# Personalized predictive modeling for patients with Alzheimer’s disease using an extension of Sullivan’s life table model

**DOI:** 10.1186/s13195-017-0302-6

**Published:** 2017-09-20

**Authors:** Eric Stallard, Bruce Kinosian, Yaakov Stern

**Affiliations:** 1 Biodemography of Aging Research Unit, Social Science Research Institute, Duke University, 2024 West Main Street, Room A102H, Durham, NC 27708-0408 USA; 2 Geriatrics and Extended Care Data Analysis Center, Philadelphia VA Medical Center, 3900 Woodland Avenue, Philadelphia, PA 19104 USA; 3 Department of Medicine, Perelman School of Medicine, University of Pennsylvania, 3615 Chestnut Street, Philadelphia, PA 19104-6021 USA; 4 Cognitive Neuroscience Division, Department of Neurology, Columbia University College of Physicians and Surgeons, 630 West 168th Street, P&S Box 16, New York, NY 10032-3702 USA; 5 Taub Institute for Research on Alzheimer’s Disease and the Aging Brain, Columbia University Medical Center, 630 West 168th Street, P&S Box 16, New York, NY 10032-3702 USA

**Keywords:** Alzheimer’s disease, Grade of Membership, Multidomain model, Longitudinal data analysis, Full-time care, Disability-free life expectancy, Disabled life expectancy, Prognostic subtype

## Abstract

**Background:**

Alzheimer’s disease (AD) progression varies substantially among patients, hindering calculation of residual total life expectancy (TLE) and its decomposition into disability-free life expectancy (DFLE) and disabled life expectancy (DLE) for individual patients with AD. The objective of the present study was to assess the accuracy of a new synthesis of Sullivan’s life table (SLT) and longitudinal Grade of Membership (L-GoM) models that estimates individualized TLEs, DFLEs, and DLEs for patients with AD. If sufficiently accurate, such information could enhance the quality of important decisions in AD treatment and patient care.

**Methods:**

We estimated a new SLT/L-GoM model of the natural history of AD over 10 years in the Predictors 2 Study cohort: *N* = 229 with 6 fixed and 73 time-varying covariates over 21 examinations covering 11 measurement domains including cognitive, functional, behavioral, psychiatric, and other symptoms/signs. Total remaining life expectancy was censored at 10 years. Disability was defined as need for full-time care (FTC), the outcome most strongly associated with AD progression. All parameters were estimated via weighted maximum likelihood using data-dependent weights designed to ensure that the estimates of the prognostic subtypes were of high quality. Goodness of fit was tested/confirmed for survival and FTC disability for five relatively homogeneous subgroups defined to cover the range of patient outcomes over the 21 examinations.

**Results:**

The substantial heterogeneity in initial patient presentation and AD progression was captured using three clinically meaningful prognostic subtypes and one terminal subtype exhibiting highly differentiated symptom severity on 7 of the 11 measurement domains. Comparisons of the observed and estimated survival and FTC disability probabilities demonstrated that the estimates were accurate for all five subgroups, supporting their use in AD life expectancy calculations. Mean 10-year TLE differed widely across subgroups: range 3.6–8.0 years, average 6.1 years. Mean 10-year DFLE differed relatively even more widely across subgroups: range 1.2–6.5 years, average 4.0 years. Mean 10-year DLE was relatively much closer: range 1.5–2.3 years, average 2.1 years.

**Conclusions:**

The SLT/L-GoM model yields accurate maximum likelihood estimates of TLE, DFLE, and DLE for patients with AD; it provides a realistic, comprehensive modeling framework for endpoint and resource use/cost calculations.

**Electronic supplementary material:**

The online version of this article (doi:10.1186/s13195-017-0302-6) contains supplementary material, which is available to authorized users.

## Background

The rate of progression of Alzheimer’s disease (AD) varies across patients, making it difficult to generate accurate estimates of the course of disease or time until specific disease endpoints for individual patients [1]. Moreover, differences in group-specific rates of progression and treatment efficacies in therapeutic trials may be confounded by individual variation in rates of progression, making it difficult to evaluate the effectiveness of randomization [2]. All of these difficulties are exacerbated by two additional factors: (1) the clinical presentation at diagnosis is highly variable over individual patients with AD—involving cognitive, functional, behavioral, psychiatric, and other symptoms; and (2) the neuropathological substrates of AD—involving neuronal dysfunction, neurodegeneration, synaptic dysfunction, cerebral atrophy, and other pathologies—differentially influence the clinical course of AD in ways that are poorly understood [3]. For example, there are no known biomarkers that closely track the progression of AD clinical signs/symptoms or uniquely identify their presence [4]. Thus, the development of a realistic, comprehensive, multidomain model of the progression of AD clinical signs/symptoms and outcomes in a well-defined cohort of patients with AD dementia could yield new insights into the process and accelerate the development of disease-modifying therapies. The need for such development was recognized in the call for new models of AD progression/outcomes in the recommendations from the 2015 National Institutes of Health AD Research Summit [5]. The model reported in this paper is intended to advance this development.

Our prior work in this area was focused on maximum likelihood estimation and cross-validation of a longitudinal Grade of Membership (L-GoM) model of AD clinical signs/symptoms [6–8]. L-GoM is a latent-variable model that resolves the difficult problem of extending multivariate latent-variable analysis from cross-sectional to longitudinal data [9, 10]. Under our prior L-GoM model, the maximum likelihood estimates of the basic parameters (i.e., the individual-specific “GoM scores”) were treated as data-based computational phenotypes [11, 12] that quantified the underlying neuropathophysiological processes giving rise to the clinical manifestations of AD recorded in the longitudinal data. In effect, the GoM scores were assumed to model the entire disease process, capturing individual differences in presentation and progression over time. The challenge in estimation was to find the optimal mapping from the data to the GoM scores.

In the present study, we modified and extended L-GoM to directly map the GoM scores to individual-specific values of residual total life expectancy (TLE) and its decomposition into disability-free life expectancy (DFLE) and disabled life expectancy (DLE), thereby obtaining a composite mapping from the data to an important set of AD timing estimates with direct clinical interpretability. To construct this composite mapping, we respecified L-GoM as an extension of Sullivan’s life table (SLT) [13].

The combined SLT/L-GoM model has four advantages over existing alternatives. First, the standard Cox model [14] assumes that covariates are fixed at baseline and hazard rates are proportional over follow-up time. Neither assumption holds for AD (e.g., *see* [7] and Fig. 4 below). Second, the time-dependent Cox model [15] resolves the first problem but introduces a new problem: temporal changes in covariates are not modeled, implying that another model (e.g., a general linear mixed model [16–18]) is needed to represent those changes. Third, cognitive, functional, behavioral, psychiatric, and other measures and their changes are correlated for patients with AD, presenting formidable technical challenges for modeling AD progression under existing approaches [2, 19]. L-GoM meets these challenges by using latent variables (GoM scores) to generate the correlations between the observed covariates [7]. Fourth, L-GoM incorporates the SLT without making any assumptions about the transitions between healthy and morbidity/disability states, a difficult task in AD modeling [19, 20].

Our prior L-GoM model used one of two separate study cohorts, Predictors 1 (*N* = 252), for estimation and the second, Predictors 2 (*N* = 254), for cross-validation [7, 8]. Several technical refinements have since been developed to meet the SLT assumptions, to incorporate fixed genetic and other data, and to optimize the model for personalized predictive applications. In the remainder of this paper, we present and apply the newly developed SLT/L-GoM model to the Predictors 2 data; characterize the most salient clinical features of the resulting subtypes; present estimates of TLE, DFLE, and DLE for the associated subgroups; and discuss how the model can be used in future research and clinical applications, including situations where the input data come from just one examination concurrent with or shortly after AD diagnosis [8].

## Methods

### Data

We used L-GoM to characterize the natural history of AD in a cohort of 229 participants (91 men, 138 women) over 21 semiannual examinations (spanning 10 years) in the Predictors 2 Study cohort (1997–2011),^1^ the second of two highly coordinated cohort studies designed to investigate the natural history of AD and to develop improved prediction models [21]. The Predictors 2 Study cohort was representative of patients with AD with mild disease severity at the time of enrollment at three specific study sites specializing in AD—Columbia University College of Physicians and Surgeons, Johns Hopkins University School of Medicine, and Massachusetts General Hospital—but was not necessarily representative of the general AD population. An essential requirement for the present analysis was that the data adequately covered the full range of patient outcomes over the 21 examinations, and this was met by selecting study sites with different disciplinary specializations: neurology at Columbia, psychiatry at Johns Hopkins, and geriatric neurobehavior at Massachusetts General Hospital. All subjects were diagnosed with “probable AD” on the basis of 1984 National Institute of Neurological and Communicative Disorders and Stroke-Alzheimer’s Disease and Related Disorders Association criteria, equivalent to “probable AD dementia” on the basis of 2011 National Institute on Aging-Alzheimer’s Association criteria [22, 23]. Date of death was reported for 186 of the 229 participants, and AD was confirmed in 96% of available postmortem diagnostic evaluations [24]. Almost all participants had mild dementia at the time of recruitment into the study. Of 226 participants with complete Clinical Dementia Rating (CDR) data [25, 26], only 9 scored > 1; of 219 participants with complete Mini Mental State Examination (MMSE) [27] results, only 4 scored < 16; and of 217 participants with complete CDR and MMSE scores, only 1 crossed both of the indicated thresholds.

A total of 119,115 distinct data points (i.e., responses) for the 229 participants were available for model fitting for the 79 covariates listed in Table 1; 6 were fixed at the intake examination, and 73 were time-varying. All 79 covariates had *p* < 0.05 based on Wilks’ chi-square test [28]; 19 others (including education) with nonsignificant *p* values were excluded from the model. The 73 time-varying covariates spanned 11 measurement domains: (1) behavior, (2) cognition, (3) functioning, (4) dependence, (5) eyesight/hearing problems, (6) acute medical treatments/conditions, (7) psychiatric/psychotic symptoms, (8) alcohol use, (9) motor signs/symptoms, (10) depression/agitation, and (11) dementia with Lewy body symptoms.

**Table 1:** Domains of measurement, instruments, and descriptions of 6 fixed and 73 time-varying covariates used in the Sullivan life table/longitudinal Grade of Membership model

No.	Domain	Instrument	Count	Description of variables
Fixed covariates				
–	–	Intake assessment	6	ApoE status, sex, age at intake, race, occupation, and years since diagnosis
Time-varying variables				
1	Behavior	CUSPAD	4	Wandering away, verbal outbursts, physical threats, and difficulty sleeping
2	Cognition	MMSE	8	MMSE completion indicator, orientation, registration, “world” backward, recall, language, and drawing
3	Functioning	BDRS	22	IADL (8 items), BADL (3 items), and personality (11 items)
4	Dependence	Dependence scale	14	Dependence scale (13 items), equivalent institutional care
5	Eyesight/hearing problems	Medical questionnaire	2	Adequate sight? Adequate hearing?
6	Acute medical treatments/conditions	Patient follow-up questionnaire	3	Admission to hospital, treatment, and had seizure?
7	Psychiatric/psychotic symptoms	CUSPAD	3	Delusions, hallucinations, and illusions
8	Alcohol use	Alcohol questionnaire	3	Beer/week, wine/week, and hard liquor/week
9	Motor signs/symptoms	UPDRS	6	Extrapyramidal signs (summary score), tremor, bradykinesia, gait, myoclonus, and rigidity
10	Depression/agitation	CUSPAD	4	Agitation, sadness/depression, depression frequency, and appetite problems
11	Dementia with Lewy body symptoms	DLB questionnaire	4	DLB questionnaire completion indicator, fluctuating cognition, and visual hallucinations

*Abbreviations: ApoE* Apolipoprotein E, *BADL* Basic activities of daily living, *BDRS* Blessed Dementia Rating Scale, *CUSPAD* Columbia University Scale for Psychopathology in Alzheimer’s Disease, *DLB* Dementia with Lewy bodies, *IADL* Instrumental activities of daily living, *MMSE* Mini Mental State Examination, *UPDRS* Unified Parkinson’s Disease Rating Scale

*See* [21] for details

### Model

Because the SLT/L-GoM model is a new synthesis of the SLT [13] and L-GoM [7, 8, 29] models, this section provides a self-contained nontechnical explanation for readers interested in understanding the model and interpreting its results. The mathematics underlying this synthesis are provided separately in Additional file 1 at a level of detail sufficient to reproduce our results and apply the model to similar data.

#### GoM scores and disease subtypes

L-GoM is actually a family of AD models distinguished by differing numbers of prognostic subtypes. For each such model, L-GoM defines one additional endpoint or terminal subtype of AD that can be approached gradually over time as disease severity increases. The progressive nature of disease severity leads to consideration of a continuum of severity scores that, in turn, give rise to the mixed membership structure [30] of the L-GoM model. Here we consider three variants of L-GoM with increasing complexity and applicability. The terms *subtype scores* and *GoM score*s are used interchangeably hereinafter.

The most basic model represents two ordered subtypes of AD: (1) mild subtype 1 (prognostic) and (2) severe subtype 2 (terminal). Under this model, a patient exhibits mild AD at the time of disease onset; some years later, the patient progresses to severe AD. In between, the patient exhibits intermediate levels of AD that can be characterized by a continuum of severity scores in the range 0–100% or, equivalently, mildness scores in the range 100–0%. Fractional scores are allowed; the sum of the scores must be held fixed at 100%. Because intake into the Predictors 2 Study necessarily occurs sometime after disease onset, the initial mildness score of a patient with AD may be < 100%, and this is the sole source of heterogeneity in initial AD presentation. AD progression occurs along this same continuum: Any two patients with the same initial mildness score have the same prognosis; alternatively, if one patient has greater initial severity than the other, then his/her prognosis is worse.

A more realistic model relaxes these assumptions using three ordered subtypes of AD: (1) mild subtype 1 (prognostic), (2) moderate subtype 2 (prognostic), and (3) severe subtype 3 (terminal). This model assigns corresponding sets of three subtype scores to patients with AD at the intake examination; the scores can be any set of three values in the range 0–100% whose sum is fixed at 100%. This model introduces an important new requirement: The ordering of the subtypes must be nonlinear in the sense that subtype 2 cannot be represented as an intermediate point between subtypes 1 and 3; if it can, then subtype 2 can be represented as a weighted mixture of subtypes 1 and 3, and the model structure will revert to the first variant. Hence, the labeling of subtype 2 as *moderate* implies only that its overall severity is greater than for subtype 1; no specific order is assumed for the severities of the individual covariates (i.e., clinical signs/symptoms). Equivalently, subtype 2 reflects additional heterogeneity in initial AD severity that cannot be represented in the more basic model; this heterogeneity is expressed most strongly at or near the time of study intake and is reflected in the measurements made at examination 1. AD progression rates differ between the prognostic subtypes, as described below.

The provisions for initial heterogeneity can be further extended using four nonlinearly ordered subtypes of AD: (1) mild subtype 1 (prognostic), (2) mild-moderate subtype 2 (prognostic), (3) moderate subtype 3 (prognostic), and (4) severe subtype 4 (terminal). Their corresponding sets of subtype scores are in the range 0–100% whose sum is fixed at 100%. The requirement for nonlinear ordering means that no subtype can be represented as an intermediate point between any other pair of subtypes; if it can, then that subtype can be represented as a weighted mixture of the other pair of subtypes, and the model structure will revert to one of the simpler variants. As above, the labeling of subtypes 1–3 as *mild*, *mild-moderate*, and *moderate* implies only that the overall severities are in increasing order; no specific order is assumed for the severities of the individual covariates.

Maximum likelihood estimation requires that the study sample be (1) sufficiently heterogeneous at initial intake to fully span the range of the initial prognostic subtypes and (2) followed sufficiently long (i.e., until death for most subjects) to identify different rates and types of progression from the initial presentation to severe AD. The Predictors 1 and 2 studies met these requirements. Our previous analysis of the Predictors 1 Study overwhelmingly supported a model with four subtypes [7], so this model was used for the present analysis of the Predictors 2 Study.

#### AD progression

AD progression is represented in L-GoM as irreversible movement in the GoM score continuum away from the prognostic subtypes toward the terminal subtype, which is implemented by allowing the GoM scores to change from one examination to the next (while always summing to 100%). The simplest type of AD progression is movement away from a specific prognostic subtype toward the terminal subtype. The assumed ordering of the subtypes prohibits any movement from a higher-numbered to a lower-numbered subtype. For our model, which incorporates four subtypes, the progression away from subtype 1 may include movement toward subtypes 2 and/or 3 before ultimately heading toward subtype 4. The cumulative transitions from the prognostic subtypes to the terminal subtype may be incomplete in the sense that the terminal subtype score for the last examination may be < 100%.

L-GoM assumes that the paths or trajectories in the GoM score continuum extending from each prognostic subtype toward the terminal subtype are deterministic and are fundamental properties of the AD subtypes. If a given patient scores 100% on a given prognostic subtype, then the patient’s AD trajectory is fully determined. We refer to these as the *pure-subtype trajectories*.

L-GoM uses the pure-subtype trajectories as the basis of its model of AD progression among patients with mixed GoM scores at the initial examination (i.e., sets of subtype scores for which no individual score equals 100%). For each such patient, the corresponding trajectory is modeled as a weighted combination of the pure-subtype trajectories using his/her initial GoM scores as weights. Each GoM score weighted combination of the pure-subtype trajectories defines a unique deterministic trajectory from the initial point in the GoM score continuum toward the terminal subtype, generating a deterministic sequence of time-varying GoM scores in one-to-one correspondence with the study examinations. We refer to these as the *GoM score trajectories*.

This model of AD progression ensures that the range of the time-varying GoM scores contracts over time as patients move away from the prognostic subtypes toward the terminal subtype. Hence, the data from examination 1 should be given greater weight in the maximum likelihood estimation procedure because that is the only examination for which the GoM scores can fully span the range of the prognostic subtypes. The weighting procedure for examination 1 in the present study ensured that the L-GoM estimates for all examinations were consistent with the corresponding cross-sectional GoM estimates for examination 1 alone (*see* Additional file 1: Appendix).

#### Outcome probabilities and *λ* parameters

Application of maximum likelihood to longitudinal data such as that in the Predictors 2 Study requires that all variables used in the analysis be coded as discrete categorical variables (e.g., 0 or 1 for binary responses). For each AD subtype, the probability of each possible response for each measured variable was estimated under the assumption that the probability was constant over all examinations. These probabilities are referred to as the *pure-subtype probabilities* or *λ parameters* (because they are denoted using the symbol λ in the mathematical formulas). For each subject, the probability of each given response for each measured variable was determined at each examination as a time-varying GoM score weighted combination of the corresponding pure-subtype probabilities.

#### Sullivan’s life table

Because the L-GoM model can effectively predict the probability of any event occurring at any time during follow-up, it is ideal for constructing life table survival models where death is the endpoint event and TLE is the statistic of interest. Here, we extended L-GoM to allow DFLE and DLE to be estimated using the SLT method [13]. Sullivan [13] showed that the overall time of survival (i.e., TLE) could be decomposed into the time spent in healthy (i.e., DFLE) and morbidity/disability (i.e., DLE) states by multiplying the overall survival function value at each examination by the respective morbidity/disability prevalence rate, summarizing the results using standard life table calculations. Imai and Soneji [31] showed that (1) the SLT method was statistically optimal in applications to longitudinal cohort data, justifying its use in medical follow-up studies; and (2) DFLE and DLE can be estimated without making any assumptions about the underlying transition rates.

In the present study, the L-GoM model allowed an overall survival function to be generated for each subject as the product of the conditional survival probabilities defined for each 6-month interval between adjacent examinations. Disability was defined as the need for full-time care (FTC), a major disability endpoint in AD research [32–34] and the highest outcome category for the *equivalent institutional care* variable. TLE was generated as the area under the subject’s overall survival function. DFLE and DLE were generated as the area under the subject’s disability-free and disabled survival functions, using the SLT method to apply his/her estimated FTC disability-free and FTC disability probabilities to the overall survival function at each examination to generate the component survival functions.

#### Goodness of fit

Although the SLT calculations were conducted separately for each individual subject in the study, it was not feasible to assess the goodness of fit of the model at the individual level. Instead, we divided the 229 study participants into five relatively homogeneous subgroups (i.e., so-called rational subgroups [35]) on the basis of their GoM scores on the four AD subtypes identified by the L-GoM model. The five subgroups comprised those individuals predominantly expressing one subtype at the initial examination (i.e., with exactly one GoM score > 50%) plus a residual “subgroup 0” (i.e., with no GoM score > 50%). We employed the SLT/L-GoM model to estimate total, disabled, and disability-free 10-year survival functions and life expectancies, overall and by subgroup, using FTC to define disability. We assessed the goodness of fit of model-based to observed values by subgroup for the 21 examinations using pointwise and simultaneous confidence bands [36] for the overall survival curves and pointwise confidence bands for the FTC probabilities.

## Results

### Characterization of subtypes

The four disease subtypes and their trajectories were determined using procedures detailed in Additional file 1: Table A.2 (see Additional file 1: Appendix) displays the Kullback-Leibler information statistics [37] used to assess the relative importance of each covariate for each of the four AD subtypes. Additional file 1: Table A.3 displays the corresponding λ parameters. Additional file 1: Table A.5 displays the corresponding pure-subtype trajectories. The statistics in Additional file 1: Tables A.2 and A.3 are summarized in Table 2 below in a form that characterizes the four subtypes according to the size and direction of the effects exhibited by the 35 most salient covariates in the model. Each combination of subtype and covariate was coded as exhibiting low (L), medium (M), or high (H) severity, but only if the associated λ parameters exhibited relatively large differences from the corresponding marginal probabilities for randomly selected patients with AD (i.e., computed by averaging over all completed examinations, not just examination 1). Operationally, the L-M-H designation was made only if the Kullback-Leibler information statistic [37] was > 0.50 (*see* Additional file 1: Appendix); otherwise, the covariate severity remained unclassified for the subtype. The M designation was used only for covariates with three or more severity levels, and only if an unambiguous H or L designation could not be made.

**Table 2:** Symptom severity for Alzheimer’s disease subtypes on the 35 most salient covariates and 5 salient summary measures

				Subtype			
*j*	Domain	Name	Description	1	2	3	4
Salient covariates							
8	1	PP44	Verbal outbursts			H	
12	2	Orientation_RC	MMSE: sum of orientation variables	L			H
17	2	SP41B	MMSE: intersecting pentagons				H
18	3	NN01	Patient trouble with chores	L			H
19	3	NN02	Patient trouble handling money	L			H
20	3	NN03	Patient trouble remembering lists	L			
21	3	NN04	Patient trouble around house				H
22	3	NN05	Patient trouble around neighborhood	L	L		H
23	3	NN06	Patient trouble recognizing place	L	L		H
24	3	NN07	Patient trouble remembering things	L			
25	3	NN08	Patient dwells in the past			H	
27	3	NN10	Patient dressing	L			H
28	3	NN11	Patient bladder and bowel control				H
29	3	NN12	Increased rigidity			H	
30	3	NN13	Increased egocentricity			H	
31	3	NN14	Impairment of regard for feelings of others			H	
32	3	NN15	Coarsening of affect			H	
33	3	NN16	Impairment of emotional control			H	
35	3	NN18	Diminished emotional responsiveness	L			
36	3	NN19	Sexual misdemeanor			H	
37	3	NN20	Hobbies relinquished	L			
40	4	RR01	Needs reminders	L			
41	4	RR02	Needs help to remember	L			
43	4	RR04	Needs household chores done	L			
44	4	RR05	Needs watching when awake	L	L		H
45	4	RR06	Needs to be escorted when outside	L	L		H
46	4	RR07	Needs to be accompanied bathing/eating	L	L		H
47	4	RR08	Needs to be dressed/washed/groomed				H
48	4	RR09	Needs to be taken to toilet				H
51	4	RR12	Needs to wear diaper/catheter				H
53	4	RR15	Equivalent institutional care	L		M	H
61	7	DELUSION	Delusions			H	
67	9	EPSXX	Extrapyramidal symptoms				H
73	10	AGITATION	Agitation			H	
74	10	SAD	Sadness/depression			H	
Total H/L				17	5	11	17
Salient Summary Measures							
—	—	QQ01_RC	CDR rating	L	L		H
—	2	SP51_RC	MMSE score	L			H
—	3	NNTOT_RC	BDRS score	L	L	M	H
—	4	RR14	Dependence scale score	L	L	M	H
—	7	PSYCHSX	Psychiatric symptoms			H	
Total H/L				4	3	1	4

*Abbreviations: BDRS* Blessed Dementia Rating Scale, *CDR* Clinical Dementia Rating, *LMH* Low, medium, or high severity, MMSE Mini Mental State Examination

*j* denotes variable number. LMH designations indicate the direction and strength of the symptom severity for the indicated subtypes. LMH designations are provided for covariates with positive Bayesian information criterion statistics in Additional file 1: Table A.2, but only if the corresponding Kullback-Leibler information statistics exceed 0.50. These conditions identified the 35 most salient covariates shown above. The summary measures were processed using conditional maximum likelihood estimation procedures that did not impact the estimated Grade of Measurement scores. In three cases, the conditions for assigning H or L were met, but the effect involved a restricted set of intermediate severity levels, which were coded as M

The severity patterns in Table 2 show that the subtypes are quite distinct. Subtype 1 had the largest number of covariates exhibiting low severity (*n* = 17). Subtype 2 had low severity for 5 of the same 17 covariates. Subtype 3 showed no overlap in severity designations with subtype 1 or 2. Subtypes 3 and 4 had high severity on 11 and 17 covariates, respectively, but the high-severity designations did not overlap. Subtypes 3 and 4 had medium and high severity, respectively, on the *equivalent institutional care* variable (number 53, the only covariate for which both subtypes had severity codes), on the basis of their respective use of *adult home care* vs. FTC. *Equivalent institutional care* was the top-ranked covariate (based on *p* values).

Subtype 4, representing the terminal endpoint of the AD process, had high patient dependence, moderate extrapyramidal symptoms, poor cognition, and highly impaired functioning. Subtype 3 was the only subtype with any severity designations on items within domains 1, 7, and 10 (behavior, psychiatric/psychotic symptoms, and depression/agitation), and it was the only subtype with high severity on the personality items within domain 3 (Blessed Dementia Rating Scale [BDRS]). Hence, psychiatric and behavioral symptoms, personality changes, and depression were strongly exhibited only by subtype 3. Subtype 1 had low initial severity on 17 items, slow AD progression (Additional file 1: Table A.5), and the best prognosis. Subtype 2 had low initial severity on five items, fast AD progression (Additional file 1: Table A.5), and the worst prognosis. Compared with subtype 1, subtype 2 was more likely to be female, be older, score lower on the MMSE, and be homozygous for the apolipoprotein E (ApoE) ε4 allele.

We evaluated the assumption that overall severity increased over subtypes by applying the LMH severity coding procedure to five summary measures: CDR [25], MMSE score [27], BDRS score [38, 39], Dependence Scale score [40], and psychiatric symptoms [41, 42] (Table 2). The severity codes for subtypes 1 and 2 were indistinguishable on four of the five summary measures, the exception being MMSE, with subtype 1 coded low (L) and subtype 2 unclassified. The severity for subtype 3 was higher than for subtype 1 on all five summary measures and higher than for subtype 2 for all but MMSE. Thus, the results for the summary measures exhibited monotonic severity patterns, which confirmed the assumed overall ordering of the subtypes; moreover, taken as a set, the severity codes were consistent with the labeling of the subtypes as *mild*, *mild-moderate*, *moderate*, and *severe*.

A finding of great prognostic significance was that the rate of AD progression was substantially faster for subtype 2 than for subtype 3, which, in turn, was substantially faster than for subtype 1 (Additional file 1: Table A.5). The ordering reversal between subtypes 2 and 3 was maintained throughout the 10-year study period, with the fourth component of the pure-subtype trajectories reaching final values of 0.58, 0.99, and 0.93, respectively, for subtypes 1–3.

**Fig. 1:**
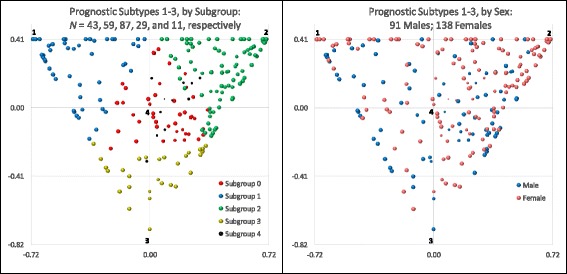
Scatterplot of the Grade of Membership (GoM) scores for examination 1 by subgroup and sex. Within each chart, subtypes 1–3 are located at the vertices of each triangular region, starting from the upper left side, in clockwise order. The GoM score continuum is triangular because GoM scores locate individuals at or between the indicated subtype vertices. The origin of the coordinate system is located at the centroid of each triangular region; the coordinate axes are scaled to reproduce the distance of $\sqrt{2}$ GoM score units between all pairs of vertices. Subtype 4 is hidden 1.15 GoM score units directly behind the origin of the coordinate system. Each bubble represents the estimated GoM scores for one subject, except at the vertices for subtypes 1 and 2, where the bubbles represent ten (seven males, three females) and three (female) subjects, respectively; the vertices for subtypes 3 and 4 are unoccupied. The location of each bubble is determined by the subject’s GoM scores on subtypes 1–3. The area of each bubble declines linearly with the GoM score on subtype 4, such that the bubble vanishes at the subtype 4 vertex. This is why the bubbles for subgroup 4 are much smaller than for the other subgroups. The GoM scores for the 229 subjects are listed in Additional file 1: Table A.6

The relationships between the three prognostic subtypes and the five subgroups can be visualized using the scatterplot on the left side of Fig. 1. Except for ten subjects located at the subtype 1 vertex and three subjects at the subtype 2 vertex, the scatterplot shows no evidence of clustering of the subjects. The subtype 3 vertex was unoccupied (because the highest GoM score for subtype 3 was 0.89); moreover, the nearby regions were thinly populated. The five subgroups are color-coded in Fig. 1; they were constructed to be cleanly separated and substantially more homogeneous than the overall sample (*see* Additional file 1: Table A.7). In contrast, the scatterplot on the right side of Fig. 1 shows that, although the sex effect was highly significant (*p* = 0.00007) (Additional file 1: Table A.2), males and females were distributed throughout the plot with no clean separation. Females, however, had their highest relative concentrations near subtype 2 and between subtypes 1 and 2. Males had their highest relative concentrations between subtypes 1 and 3 and between subtypes 2 and 3; seven males were located at the subtype 1 vertex.

### Observed vs. estimated survival

**Fig. 2:**
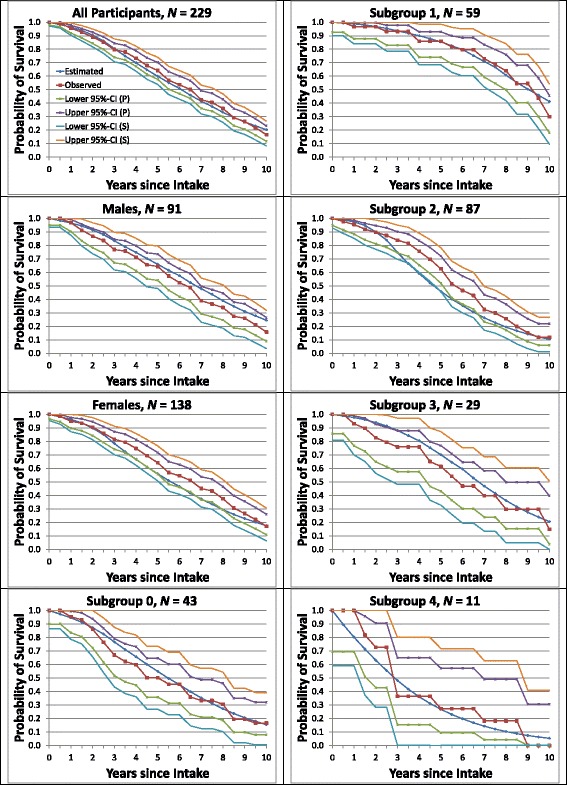
Observed vs. estimated average survival. Curves for average survival are shown separately for all participants and for males, females, and subgroups 0–4, with pointwise (P) and simultaneous (S) 95% confidence bands. Section 1.9 of Additional file 1: Appendix describes the calculations of the plotted values and their confidence bands

The basic 6-month mortality probabilities (λ parameters) were 0.6% and 16.0% for subtypes 1 and 4, respectively, and zero for subtypes 2 and 3 (Additional file 1: Table A.3), indicating that individual mortality was determined by subtypes 1 and 4 alone, constrained to the range 0–16.0%, with the rate at time *t* primarily determined by an individual’s time-varying GoM score on subtype 4 (maximum 16.0%). Figure 2 displays the observed vs. estimated survival curves for all participants, males and females, and subgroups 0–4. The estimates were almost all within the simultaneous 95% confidence bands around the observed values, the sole exceptions being subgroup 2 at 3.5–4.5 years.

### Observed vs. estimated need for full-time care

**Fig. 3:**
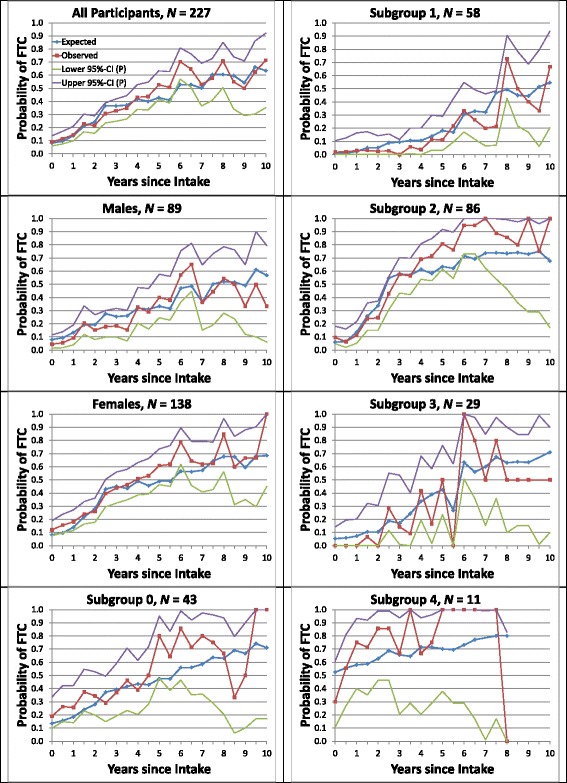
Observed vs. estimated average need for full-time care (FTC) among survivors at each examination. Average probabilities of FTC are shown separately for all participants and for males, females, and subgroups 0–4, with pointwise (P) 95% confidence bands. Section 1.9 of Additional file 1: Appendix describes the calculations of the plotted values and their confidence bands

FTC represents “around-the-clock supervision of personal care, safety, or medical care” [40], as measured by the top-ranked covariate, *equivalent institutional care*. The basic disability probability (λ parameter) was 83.2% for subtype 4 and zero for subtypes 1–3 (Additional file 1: Table A.3), with the rate at time *t* fully determined by an individual’s time-varying GoM score on subtype 4. Figure 3 displays the observed vs. estimated FTC rates for all participants, males and females, and subgroups 0–4. The estimated FTC rates increased over time for both sexes and all subgroups. The fits to the observed FTC rates were excellent; all but three estimated values (both sexes and females at 6 years and subgroup 2 at 6.5 years) were within the pointwise 95% confidence bands around the observed values.

### Survival functions and life expectancies

**Fig. 4:**
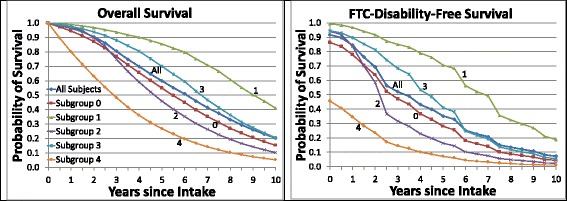
Overall and full-time care (FTC) disability-free average survival. The plots in the *left panel* are the estimated curves for average survival shown in Fig. 2; the plots in the *right panel* are the estimated average survival curves for FTC disability-free survival, calculated using Sullivan’s method to apply each individual’s estimated FTC disability-free probabilities to his/her overall survival function values at each examination. Average survival curves are shown for all participants and subgroups 0–4. Section 1.9 of Additional file 1: Appendix describes the calculations of the plotted values

Figure 4 displays the overall survival curves (left side) and the corresponding FTC disability-free survival curves (right side) for all participants and subgroups 0–4. Both sets of survival curves decreased monotonically over time and were strongly separated by subgroup. Subgroup 2 crossed over subgroup 0 at 2.5 and 1.5 years, respectively, implying that the underlying hazards were not proportional.

Table 3 displays the means, 95% CIs, and ranges of the 10-year TLEs, DFLEs, and DLEs for all participants and subgroups 0–4, assuming that survival beyond year 10 (examination 21) was censored. The TLEs and DFLEs are the areas under the respective plots in Fig. 3. Whereas the DFLEs varied widely across subgroups, the DLEs did not; the relative variation of the TLEs was intermediate between that of the DFLEs and DLEs. Mean 10-year TLE differed widely across subgroups: range 3.57–7.98 years, average 6.09 years. Mean 10-year DFLE differed relatively even more widely across subgroups: range 1.23–6.47 years, average 4.03 years. Mean 10-year DLE was relatively much closer: range 1.51–2.34 years, average 2.06 years. Subgroup 4 was distinguished by its relatively short DFLE (1.23 years) and subgroup 1 by its relatively long DFLE (6.47 years). Among the subgroups most closely related to the prognostic subtypes (1–3), subgroup 2 had the shortest DFLE and the longest DLE at 2.85 and 2.33 years, respectively.

**Table 3:** Ten-year Sullivan method life expectancies by subgroup, with disability defined as need for full-time care

	*N*	TLE		DFLE		DLE	
	(1)	(2)		(3)		(4)	
		Mean (95% CI)					
Subgroup							
1	59	7.98	(7.78–8.17)	6.47	(6.20–6.74)	1.51	(1.44–1.58)
2	87	5.18	(5.07–5.29)	2.85	(2.72–2.98)	2.33	(2.31–2.35)
3	29	6.60	(6.26–6.94)	4.53	(4.12–4.93)	2.08	(2.01–2.15)
4	11	3.57	(3.38–3.77)	1.23	(1.03–1.44)	2.34	(2.32–2.35)
0	43	5.64	(5.36–5.93)	3.47	(3.15–3.79)	2.18	(2.13–2.22)
Total	229	6.09	(5.90–6.28)	4.03	(3.80–4.27)	2.06	(2.01–2.11)
Maximum	229	7.98	(7.78–8.17)	6.47	(6.20–6.74)	2.34	(2.32–2.35)
Minimum	229	3.57	(3.38–3.77)	1.23	(1.03–1.44)	1.51	(1.44–1.58)
Ratio	229	2.23	(2.06–2.42)	5.24	(4.30–6.56)	1.55	(1.47–1.64)

*Abbreviations: DFLE* Disability-free life expectancy, *DLE* Disabled life expectancy, *TLE* Total life expectancy

All life expectancy (LE) estimates are 10-year LEs. Survival beyond 10 years was censored. TLE is the sum of DFLE and DLE. The 95% CIs reflect the variation between individuals of the indicated estimates; other sources of variation were assumed negligible. Minima and maxima are for subgroups; CIs for ratios are based on CIs for the corresponding minima and maxima

## Discussion

This study provides the first published estimates of the L-GoM extension of the SLT model. Our motivation for this extension was fourfold. First, our analysis supports the hypothesis that patients with AD are heterogeneous in initial presentation and in rates of progression [1], implying that adequate characterization of the clinical course of AD requires a parsimonious multivariate latent-variable model such as L-GoM [10]. Second, the ability to directly map the GoM scores to TLE, DFLE, and DLE focuses attention on these readily understood, familiar metrics. This contrasts with existing factor analytic models [9] that cannot incorporate the SLT model and cannot extract TLE, DFLE, and DLE from patient-level longitudinal data [10, 19]. Third, predictions of TLE, DFLE, DLE, and associated survival curves for many types of disability, especially FTC, are central to important decisions in AD treatment and patient care; they represent information that patients with AD, their families, and caregivers want to know. Fourth, the L-GoM extension of the SLT model can be used to assess the effects of treatment on disability-free and disabled survival. Lifetime costs can be calculated by combining estimated survival curves and cost functions for selected disability measures, implying that the SLT/L-GoM model can be used as a realistic, comprehensive modeling framework for endpoint and resource use/cost calculations for individual patients with AD and subgroups. The appendix in Additional file 1 provides all parametric estimates needed for hypothesis generation and further exploration of AD using the SLT/L-GoM model (Additional file 1: Tables A.3–A.6).

Our prior L-GOM model was based on longitudinal data from the Predictors 1 Study cohort [7]. Subsequently, we forward-applied that model to baseline data from the Predictors 2 Study cohort and showed that it accurately predicted times to FTC, nursing home care, and death [8]. Although that model was a major advancement, we updated the L-GoM model in this study for four reasons, described below.

First, several advances were made to the L-GoM estimation software, including more accurate representations of the effects of fixed covariates such as sex, race, age, occupation, and ApoE status, using only information from examination 1, which satisfies Drachman’s [43] call for prognostic covariates that are independent of initial severity. Race was dichotomized as white vs. nonwhite because of an insufficient sample size to support further stratification. Only 17 of the 229 subjects were nonwhite. Education was evaluated as a potential covariate, but it did not contribute significantly to the model (*p* = 0.30). Only 8 of the 229 subjects had less than 9 years of education. Another advance was the weighted maximum likelihood estimation procedure, which took into account the unique status of examination 1 as the only examination that spans the full range of the prognostic subtypes (Fig. 1). The algorithm for generating the excess weight for examination 1 used the Akaike information criterion procedure [44] to limit the loss of fit for examinations 2–21 and maintain the accuracy of the estimated pure-subtype trajectories extending from the prognostic subtypes to the terminal subtype (Additional file 1: Table A.5).

Second, the Predictors 2 Study had several advantages over Predictors 1, including the availability of ApoE genotype at examination 1. The updated model used pooled male/female data; the prior model used sex-specific data. The pooled data yielded more accurate parametric estimates, which revealed significant sex and ApoE genotype differences.

Third, the updated model incorporated several new covariates and refined versions of others, including individual items from the BDRS [38, 39] and MMSE [27], individual motor signs, and depression measures. These changes contributed significantly to the characterization of prognostic subtype 3, allowing the clinical presentation of this subtype to be clearly distinguished from that of subtype 2 (Table 2). Six summary measures were processed using conditional maximum likelihood estimation procedures that did not impact the estimated GoM scores. They were ranked as follows (based on *p* values) (Additional file 1: Table A.2): *dependence scale score* [40], *BDRS score* [38, 39], *CDR rating* [25], *MMSE score* [27], *psychiatric symptoms* [41, 42], and *total weekly alcohol consumption* [45]; the top five were included in Table 2.

Fourth, the updated model generated maximum likelihood estimates of the TLEs, DFLEs, and DLEs for individual patients with AD and for the aggregates of individual patients in subgroups 0–4 [46]. We assessed the validity of the updated model by showing that the GoM subgroups all had very accurate predictions of FTC and mortality at or following each of the 21 examinations (Figs. 2 and 3). This assessment is new; it was not done for the prior model. It follows that the updated model generates even more accurate, valid, and informative representations of AD progression than the prior model. The updated model also explains why our prior Cox analyses [14, 32] were successful in predicting FTC and mortality. Both endpoints were strongly associated with subtype 4, the terminal subtype of the L-GoM process. Hence, any covariate strongly associated with subtype 4 should work well as a predictor in the Cox model [14] (e.g., those with high severity for subtype 4 in Table 2).

By creating relatively homogeneous rational subgroups [35] of patients, such as subgroups 0–4 in Predictors 2, we could demonstrate that the estimated survival closely matched the actual survival for any homogeneous subgroup. The goodness-of-fit plots in Figs. 2 and 3 showed that the survival and disability (need for FTC) variables were well-estimated for all subgroups and observation times. These variables were but 2 of the 80 variables in the final model; they were treated like the other 78 variables so that the results could be representative of the entire AD process. Alternative measures of disability could be incorporated into these calculations on the basis of any of the disability-related covariates included in the study. Zehna’s theorem [46] ensures that the resulting individual-specific TLEs, DFLEs, and DLEs are maximum likelihood estimates (*see* Additional file 1). Forward application of the model to other prospective datasets will be required to further validate its statistical optimality and general applicability; these analyses are underway.

The weighted maximum likelihood estimation procedure ensures that the GoM score estimates from examination 1 alone are of high quality. It follows that maximum likelihood estimates of patient-specific GoM scores and survival curves can be generated conditionally on the parameters presented in Additional file 1: Tables A.3 and A.4 using input data from just one examination concurrent with or shortly after AD diagnosis [8]. Hence, the SLT/L-GoM model could be used for personalized predictive modeling for new patients with AD—with important caveats. Accurate estimation of the individual survival curves and associated TLEs, DFLEs, and DLEs is not equivalent to precise estimation of observed times to specific disease endpoints; the timing is inherently stochastic. This stochasticity could be handled if, in addition to the mean estimates, individual patients with AD, or their physicians, families, and caregivers, were supplied with estimates of key quantiles (e.g., 10th, 25th, 75th, and 90th percentiles) of the individualized survival curves. Our findings in the present study indicate that the DFLEs differed widely as a function of GoM subgroup at the initial visit, whereas the DLEs were relatively much closer (Table 3). Hence, the variability of the TLEs is attributable primarily to the variability of the DFLEs, underscoring the importance of DFLE in prognostic applications.

Our analyses have several important limitations. First, the Predictors 2 Study cohort was a nonrandom collection of participants enrolled at three specific study sites specializing in AD, which may limit the generalizability of our results [21]. Second, the full SLT/L-GoM model can only be estimated using longitudinal data with extensive sets of time-varying covariates at each examination. However, if such data have already been assembled, then SLT/L-GoM provides a highly efficient mode of analysis. Third, the assumed form and temporal structure of L-GoM may be oversimplified, reflecting the limited sample size available in the Predictors 2 Study, which required just two nonzero λ parameters to generate the entire ensemble of individual survival curves and just one λ parameter for the corresponding FTC curves. Subsequent applications may require additional λ parameters or more subtypes.

There are several other potential applications of L-GoM and its SLT extension. One would use L-GoM to determine expected progression in drug and placebo groups in clinical trials evaluating the effectiveness of randomization prior to the trial and comparing modeled vs. actual progression in the drug group after the trial [47]. Alternatively, L-GoM could be used to explore how the clinical symptoms/signs captured by it correlate with measured AD biomarkers, such as by testing concurrent and lagged associations of biomarkers and time-varying GoM scores, associations that could elucidate the connections between DFLE/DLE and the neuropathology of AD [48, 49]. With such applications, the model and its results have the potential to stimulate rapid progress in the fight against AD.

## Conclusions

The objective of the present study was to assess the accuracy of the estimates produced by the SLT/L-GoM model. This required that we generate for the first time a comprehensive, individualized multidomain model of AD progression covering the first 10 years following study intake and incorporating a composite mapping leading directly from the longitudinal data to the individual-specific TLEs, DFLEs, and DLEs. The substantial heterogeneity in initial patient presentation and AD progression was captured using three clinically meaningful prognostic subtypes (subtypes 1–3) and one terminal subtype (subtype 4) exhibiting highly differentiated symptom severity on 7 of the 11 measurement domains in the model (Table 2). The rates of progression for subtype 2 (mild-moderate severity at examination 1) were found to be substantially faster than for subtype 3 (moderate severity at examination 1), underscoring the need to distinguish these subtypes in clinical prognostication. The mixed membership property of the model was used to define five relatively homogeneous but diverse patient subgroups, four of which (1–4) had high GoM scores on the respective prognostic/terminal subtypes, with the fifth defined as a residual subgroup. The model yielded accurate maximum likelihood estimates of TLE, DFLE, and DLE and associated survival and disability probabilities for all five subgroups. Thus, the model provides a realistic, comprehensive framework for endpoint and resource use/cost calculations for patients with AD.

## Additional file

Additional file 1: This file has two sections and an Appendix. Section 1 presents a high-level overview of the mathematics of the L-GoM extension of the Sullivan life table model. Section 2 presents an associated irreversible disability model with two supplementary tables (Tables S1 and S2) and one figure (Figure S1). The appendix expands on the technical details of the mathematics for both sections and contains Tables A.1–A.7. (PDF 935 kb)

## Abbreviations

AD: Alzheimer’s disease
ApoE: Apolipoprotein E
BADL: Basic activities of daily living
BDRS: Blessed Dementia Rating Scale
CDR: Clinical Dementia Rating
CUSPAD: Columbia University Scale for Psychopathology in Alzheimer’s Disease
DFLE: Disability-free life expectancy
DLB: Dementia with Lewy bodies
DLE: Disabled life expectancy
FTC: Full-time care
IADL: Instrumental activities of daily living
LE: Life expectancy
L-GoM: Longitudinal Grade of Membership
LMH: Low, medium, or high severity
MMSE: Mini Mental State Examination
SLT: Sullivan’s life table
TLE: Total life expectancy
UPDRS: Unified Parkinson’s Disease Rating Scale

## Footnotes

1 Our prior analysis of Predictors 2 had *N* = 254 [7], but the study was ongoing. The final sample size was 267; of these, 38 were diagnosed as having Lewy body dementia at the intake examination and were excluded from the present analysis.
