# Supplementary material for: Personalized predictive modeling for patients with Alzheimer’s disease using an extension of Sullivan’s life table model
Source: Alzheimers Res Ther. 2017 Sep 20;9:75. doi: 10.1186/s13195-017-0302-6 (PMC5607490; doi:10.1186/s13195-017-0302-6)
Supplement: Additional file 1: — This file has two sections and an Appendix. Section 1 presents a high-level overview of the mathematics of the L-GoM extension of the Sullivan life table model. Section 2 presents an associated irreversible disability model with two supplementary tables (Tables S1 and S2) and one figure (Figure S1). The appendix expands on the technical details of the mathematics for both sections and contains Tables A.1–A.7. (PDF 935 kb) [file 13195_2017_302_MOESM1_ESM.pdf]

SUPPLEMENTARY MATERIAL

Personalized Predictive Modeling for Alzheimer's Disease Patients Using an Extension of Sullivan's Life Table Model

This Supplement has two sections and an Appendix. Section 1 presents a high-level overview of the mathematics of the L-GoM extension of the Sullivan life table model. Section 2 presents an associated irreversible disability model that yields a unique and simple decomposition of the DFLEs and DLEs that clarifies the relationship of the Sullivan life expectancies to other life-expectancy calculations that might be obtained from application of multi-state competing-risk models [1]. The Appendix expands on the technical details of the mathematics for both sections, describes the weighted maximum likelihood estimation procedure used in Section 1, and presents the estimated parameters and related test statistics for the model in the main text.

1. OVERVIEW OF SLT/L-GOM MODEL

1.1 L-GoM Extension of Sullivan's Model

Under Sullivan's model [2], the ω-period (e.g., 10-year) DLE is computed for each individual i in the study cohort for discrete equally-spaced follow-up time intervals (e.g., half-years in Predictors 2), indexed by t (or τ), $t = 0,1,\ldots,\omega$, using:

$$e_{\mathrm{D}i} = \frac{\pi_{i0}}{2} + \sum_{t=1}^{\omega-1}\left(\prod_{\tau=0}^{t-1} p_{i\tau}\right)\pi_{it} + \left(\prod_{\tau=0}^{\omega-1} p_{i\tau}\right)\frac{\pi_{i\omega}}{2}, \tag{S.1}$$

where $p_{i\tau}$ is the conditional probability of survival for individual i for the unit time interval beginning at time τ, given survival to time τ; π_{it} is the conditional probability that individual i meets the selected morbidity/disability criteria at time t, given survival to time t; and $e_{\mathrm{D}i}$ is the DLE for individual i. The initial and terminal division by 2 derives from the trapezoidal integration rule [3]. Disability-free (active) life expectancy (DFLE; equivalently ALE [4]), denoted $e_{\mathrm{A}i}$, is computed similarly with $\pi_{it}^{c} = 1 - \pi_{it}$ replacing π_{it} in eqn. (S.1):

$$e_{\mathrm{A}i} = \frac{(1-\pi_{i0})}{2} + \sum_{t=1}^{\omega-1}\left(\prod_{\tau=0}^{t-1} p_{i\tau}\right)(1-\pi_{it}) + \left(\prod_{\tau=0}^{\omega-1} p_{i\tau}\right)\frac{(1-\pi_{i\omega})}{2}. \tag{S.2}$$

Total life expectancy (TLE), denoted as e_i, is the sum of DFLE and DLE, i.e., $e_i = e_{\mathrm{A}i} + e_{\mathrm{D}i}$.

It follows from Zehna's theorem [5] that the maximum likelihood estimator (MLE) of $e_{\mathrm{D}i}$ is a function, of the form in eqn. (S.1), of the MLEs of $p_{i\tau}$ and π_{it}. The MLE of $e_{\mathrm{A}i}$ is computed similarly using eqn. (S.2). The contribution to the likelihood made by individual *i* is:

$$L_i = \prod_{t=0}^{T_i-1} p_{it} \times \left(p_{iT_i}\right)^{\varepsilon_{iT_i}} \left(1-p_{iT_i}\right)^{1-\varepsilon_{iT_i}} \times \prod_{t=0}^{T_i} \left(\pi_{it}\right)^{\delta_{it}} \left(1-\pi_{it}\right)^{1-\delta_{it}} , \qquad \text{(S.3)}$$

where T_i is the time of the last examination for individual *i*; ε_{iT_i} is coded 1 if individual *i* was alive at time T_i+1 and 0 if dead at that time; and δ_{it} is coded 1 if individual *i* was disabled at time *t* and 0 if free of disability at that time.

Estimation of the parameters in eqn. (S.3) uses the L-GoM extension of the SLT which is summarized in the following paragraphs (see the Appendix for full details).

Let $X(i, j, t)$ be the jth polychotomous categorical covariate, *j* = 1,…, *J*, for individual *i* at time *t*, with discrete outcomes denoted by $x(i, j, t)$, with corresponding outcome probabilities denoted by $\pi_{it}^{jx(i,j,t)}$, where the superscripts represent indexes, not exponents. Define *K* as the number of latent subtypes (equivalently "pure types") in a standard GoM model [6-8] with individual-specific parameters g_{ik}, $k=1,\ldots,K$, constituting the set of *K* GoM scores for individual *i*. Define $\lambda_k^{jx(i,j,t)}$ as the latent probability for subtype *k* and covariate *j* of the discretely-coded outcome $x(i, j, t)$ actually observed for individual *i* on covariate *j* at time *t*. Then, L-GoM incorporates eqn. (S.3) into the overall likelihood as follows:

$$L = \prod_{i=1}^{N} \prod_{j=0}^{J} \prod_{t=0}^{\Theta_{ij}} \left(\sum_{k=1}^{K} g_{ik} \sum_{h=k}^{K} v_{kht} \lambda_h^{jx(i,j,t)} \right), \qquad \text{(S.4)}$$

where *N* is the sample size; $j=0,1,\ldots,J$ indexes the "internal" variables of the L-GoM model; $\Theta_{ij}=T_i$ if covariate *j* is time-varying and $\Theta_{ij}=0$ if covariate *j* is fixed; and the survival components of eqn. (S.3) are represented via suitable extensions of $X(i, j, t)$, $x(i, j, t)$, and $\lambda_k^{jx(i,j,t)}$ using the special index value $j=0$. Designation of a variable as "internal" means that the associated λ-parameters are estimated simultaneously with the *g*- and *v*-parameters using eqn. (S.4); all other variables are designated as "external". The outer sum in eqn. (S.4) generates the outcome probabilities $\pi_{it}^{jx(i,j,t)}$ for $x(i, j, t)$ using individual-specific convex combinations of *K* time-varying probabilities (implicitly introducing, e.g., $\tilde{\pi}_{kt}^{jx(i,j,t)} = \sum_{h=k}^{K} v_{kht} \lambda_h^{jx(i,j,t)}$, where the tilde (~) distinguishes the pure-type probabilities $\tilde{\pi}_{kt}^{jx(i,j,t)}$ from individual-specific probabilities $\pi_{it}^{jx(i,j,t)}$)

with convex coefficients g_{ik} (i.e., $0 \le g_{ik} \le 1$ and $\sum_{k=1}^{K} g_{ik} = 1$). The inner sum in eqn. (S.4) implicitly defines $\mathbf{V}_t = [v_{kht}]$ to be a $K \times K$ upper-triangular transition matrix, with unit row sums, inducing a natural ordering on the AD subtypes from lowest to highest severity, with subtype K representing the endpoint of the process; by convention $\mathbf{V}_0 = \mathbf{I}$, the identity matrix. Procedures for weighted maximum likelihood estimation of the g-, v-, and λ-parameters are described in the Appendix based on the algorithm introduced in [9].

1.2 Mortality and Missing Data

Problems related to mortality and missing data in longitudinal data were resolved using L-GoM's conditional independence property assuming that the missing data were missing at random (MAR) [10], conditional on the non-missing data. The resolution under the MAR assumption was trivially implemented by deleting the associated terms from eqn. (S.4) [9]. If date of death was missing, the final mortality term at T_i in eqn. (S.4) was deleted; if death occurred after a missed scheduled exam at time $T_i + 1$, then an additional mortality/survival term was added for the half-open interval $(T_i + 1, T_i + 2]$. If exams for living individuals were completely missing or if specific covariate items were missing, then the corresponding terms in eqn. (S.4) were deleted under the MAR assumption.

Comment 1—Zehna's theorem [5] proves that the individualized TLEs, DFLEs, and DLEs are MLEs that are statistically optimal under the standard assumption that the associated model, as shown in eqn. (S.4), is correct. Freedman [11] discusses how to proceed when it is known that the associated model is incorrect.

Comment 2—Our prior L-GoM likelihood [12, 13] included both prospective and retrospective coding of survival indicator variables. Zehna's theorem [5] provides theoretical justification for including the prospective survival indicator (using ε_{iT_i}) in the L-GoM likelihood, as shown in eqns. (S.3) and (S.4). In contrast, L-GoM's conditional independence property justifies dropping the retrospective survival indicator (which replicates ε_{iT_i}, but codes the value at time $T_i + 1$) from the current likelihood.

Comment 3—The combination of the MLE mapping from the GoM scores to the TLEs, DFLEs, and DLEs via eqns. (S.1) and (S.2), and their sum, with the MLE mapping from the data to the GoM scores via eqn. (S.4) defines a composite MLE mapping from the data to the TLEs, DFLEs, and DLEs, which can be incorporated directly into important decisions regarding the treatment and care of AD patients. From this perspective, the GoM scores represent intermediate computational phenotypes that are essential to the TLEs, DFLEs, and DLEs.

1.3 Statistical Software

Data preparation and editing were conducted using SAS 9.3. The tables and figures were generated using Microsoft Excel Version 14.0. Weighted MLEs were estimated via eqn. (S.4) using Simply Fortran Version 2.22 to calculate the constrained Newton-Raphson updates [9]; see the Appendix for details.

2. ASSOCIATED IRREVERSIBLE DISABILITY MODEL

2.1 Overview

Although the Sullivan DFLEs and DLEs do not require any assumptions about the underlying disability transition rates, these life expectancies can be usefully decomposed using a unidirectional illness-death model [1, 14] which is specified at the individual level under two assumptions: (1) disability is irreversible; and (2) mortality is conditionally independent of disability status, given the individual's GoM scores. Assumption (1) applies when FTC (or a comparable endpoint covariate) is used to define disability. Assumption (2) is equivalent to L-GoM's conditional independence assumption (see Appendix); it applies when L-GoM fits the data.

The two assumptions are used to specify the associated irreversible disability model depicted in Figure S.1, which shows the states and transitions of the model. Three sets of transition rates, $\mu_i(t)$, $\nu_i(t)$, and $\upsilon_i(t)$, govern nondisabled mortality, disability onset, and disabled mortality, respectively; they operate in continuous time as individual-specific functions of *t*. Assumption (1) restricts the directions of the transitions; Assumption (2) restricts the mortality rates to be equal for all *t*, i.e., $\mu_i(t) \equiv \upsilon_i(t)$. These assumptions are sufficient to generate the conditional DFLEs and DLEs, given the GoM scores for individual *i*, as shown in the Appendix. The model yields clinically interpretable conditional mortality and disability probabilities, and associated conditional timing measures, for individual AD patients. Section 2.5 discusses how these assumptions might be relaxed.

2.2 Conditional Probabilities of Disability and Death

Table S.1 displays the means, standard errors, and ranges of the 10-year mortality and disability probabilities for all participants and for subgroups 0–4 under the associated irreversible disability model, assuming that all survivors at the end of year 10 (Exam 21) died immediately thereafter—consistent with the 10-year censoring assumption in Table 1. Column 2 contains the average probabilities of being free of FTC at study intake among individuals in the indicated subgroups. Column 3 contains the average joint probabilities of being free of FTC at study intake and dying without ever transitioning to FTC. Column 4 contains the average joint probabilities of being free of FTC at study intake and transitioning to FTC prior to death. Column 6 contains the average probabilities of FTC at any point prior to death. Column 7 contains the average probabilities of FTC at study intake while column 8 contains the average probabilities of transitioning to FTC after study intake but prior to death. The probabilities in columns 4 and 8 represent the same two-stage

outcome: No FTC at intake followed by FTC followed by death. Column 5 repeats column 4 for later alignment with Table S.2.

Overall, 91.8% of the sample was free of FTC at intake and 55.4% experienced FTC at some time during the 10-year follow-up. Subgroup 4 was distinguished by its relatively high average probability of FTC (68.4%), most of which was present at study intake (54.3%). This contrasted with subgroup 2 which also had a relatively high average probability of FTC (62.7%), but most FTC occurred after study intake (56.8%).

2.3 Conditional DFLEs and DLEs

Table S.2 displays the corresponding means, standard errors, and ranges of the 10-year conditional DFLEs, conditional DLEs, and related conditional times to death and FTC for all participants and for subgroups 0–4. *Conditional DFLE* (col. 2) is the average time lived between (1) study intake and (2) death, onset of disability, or time ω (i.e., 10 years), whichever occurred first, given that disability occurred after study intake. Among those with no FTC at study intake (col. 2), death and FTC represent competing risks [1]: *Time to Death* (col. 3) represents the case that death occurred prior to FTC and *Time to FTC* (col. 4) the case that FTC occurred prior to death. *Conditional DLE* (col. 6) is the average time lived between (1) the onset of disability and (2) death or time ω, whichever occurred first, given that disability occurred prior to time ω. The *Conditional DLEs* were further restricted to include (*Conditional DLE_1*; col. 7) or exclude (*Conditional DLE_2*; col. 8) disability present at study intake. *Total Time to Death* (col. 5) is the sum of *Time to FTC* (col. 4) and *Conditional DLE_2* (col. 8).

The ratios in Table S.2 (bottom) show that the largest differences between subgroups occurred for *Conditional DFLE* and its components (2.41–2.56 yrs.; cols. 2–4); these ratios were less than half of the corresponding *DFLE* ratio in Table 1 (5.24 yrs.; col. 3) because of the conditioning on intake status (i.e., *DFLE*=1.23 yrs.; *Cond. DFLE*=2.70 yrs.). Subgroup 2 was distinguished by having the shortest *Time to FTC* among those with no FTC at study intake (2.19 yrs.; col. 4), with subgroup 1 having the longest time (5.37 yrs.). Conversely, *Conditional DLE_2* (col. 8) exhibited relatively little variation over subgroup.

2.4 Decomposing the Sullivan Life Expectancies

The irreversible disability model was designed to uniquely decompose the Sullivan life expectancies. Conversely, the Sullivan life expectancies can be regenerated from the associated irreversible disability model. Specifically, the *DFLEs* and *DLEs* in Table 1 (cols. 3 and 4) can be decomposed into products of the *Conditional DFLEs* and *Conditional DLEs* in Table S.2 (cols. 2 and 6) with the corresponding probabilities in Table S.1. Given that the *TLEs* in Table 1 are the sums of the corresponding *DFLEs* and *DLEs*, it also follows that the *TLEs* are probability-weighted sums of the corresponding *Conditional DFLEs* and *Conditional DLEs* in Table S.2. Alternatively, the *TLEs* in Table 1 can be decomposed as probability-weighted averages of *Time to Death*, *Total*

Time to Death, and *Conditional DLE* in Table S.2 (cols. 3, 5, and 7), using the corresponding probabilities in Table S.1.

Using the above relationships, the average Sullivan *DFLE* of 4.03 can be decomposed into the product of a 91.8% average probability of no FTC at intake and the average *Conditional DFLE* of 4.39 years; the average Sullivan *DLE* of 2.06 can be decomposed into the product of a 55.4% average probability of FTC and the average *Conditional DLE* of 3.72 years; and the average *TLE* of 6.09 years can be decomposed into the probability-weighted average of 5.49, 6.95, and 4.46 years for *Time to Death* (Table S.2; col. 3), *Total Time to Death* (Table S.2; col. 5) and *Conditional DLE_1* (Table S.2; col. 7), with probabilities 44.6%, 47.2%, and 8.2%, respectively.

Time to FTC (Table S.2; col. 4) and *Conditional DLE_2* (Table S.2; col. 8) sum to yield *Total Time to Death* (Table S.2; col. 5), although neither is represented in Table 1. The associated irreversible disability model extracts them from the SLT/L-GoM model—important because *Time to FTC* among patients free of FTC at intake is a major endpoint in AD research [15-17].

2.5 Discussion

The Sullivan [2] life table calculations were designed to generate estimates of DFLE, DLE, and the associated survival functions, without having to specify an underlying transition model or to restrict the analytic sample to AD patients free of disability at the intake examination. The SLT/L-GoM extension allows these same estimates to be generated on an individual-specific basis. Moreover, because the L-GoM extension uses all relevant covariates from the longitudinal panel data for all AD patients meeting the intake criteria, it represents a highly efficient mode of analysis.

The associated irreversible disability model provides a unique and simple decomposition of the Sullivan life expectancies and it clarifies the relationship of the Sullivan life expectancies to other life-expectancy calculations that might be obtained from application of multi-state competing-risk models [1]. The assumptions of no recovery and conditional independence may be oversimplifications, but any resulting biases must be compensating because the Sullivan estimates of the DFLEs and DLEs are unique. Hence, the associated irreversible disability model provides a readily-generated baseline for evaluation of more complex disability models.

The conditional independence assumption is not required for the standard form of the Sullivan [2] model. It is required for the L-GoM application. For sufficiently large J with $K \geq 4$, the assumption is expected to be valid within the set of J covariates measured at each examination. The quality of the assumption for each longitudinal sequence of morbidity/disability covariate values should be validated. In the case of FTC, our validation was based on the goodness of fit of the estimated vs. observed values within the five rational subgroups shown in Figure 3.

If the conditional independence assumption cannot be validated for a specific disability covariate, one can estimate separate L-GoM models for the disability states shown in Figure S.1,

stratifying the intake population according to their initial disability status. In the case of FTC, the specification for the disabled subpopulation would look like the current specification except that FTC would be dropped as an internal variable. The specification for the nondisabled subpopulation be would similar except that the mortality/survival covariate would include transition to FTC as a competing risk for termination of the disability-free process [4]. The separate L-GoM models could be linked by joint (i.e., constrained) estimation of the individual GoM scores or by constraining the separately estimated GoM scores to be correlated for individuals observed in both components of the stratified analysis [18].

Finally, we emphasize that the irreversible disability assumption is not required for the standard form of the Sullivan [2] model; nor is it required for the L-GoM application. This means that the SLT/L-GoM model can be used to generate individual-specific DFLE and DLE estimates for transient forms of morbidity/disability such as behavioral and psychiatric symptoms, depression, and other signs/symptoms differentially associated with the non-terminal prognostic subtype 3 [19].

REFERENCES

1. Andersen PK, Keiding N: **Multi-state models for event history analysis.** *Statistical Methods in Medical Research* 2002, **11:**91-115.
2. Sullivan DF: **A single index of mortality and morbidity.** *HSMHA Health Reports* 1971, **86:**347-354.
3. Irwin J: **The standard error of an estimate of expectation of life, with special reference to expectation of tumourless life in experiments with mice.** *Journal of Hygiene* 1949, **47:**188-189.
4. Katz S, Branch LG, Branson MH, Papsidero JA, Beck JC, Greer DS: **Active life expectancy.** *New England Journal of Medicine* 1983, **309:**1218-1224.
5. Zehna PW: **Invariance of maximum likelihood estimators.** *Annals of Mathematical Statistics* 1966, **37:**744.
6. Wieland D, Kinosian B, Stallard E, Boland R: **Does Medicaid pay more to a Program of All-inclusive Care for the Elderly (PACE) than for fee-for-service long-term care?** *Journal of Gerontology: Medical Sciences* 2013, **68:**47-55.
7. Maetzel A, Johnson SH, Woodbury M, Bombardier C: **Use of Grade of Membership analysis to profile the practice styles of individual physicians in the management of acute low back pain.** *Journal of Clinical Epidemiology* 2000, **53:**195-205.
8. Corder LS, Woodbury MA, Manton KG: **Proxy response patterns among the aged: Effects on estimates of health status and medical care utilization from the 1982–1984 Long-Term Care Surveys.** *Journal of Clinical Epidemiology* 1996, **49:**173-182.
9. Stallard E, Sloan FA: **Analysis of the natural history of dementia using longitudinal Grade of Membership models.** In *Biodemography of Aging: Determinants of Healthy Life Span and Longevity.* Edited by Yashin AI, Stallard E, Land KC. Dordrecht: Springer Netherlands; 2016: 353-418
10. Rubin DB: **Inference and missing data.** *Biometrika* 1976, **63:**581-592.
11. Freedman DA: **On the so-called "Huber sandwich estimator" and "robust standard errors".** *American Statistician* 2006, **60:**299-302.
12. Stallard E: **Trajectories of morbidity, disability, and mortality among the U.S. elderly population: Evidence from the 1984-1999 NLTCS.** *North American Actuarial Journal* 2007, **11:**16-53.
13. Stallard E, Kinosian B, Zbrozek AS, Yashin AI, Glick HA, Stern Y: **Estimation and validation of a multiattribute model of Alzheimer disease progression.** *Medical Decision Making* 2010, **30:**625-638.
14. Touraine C, Helmer C, Joly P: **Predictions in an illness-death model.** *Statistical Methods in Medical Research* 2016, **25:**1452-1470.
15. Stern Y, Tang M-X, Albert MS, Brandt J, Jacobs DM, Bell K, Marder K, Sano M, Devanand D, Albert SM, et al: **Predicting time to nursing home care and death in individuals with Alzheimer disease.** *JAMA* 1997, **277:**806-812.
16. Caro JJ, Getsios D, Migliaccio–Walle K, Raggio G, Ward A, Group AS: **Assessment of health economics in Alzheimer's disease (AHEAD) based on need for full-time care.** *Neurology* 2001, **57:**964-971.
17. Rive B, Le Reun C, Grishchenko M, Cochran J, Katona C, Livingston G, Lamure M, Toumi M, François C: **Predicting time to full-time care in AD: A new model.** *Journal of Medical Economics* 2010, **13:**362-370.

18. Manton KG, Woodbury MA, Stallard E, Corder LS: **The use of Grade-of-Membership techniques to estimate regression relationships.** *Sociological Methodology* 1992**:**321-381.
19. Scarmeas N, Brandt J, Albert M, Hadjigeorgiou G, Papadimitriou A, Dubois B, Sarazin M, Devanand D, Honig L, Marder K: **Delusions and hallucinations are associated with worse outcome in Alzheimer disease.** *Archives of Neurology* 2005, **62:**1601-1608.
20. Imai K, Soneji S: **On the estimation of disability-free life expectancy: Sullivan's method and its extension.** *Journal of the American Statistical Association* 2007, **102:**1199-1211.
21. Kaplan EL, Meier P: **Nonparametric estimation from incomplete observations.** *Journal of the American Statistical Association* 1958, **53:**457-481.
22. Razlighi QR, Stallard E, Brandt J, Blacker D, Albert M, Scarmeas N, Kinosian B, Yashin AI, Stern Y: **A new algorithm for predicting time to disease endpoints in Alzheimer's disease patients.** *Journal of Alzheimer's Disease* 2014, **38:**661-668.
23. Suppes P, Zanotti M: **When are probabilistic explanations possible?** *Synthese* 1981, **48:**191-199.
24. Suppes P: **Probabilistic causality in space and time.** In *Causation, Chance, and Credence. Volume* 1. Edited by Skyrms B, Harper WL. Dordrecht: Kluwer Academic Publishers; 1988: 135-151
25. Woodbury MA, Manton KG, Tolley HD: **A general model for statistical analysis using fuzzy sets: sufficient conditions for identifiability and statistical properties.** *Information Sciences-Applications* 1994, **1:**149-180.
26. Woodbury MA, Clive J: **Clinical pure types as a fuzzy partition.** *Journal of Cybernetics* 1974, **4:**111-121.
27. MacKinnon DP, Lockwood CM, Hoffman JM, West SG, Sheets V: **A comparison of methods to test mediation and other intervening variable effects.** *Psychological Methods* 2002, **7:**83-104.
28. Kinosian B, Stallard E, Manton KG, Straley DL, Zbrozek AS, Glick HA: **The expected outcomes and costs of US patients with incident suspected Alzheimer's Disease (AD) over 15 years.** In *Abstract of Poster Session at the 9th International Conference on Alzheimer's Disease and Related Disorders, Philadelphia, PA, July*. 2004: 17-22.
29. Wilks SS: **The large-sample distribution of the likelihood ratio for testing composite hypotheses.** *Annals of Mathematical Statistics* 1938, **9:**60-62.
30. Fillenbaum GG, Woodbury MA: **Typology of Alzheimer's disease: Findings from CERAD data.** *Aging & Mental Health* 1998, **2:**105-127.
31. Hu F, Rosenberger WF, Zidek JV: **Relevance weighted likelihood for dependent data.** *Metrika* 2000, **51:**223-243.
32. Hu F, Zidek JV: **The weighted likelihood.** *Canadian Journal of Statistics* 2002, **30:**347-371.
33. Akaike H: **Information theory and an extension of the maximum likelihood principle.** In *Proceedings of the Second International Symposium on Information Theory.* Edited by Petrov BN, Csaki F. Budapest, Hungary: Akademiai Kaido; 1973: 267-281
34. Boos DD, Stefanski LA: *Essential Statistical Inference: Theory and Methods.* New York: Springer; 2013.

35. Airoldi EM, Blei DM, Erosheva EA, Fienberg SE (Eds.): **Handbook of Mixed Membership Models and Their Applications**. Boca Raton, FL: Chapman and Hall/CRC; 2015.
36. Wang X: **Approximating Bayesian inference by weighted likelihood.** *Canadian Journal of Statistics* 2006, **34:**279-298.
37. Manrique-Vallier D: **Longitudinal mixed membership trajectory models for disability survey data.** *Annals of Applied Statistics* 2014, **8:**2268-2291.
38. Manrique-Vallier D: **Mixed Membership Trajectory Models.** In *Handbook of Mixed Membership Models and Their Application.* Edited by Airoldi EM, Blei DM, Erosheva EA, Fienberg SE. Boca Raton, FL: Chapman and Hall/CRC; 2015
39. Kullback S, Leibler RA: **On information and sufficiency.** *Annals of Mathematical Statistics* 1951, **22:**79-86.
40. Rao CR: **Maximum likelihood estimation for the multinomial distribution with infinite number of cells.** *Sankhyā: The Indian Journal of Statistics* 1958**:**211-218.
41. Sefik M: **Importance of the rational subgroups in designing control charts.** *Computers & Industrial Engineering* 1998, **35:**205-208.
42. Nair VN: **Confidence bands for survival functions with censored data: a comparative study.** *Technometrics* 1984, **26:**265-275.
43. Agresti A, Coull BA: **Approximate is better than "exact" for interval estimation of binomial proportions.** *American Statistician* 1998, **52:**119-126.
44. Potthoff RF, Woodbury MA, Manton KG: **"Equivalent sample size" and "equivalent degrees of freedom" refinements for inference using survey weights under superpopulation models.** *Journal of the American Statistical Association* 1992, **87:**383-396.
45. McKhann G, Drachman D, Folstein M, Katzman R, Price D, Stadlan EM: **Clinical diagnosis of Alzheimer's disease: Report of the NINCDS-ADRDA Work Group under the auspices of Department of Health and Human Services Task Force on Alzheimer's Disease.** *Neurology* 1984, **34:**939-944.

Table S.1. Means, Standard Errors, and Ranges of Probabilities of Disability and Death over 10 Years by Subgroup, based on Need for Full-Time Care (FTC) under the Associated Irreversible Disability Model

	N	No FTC at Intake				FTC at Any Time		
		Total	Death before FTC	FTC before Death	Death after FTC	Total	FTC at Intake	No FTC at Intake; FTC before Death
	(1)	(2)	(3)	(4)	(5)	(6)	(7)	(8)
				**Mean Probability (%)**				
Subgroup								
1	59	99.4	56.8	42.6	42.6	43.2	0.6	42.6
2	87	94.1	37.3	56.8	56.8	62.7	5.9	56.8
3	29	94.6	44.8	49.8	49.8	55.2	5.4	49.8
4	11	45.7	31.6	14.0	14.0	68.4	54.3	14.0
0	43	86.4	45.9	40.5	40.5	54.1	13.6	40.5
Total	229	91.8	44.6	47.2	47.2	55.4	8.2	47.2
				**Standard Error of Mean Probability (%)**				
Subgroup								
1	59	0.3	0.4	0.5	0.5	0.4	0.3	0.5
2	87	0.9	0.6	1.0	1.0	0.6	0.9	1.0
3	29	1.8	0.5	2.0	2.0	0.5	1.8	2.0
4	11	2.5	1.7	1.6	1.6	1.7	2.5	1.6
0	43	1.9	0.7	1.6	1.6	0.7	1.9	1.6
Total	229	0.9	0.6	0.9	0.9	0.6	0.9	0.9
Maximum	229	99.4	56.8	56.8	56.8	68.4	54.3	56.8
Minimum	229	45.7	31.6	14.0	14.0	43.2	0.6	14.0
Ratio	229	2.2	1.8	4.1	4.1	1.6	84.8	4.1

Notes: Survival beyond 10 years was censored; all survivors at year 10 were assumed to die immediately thereafter. *FTC before Death* (col. 4), *Death after FTC* (col. 5), and *No FTC at Intake; FTC before Death* (col. 8) are alternative representations of the same outcome. The probabilities in cols. 2 and 7 sum to 100%; the probabilities in cols. 3 and 4 sum to the probabilities in col. 2; and the probabilities in cols. 7 and 8 sum to the probabilities in col. 6. The standard errors reflect the variation between individuals of the indicated estimates; other sources of variation were assumed negligible. Minima and maxima are for subgroups.

Table S.2. Means, Standard Errors, and Ranges of 10-Year Conditional Life Expectancies by Subgroup, based on Need for Full-Time Care (FTC) under the Associated Irreversible Disability Model

	N	No FTC at Intake				FTC at Any Time		
		Total	Death before FTC	FTC before Death	Death after FTC	Total	FTC at Intake	No FTC at Intake; FTC before Death
Type of Estimate		*Cond. DFLE*	*Time to Death*	*Time to FTC*	*Total Time to Death*	*Cond. DLE*	*Cond. DLE_1*	*Cond. DLE_2*
	(1)	(2)	(3)	(4)	(5)	(6)	(7)	(8)
					**Mean (Years)**			
Subgroup								
1	59	6.51	7.37	5.37	8.81	3.49	7.12	3.44
2	87	3.03	4.29	2.19	5.83	3.72	4.59	3.63
3	29	4.78	5.56	4.09	7.67	3.77	5.44	3.59
4	11	2.70	2.87	2.32	5.31	3.42	3.53	3.00
0	43	4.01	4.70	3.24	6.99	4.03	4.86	3.75
Total	229	4.39	5.49	3.36	6.95	3.72	4.46	3.59
					**Standard Error of Mean (Years)**			
Subgroup								
1	59	0.13	0.12	0.14	0.08	0.07	0.18	0.07
2	87	0.05	0.07	0.04	0.06	0.02	0.08	0.03
3	29	0.17	0.21	0.16	0.13	0.05	0.24	0.04
4	11	0.13	0.08	0.26	0.26	0.08	0.10	0.04
0	43	0.12	0.15	0.10	0.12	0.03	0.13	0.04
Total	229	0.11	0.11	0.10	0.09	0.02	0.12	0.02
Maximum	229	6.51	7.37	5.37	8.81	4.03	7.12	3.75
Minimum	229	2.70	2.87	2.19	5.31	3.42	3.53	3.00
Ratio	229	2.41	2.56	2.45	1.66	1.18	2.02	1.25

Notes: All conditional life expectancy (LE) estimates are 10-year LEs. Survival beyond 10 years was censored; all survivors at year 10 were assumed to die immediately thereafter. Mean values refer to the probability-weighted expected values of conditional LE estimates for individuals, weighted with the corresponding probabilities defined in Table S.1. *Cond. DFLE* (col. 2) is a probability-weighted average of *Time to Death* (col. 3) and *Time to FTC (col. 4)*. *Cond. DLE* (col. 6) is a probability-weighted average of *Cond. DLE_1* (col. 7) and *Cond. DLE_2* (col. 8). *Total Time to Death* (col. 5) is the sum of *Time to FTC* (col. 4) and *Cond. DLE_2* (col. 8). The standard errors reflect the variation between individuals of the indicated estimates; other sources of variation were assumed negligible. Section 2.7 of the Supplementary Appendix describes the calculations of the standard errors. Minima and maxima are for subgroups.

Figure S.1: States and transitions for the associated irreversible disability model for individual AD patients, with disability defined as the need for full-time care (FTC), estimated under the SLT/L-GoM model with $\mu_i(t) \equiv \upsilon_i(t)$.

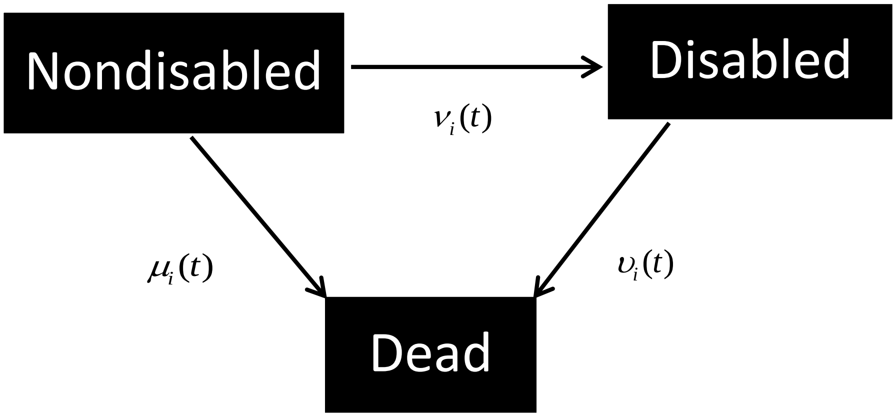

APPENDIX

This Appendix has two sections. Section 1 presents the technical details of the L-GoM extension of the Sullivan life table model including treatment of genetic and other unchanging fixed covariates such as sex and ApoE status; this section also describes the weighted maximum likelihood estimation procedure and presents the estimated parameters and related test statistics. Section 2 characterizes the relationship of the DFLEs and DLEs obtained from the Sullivan life table model to the corresponding conditional DFLEs and DLEs obtained from the associated irreversible disability model.

1. L-GoM EXTENSION OF SULLIVAN MODEL

This section provides the technical details on the L-GoM extension of the Sullivan life table (SLT) model (SLT/L-GoM). Before starting, we note that independent use of eqn. (S.3) for each individual *i* would yield trivial maximum likelihood estimates: $\hat{p}_{it} = 1$, $\hat{p}_{iT_i} = \varepsilon_{iT_i}$, and $\hat{\pi}_{it} = \delta_{it}$; these would be of little practical use.

Thus, to generate nontrivial ML estimates, the observations must be pooled over individuals in the study population. Two methods are considered herein; both involve products over individuals of the likelihood contributions in eqn. (S.3).

The *first* method assumes that the parameters $p_{it} \equiv p_t$ and $\pi_{it} \equiv \pi_t$ are constant over individuals; this is the defining assumption for Sullivan's cohort model [2, 20]. ML estimates for the survival parameters $p_{it} \equiv p_t$ were generated using Kaplan and Meier's product-limit method [21]; ML estimates for the disability parameters $\pi_{it} \equiv \pi_t$ were generated using the observed prevalence rates at time *t*. Imai and Soneji [20] proved that Sullivan's cohort estimator is both consistent and unbiased while Zehna's theorem [5] implies that it is an MLE. The *observed* survival and disability rates, and their products, were calculated as indicated above for each time *t* for all participants and for select subgroups of AD patients in Predictors 2 (see Figures 2–4). The observed survival and disability rates were needed to validate the corresponding model-based estimates produced by the SLT/L-GoM model.

The *second* method assumes that the parameters p_{it} and π_{it} vary over individuals, which, with additional assumptions described below, yields the SLT/L-GoM model. This initial assumption was implemented using two sets of "hidden" parameters to generate p_{it} and π_{it}—with one set (denoted $\tilde{p}_{kt}$ and $\tilde{\pi}_{kt}$) shared over individuals and the other (denoted g_{ik}) not shared over individuals, where the subscripts *k*, *k* = 1,…, *K*, index the AD subtypes within the L-GoM model.

The hidden parameters are assumed to satisfy two fundamental bilinear relations: $p_{it} = \sum_{k=1}^{K} g_{ik}\tilde{p}_{kt}$ and $\pi_{it} = \sum_{k=1}^{K} g_{ik}\tilde{\pi}_{kt}$, which are the defining assumptions for L-GoM.

To apply the second method, we had to extend the scope of eqn. (S.3) beyond a single selected morbidity/disability variable to include other relevant covariates as internal variables. The extension was justified because L-GoM provides a realistic and comprehensive model of the AD process, as demonstrated in the current and prior papers [13, 22]. Moreover, some extension was required for identifiability (see below); once identifiability is achieved, the accuracy of the estimates of the hidden parameters improves as additional covariates are introduced (Stallard and Sloan, 2016).

We require new notation both to extend the scope of eqn. (S.3) to include $J > 1$ internal covariates and to bridge from the Sullivan life table notation to the standard longitudinal GoM notation:

1. Define $X(i, 1, t)$ as the dichotomous morbidity/disability covariate, with outcomes denoted by $x(i, 1, t)$, and with corresponding conditional outcome probabilities denoted by $\pi_{it}^{1x(i,1,t)}$, where $\pi_{it}^{1x(i,1,t)}$ denotes either π_{it} or π_{it}^{c} depending on $x(i, 1, t)$.
   - Define $\tilde{\pi}_{kt}^{1x(i,1,t)}$ as the corresponding hidden conditional outcome probability for subtype k, where $\tilde{\pi}_{kt}^{1x(i,1,t)}$ denotes either $\tilde{\pi}_{kt}$ or $\tilde{\pi}_{kt}^{c}$ depending on $x(i, 1, t)$.
2. Define $X(i, j, t)$, $j = 2,\ldots, J$, as the jth polychotomous internal variable for individual i at time t, taking a finite number of values, denoted by $x(i, j, t)$, with corresponding conditional outcome probabilities denoted by $\pi_{it}^{jx(i,j,t)}$.
   - Define $\tilde{\pi}_{kt}^{jx(i,j,t)}$ as the corresponding hidden conditional outcome probability for subtype k.

   For consistency, we extend the assumption that $\pi_{it}^{jx(i,j,t)}$ satisfies the fundamental bilinear relation $\pi_{it}^{jx(i,j,t)} = \sum_{k=1}^{K} g_{ik}\tilde{\pi}_{kt}^{jx(i,j,t)}$ for all j, $j = 1,\ldots, J$.

3. Define $X(i, 0, t)$ as the dichotomous mortality/survival covariate, with outcomes denoted by $x(i, 0, t)$, and with corresponding conditional outcome probabilities denoted by $\pi_{it}^{0x(i,0,t)} \equiv p_{it}^{x(i,0,t)}$ where $p_{it}^{x(i,0,t)}$ denotes either p_{it}, p_{iT_i}, or $p_{iT_i}^{c}$ depending on $x(i, 0, t)$.
   - Define $\tilde{\pi}_{kt}^{0x(i,0,t)} \equiv \tilde{p}_{kt}^{x(i,0,t)}$ as the corresponding hidden conditional outcome probability for subtype k, where $\tilde{p}_{kt}^{x(i,0,t)}$ denotes either $\tilde{p}_{kt}$, $\tilde{p}_{kT_i}$, or $\tilde{p}_{kT_i}^{c}$ depending on $x(i, 0, t)$.

By reserving the index $j = 0$ for the prospective mortality/survival indicator, we highlight the special role of this variable within the extended Sullivan life table model. It must always be included within the set of internal variables in the SLT/L-GoM model. In contrast, specific morbidity/disability covariates indexed by $j = 1,\ldots, J$ may or may not be included in different versions of the SLT/L-GoM model.

Under these conditions, we can extend the scope of eqn. (S.3) as follows:

$$L_i = \prod_{t=0}^{T_i-1}\left(p_{it}\right)\times\left(p_{iT_i}\right)^{\varepsilon_{iT_i}}\left(p_{iT_i}^{c}\right)^{1-\varepsilon_{iT_i}}\times\prod_{t=0}^{T_i}\left(\pi_{it}^{1x(i,1,t)}\right)\times\prod_{t=0}^{T_i}\left(\prod_{j=2}^{J}\left(\pi_{it}^{jx(i,j,t)}\right)\right) \quad \text{(A.1a)}$$

$$= \prod_{t=0}^{T_i-1}\left(\sum_{k=1}^{K} g_{ik}\tilde{p}_{kt}\right)\times\left(\sum_{k=1}^{K} g_{ik}\tilde{p}_{kT_i}\right)^{\varepsilon_{iT_i}}\left(\sum_{k=1}^{K} g_{ik}\tilde{p}_{kT_i}^{c}\right)^{1-\varepsilon_{iT_i}}\times\prod_{t=0}^{T_i}\prod_{j=1}^{J}\left(\sum_{k=1}^{K} g_{ik}\tilde{\pi}_{kt}^{jx(i,j,t)}\right) \quad \text{(A.1b)}$$

$$= \prod_{t=0}^{T_i}\prod_{j=0}^{J}\left(\sum_{k=1}^{K} g_{ik}\tilde{\pi}_{kt}^{jx(i,j,t)}\right) \quad \text{(A.1c)}$$

$$= \prod_{t=0}^{T_i}\prod_{j=0}^{J}\left(\pi_{it}^{jx(i,j,t)}\right), \quad \text{(A.1d)}$$

where $\tilde{p}_{kt}$, $\tilde{p}_{kT_i}$, $\tilde{p}_{kT_i}^{c}$, $\tilde{\pi}_{kt}^{jx(i,j,t)}$, and g_{ik} are restricted to the range 0–1. Eqn. (A.1a) introduces covariates $j = 2,\ldots, J$ into eqn. (S.3); the original factors for $j = 1$ were rewritten to reflect the new notation introduced above. To move from eqn. (A.1a) to (A.1b), we used the fundamental bilinear relations: $p_{it} = \sum_{k=1}^{K} g_{ik}\tilde{p}_{kt}$, $\pi_{it} = \sum_{k=1}^{K} g_{ik}\tilde{\pi}_{kt}$, and $\pi_{it}^{jx(i,j,t)} = \sum_{k=1}^{K} g_{ik}\tilde{\pi}_{kt}^{jx(i,j,t)}$, and we combined the factors for covariates $j = 1,\ldots, J$. Each sum in eqn. (A.1b) represents a probability in the range 0–1 generated by an individual-specific convex combination of the basic (i.e., shared) probabilities $\tilde{p}_{kt}$ and $\tilde{\pi}_{kt}^{jx(i,j,t)}$ with convex coefficients $\{g_{ik}\}$, the set of K GoM scores for individual i, which are constrained to sum over k to 1. To move from eqn. (A.1b) to (A.1c), we substituted $\tilde{\pi}_{kt}^{0x(i,0,t)}$ for $\tilde{p}_{kt}$, $\tilde{p}_{kT_i}$, and $\tilde{p}_{kT_i}^{c}$, and we combined the factors for covariates $j = 0,\ldots, J$. To move from eqn. (A.1c) to (A.1d), we substituted $\pi_{it}^{jx(i,j,t)}$ for $\sum_{k=1}^{K} g_{ik}\tilde{\pi}_{kt}^{jx(i,j,t)}$ using the general form of the assumed bilinear relation for $j = 0,\ldots, J$.

It follows from the product form of the rightmost factor in eqn. (A.1b), i.e., $\prod_{t=0}^{T_i}\prod_{j=1}^{J}\left(\sum_{k=1}^{K} g_{ik}\tilde{\pi}_{kt}^{jx(i,j,t)}\right)$, that the $J\times(T_i+1)$ morbidity/disability covariates are conditionally independent, given the GoM scores, which is sufficient to characterize L-GoM with $\pi_{it}^{jx(i,j,t)} = \sum_{k=1}^{K} g_{ik}\tilde{\pi}_{kt}^{jx(i,j,t)}$ as a latent-variable model and the GoM score vector $\mathbf{g}_i = [g_{ik}]$, $k = 1,\ldots, K$,

as the "common cause" of the observed outcomes [23, 24]. Equivalently, one could assume that the $J \times (T_i + 1)$ morbidity/disability covariates are conditionally independent, from which it follows that the rightmost factor has the product form shown in eqn. (A.1b). Because the rightmost factor in eqn. (A.1b) is the conditional probability of the indicated composite morbidity/disability outcome, the remaining multiplications in eqn. (A.1b) constitute an application of the chain rule for products of conditional probabilities [21].

The final assumption needed for the L-GoM model is that the temporal progression of AD is governed solely by a finite set of $K \times K$ upper triangular cumulative transition matrices $\mathbf{V}_t = [v_{kht}]$, $t = 0, \ldots, \omega$, covering the time intervals from 0 to ω, that irreversibly change the probabilities faced by individuals at each time t from those of lower-numbered subtypes to higher-numbered subtypes. By convention, $\mathbf{V}_0 = \mathbf{I}$, an identity matrix, and $\mathbf{V}_{t+1} = \mathbf{V}_t \mathbf{U}_t$, where $\mathbf{U}_t = [u_{kht}]$ is a $K \times K$ upper triangular transition matrix associated with the unit time interval (t, t + 1). The $\mathbf{V}_t$'s govern the cumulative changes in the probabilities over time which induce a natural ordering on the subtypes with the Kth subtype representing the endpoint of the AD process. Because the $\mathbf{V}_t$'s contain the shared u-parameters, they fully subsume the temporal variability of the $\tilde{\pi}_{kt}^{jx(i,j,t)}$'s and this dramatically reduces the number of parameters needed to fit the model to the study data. Hence, eqn. (A.1c) can be rewritten as:

$$L_i = \prod_{t=0}^{T_i} \prod_{j=0}^{J} \left(\sum_{k=1}^{K} \sum_{h=k}^{K} g_{ik} v_{kht} \lambda_h^{jx(i,j,t)} \right), \tag{A.2}$$

where $\tilde{\pi}_{kt}^{jx(i,j,t)}$ is replaced with $\sum_{h=k}^{K} v_{kht} \lambda_h^{jx(i,j,t)}$; where the $\lambda_k^{jx(i,j,t)}$'s constitute a second set of shared parameters. MLEs of the g-, v-, u-, and λ-parameters can be obtained using eqn. (A.2), as detailed in [9] and as modified in Section 2. The large sample statistical properties of L-GoM [9] apply directly to the Sullivan extension.

The GoM model is generally identifiable if the number of internal variables J (i.e., excluding the mortality/survival indicator) is at least twice the number of disease subtypes K [25]. We used $J = 79$ with $K = 4$ in the current L-GoM analysis. The average number of responses per participant, i.e., $J \times (T_i + 1)$, was 520, which was far in excess of that needed for identifiability. The value of K must be sufficiently large that the conditional independence assumption holds, in which case the GoM scores constitute a set of underlying (latent) causal variables [23, 24]. $K = 4$ was found empirically to be the smallest K value that meets this condition for AD (see Section 2 and [13]).

1.1 Comment on g-λ Duality

The representation of the temporal progression of AD via eqn. (A.2) implies a duality wherein the temporal changes may be associated with either the g- or λ-parameters. Under the treatment above, the conditional probability that $X(i, j, t) = x(i, j, t) = l$, given the initial vector $\mathbf{g}_i$, is:

$$\Pr[X(i,j,t)=l \mid \mathbf{g}_i] = \sum_{k=1}^{K} g_{ik}\tilde{\pi}_{kt}^{jl}, \tag{A.3}$$

where $\tilde{\pi}_{kt}^{jl} \equiv \sum_{h=k}^{K} v_{kht}\lambda_h^{jl}$. Thus, the temporal changes in $\tilde{\pi}_{kt}^{jl}$ reflect those captured by the matrix $\mathbf{V}_t$.

An equivalent alternative treatment introduces a set of time-varying GoM scores [13], defined as $\boldsymbol{\gamma}_{it} = \mathbf{V}_t^T \mathbf{g}_i$ (where the superscript T denotes matrix transposition), which leads to the following expression:

$$\Pr[X(i,j,t)=l \mid \boldsymbol{\gamma}_{it}] = \sum_{k=1}^{K} \gamma_{itk}\lambda_k^{jl}, \tag{A.4}$$

where γ_{itk} is the kth element of $\boldsymbol{\gamma}_{it}$. Under this treatment, the temporal progression of AD is governed solely by the cumulative transition matrices, $\mathbf{V}_t$, which irreversibly move individuals (vs. probabilities in eqn. A.3) from lower-numbered to higher-numbered subtypes at rates determined by the vector $\mathbf{g}_i$.

Except for the temporal indexes on $\tilde{\pi}_{kt}^{jl}$ and γ_{itk}, eqns. (A.3) and (A.4) have precisely the same form as the cross-sectional GoM model ([26]; eqn. 3.2). The parametrization in eqn. (A.3) emphasizes the role of the initial vector $\mathbf{g}_i$ as the fundamental summarization of the measured covariates $X(i, j, t)$ for individual i; the hidden outcome probability structure is represented as changing over time. The parametrization in eqn. (A.3) also implies that the g_{ik}'s are implicit functions of the measured covariates $X(i, j, t)$, making L-GoM substantially more flexible than alternative models that rely on explicit functional forms (e.g., logistic regression) to meet the 0–1 boundary constraints on estimated probabilities. The parametrization in eqn. (A.4) emphasizes the role of the temporal changes in $\boldsymbol{\gamma}_{it}$ as the primary drivers of AD progression; the outcome probability structure is represented as constant over time.

1.2 Fixed Covariates

Six fixed covariates required special treatment to satisfy L-GoM's conditional independence assumption. They were included in the likelihood only for Exam 1; the corresponding terms for subsequent exams can be deleted by rewriting eqn. (A.2) as:

$$L_i = \prod_{j=0}^{J} \prod_{t=0}^{\Theta_{ij}} \left(\sum_{k=1}^{K} \sum_{h=k}^{K} g_{ik} v_{kht} \lambda_h^{jx(i,j,t)} \right), \tag{A.5}$$

where $\Theta_{ij} = T_i$ if covariate *j* is time-varying and $\Theta_{ij} = 0$ if *j* is fixed. The total likelihood is the product over all individuals in the study of terms of the form (A.5)—shown in eqn. (S.4). The conditional independence property allows the estimated GoM scores to be interpretable as mediators or intervening variables between the fixed and time-varying covariates [27].

1.3 Weighted Maximum Likelihood Estimation and Testing

Three versions of L-GoM were estimated for the Predictors 2 Study (Table A.1). For all three we set $K = 4$ which was established as the minimum acceptable size for *K* in prior analyses of the Predictors 1 and 2 cohorts where $K = 4$ was overwhelmingly favored over alternatives $K = 1$, 2, or 3 using AIC and BIC model selection rules [13]. $K = 4$ was also strongly preferred over $K = 5$ using AIC and BIC rules for suspected AD cases in the National Long Term Care Survey in our (unpublished) reanalysis of data compiled by Kinosian et al. [28], who had selected $K = 5$ over $K = 4$ based on Wilks' [29] chi-squared test (Wilks' test). Another application of GoM to cross-sectional AD registry data using Wilks' test reported $K = 5$ for neuropsychological data and $K = 6$ for clinical data [30]. Stallard and Sloan [9] noted that Wilks' test effectively cuts the AIC penalty (i.e., twice the degrees of freedom; or, equivalently, twice the number of free parameters) in half for large *J*—which explained the different *K*-values resulting from Wilks' test and the AIC rule. Our choice of $K = 4$ for the Predictors Study was strongly supported by the AIC and BIC rules and is consistent with the principle of parsimony. There was no support for $K < 4$.

Model 1 (M1) was based on 80 internal variables which included the mortality/survival indicator, six fixed covariates, and 73 time-varying covariates (all had $p < 0.05$; all variable-specific *p*-values were computed using Wilks' test for $K = 1$ vs. $K = 4$). In addition, six time-varying summary variables were fitted separately as an external set; the associated λ-parameters were estimated conditionally on the *g*- and *v*-parameters previously estimated with the 80 internal variables for Model 1; all six were statistically highly significant ($p < 0.001$). The 73 internal time-varying covariates constituted a comprehensive set of variables spanning 11 symptom domains, including cognition, functioning, dependence, and behavior (see Table A.2). This set included all covariates that had been established as significant L-GoM predictors in prior analyses of the Predictors 1 and 2 cohorts [13]. The overall log-likelihood for Model 1 was −62,737.93 which was separated into two parts: −8,443.05 for Exam 1 and −54,294.88 for Exams 2–21.

Model 2 (M2) was the optimal GoM solution for Exam 1 alone, i.e., it was estimated without consideration of Exams 2–21 or, equivalently, using eqn. (S.4) with $\Theta_{ij} \equiv 0$ for all *i, j*. The log-likelihood for Model 2 was −7,258.60, representing an improvement of 1,184.45 over the Model 1 results for Exam 1, with 1,191 d.f. The AIC rule accepts Model 1 over Model 2 because the log-likelihood improvement was smaller than its d.f. In contrast, the log-likelihood improvement was

statistically highly significant under Wilks' test (χ^2 = 2368.90; 1191 d.f.; $p << 0.001$), which rejects Model 1 in favor of Model 2.

Model 3 (M3) was designed to improve the fit for Exam 1 over the fit obtained from Model 1 for two reasons: (1) to exploit Exam 1's status as the single examination whose GoM scores were most likely to span the full range of the GoM-score continuum between the prognostic subtypes; and (2) to facilitate the use of the results in subsequent personalized predictive modeling applications for which the only available data were from a single exam at or shortly after diagnosis which would substitute for Exam 1 in the GoM-score estimation. Model 3 was estimated using weighted MLEs [31, 32] for which the relative weights for Exam 1 vs. Exams 2–21 were increased from 1.000 to 3.993, at which point the loss in both the weighted and unweighted log-likelihood fit for Exams 2–21 (1,175) exactly equaled the number of parameter constraints implicitly induced by the unequal weighting in Model 3. Assuming that these conditions are maintained as the sample size increases, we can ensure that Model 3 has the same limit point as Model 1, and, hence, the same asymptotic properties as Model 1; see [9] for discussion of these properties.

Table A.1 shows that the overall difference in log-likelihood fit between Model 1 and Model 3 (618.63) was statistically nonsignificant under Wilks' test (χ^2 = 1237.26; 1175 d.f; $p \geq 0.05$). More importantly, the difference in log-likelihood fit between Model 2 and Model 3 for Exam 1 (628.08) was also statistically nonsignificant (χ^2 = 1256.16; 1191 d.f.; $p \geq 0.05$). Hence, Model 3 provides an acceptable fit to Exam 1 under Wilks' test whereas Model 1 fails to do so. In other words, Model 3 outperforms Model 1 with respect to Exam 1—and it provides an acceptable overall fit for all examinations. Hence, Model 3 is our preferred model; it constitutes the L-GoM model in the main text.

Comment 1—Akaike [33] showed that ML estimates are biased, i.e., the estimated log-likelihood exceeds the expected log-likelihood by an amount approximately equal to the number of free parameters (k) in the model, leading to his celebrated formula: $\mathrm{AIC} = -2\ln L + 2k$. When multiple models are considered, the optimal estimates are obtained from the model with the largest expected log-likelihood or, equivalently, with the smallest AIC statistic. Hu and Zidek [32] presented a general approach for selecting optimal data-dependent weights in the context of weighted ML estimation for multiple independent but similar populations using Akaike's [33] approach as their starting point. We used Akaike's [33] approach to constrain the expected log-likelihood for Model 3 to exceed the expected log-likelihood for Model 1 using the data-dependent weights defined above.

Comment 2—We used Wilks' [29] chi-squared test to establish that Model 3 was an acceptable alternative to Model 1 and that Model 3 (Exam 1 only) was an acceptable alternative to Model 2. It follows, by inverting Wilks' test [34], that: (1) Model 3 lies within the 95% confidence region for Model 1; and (2) Model 3 (Exam 1 only) lies within the 95% confidence region for Model 2.

Comment 3—The Bayesian approach to mixed membership modeling [35] can be applied to L-GoM estimation if sufficient information is available to specify the Bayesian prior distributions. Wang [36] showed that weighted ML estimation can approximate Bayesian estimation when the information needed to specify the prior distributions is lacking. Our application of L-GoM aimed to estimate the GoM scores for individual AD patients enrolled in Predictors 2 and to do so using weighted ML estimation without having to specify a prior distribution for the GoM scores. The resulting GoM-score estimates (Table A.6) can be used to inform specification of the prior distribution of these scores for subsequent Bayesian analyses of new AD patient populations, providing an alternative to the standard Bayesian assumption that the prior distribution is Dirichlet—an assumption typically made because of its mathematical tractability rather than its external validity [37, 38].

1.4 Summary Statistics

Table A.2 displays the summary statistics for the 80 internal and six external variables in Model 3. The 73 time-varying variables are grouped into 11 measurement domains with domains 9–11 representing extensions to our prior model [13]. *APOE* (fixed internal) and *CDR* (time-varying external) were also added to our prior model. The key items for each variable *j* are Wilks' [29] chi-squared statistic and its d.f., which jointly yield the *p*-value. The AIC statistic was calculated as chi-squared minus twice its d.f. The BIC statistic was calculated as chi-squared minus its d.f. times the natural logarithm of the number of respondents, i.e., $\ln(N_j)$. Myoclonus was the sole variable with a nonsignificant *p*-value (i.e., $p \geq 0.05$) or a negative AIC statistic in Model 3; all 80 *p*-values were significant in Model 1. Fourteen variables, including all fixed variables except sex, had negative BIC statistics in Model 3. In other words, there was overwhelming evidence to support the inclusion of 66 of the 80 internal variables in Model 3. The support for the final 14 was absent under the BIC rule but present for the AIC rule and *p*-values, except for myoclonus.

The Kullback and Leibler [39] information statistic H_{jk} summarizes the contribution of variable *j* to subtype *k* using $H_{jk} = \sum_{l=1}^{R_j} \lambda_k^{jl} \ln(\lambda_k^{jl} / \bar{\pi}_{jl}) = \sum_{l=1}^{R_j} \lambda_k^{jl} \left[\ln(\lambda_k^{jl}) - \ln(\bar{\pi}_{jl})\right]$, where $\bar{\pi}_{jl}$ is the observed rate over all subjects and examinations for response *l*, $l = 1,\ldots, R_j$, where R_j is the number of responses to variable *j*. Rao ([40]; Lemma 2) proved that $H_{jk} \geq \frac{1}{2}\sum_{l=1}^{R_j} \lambda_k^{jl} (\lambda_k^{jl} - \bar{\pi}_{jl})^2$ which is half the mean squared deviation of the model-based subtype probabilities from the observed marginal probabilities. Because the maximum deviation is 1, it follows that $\sum_{l=1}^{R_j} \lambda_k^{jl} (\lambda_k^{jl} - \bar{\pi}_{jl})^2 \leq 1$. We interpret $H_{jk} > 1/2$ as indicating relatively large deviations between the model-based and the observed marginal probabilities; variables meeting this criterion are indicated in boldface red font in Table A.2. Three fixed variables with negative BIC statistics —*APOE*, *Age at Intake,* and

Occupation—had at least one $H_{jk} > 1/2$, indicating that the BIC criterion alone may be too stringent, explaining why we continue to present the AIC statistics and *p*-values.

The top ranked predictor using the BIC or AIC statistics, or the *p*-values, was *Equivalent Institutional Care*, which was the source variable for *FTC*. Among the external variables, the largest BIC and AIC statistics (and smallest *p*-values) were for *Dependence Scale Score*, *BDRS Score*, *CDR Rating*, *MMSE Score*, and *Psychiatric Symptoms*, in that order.

1.5 *λ*-Parameters

Table A.3 displays the *λ*-parameters for the 80 internal and six external variables in Model 3. The *λ*-parameters for variables with $H_{jk} > 1/2$ are indicated in boldface red font. Also displayed are the expected [$E(\pi_i^{jl})$] and observed ($\bar{\pi}_{jl}$) marginal probabilities for the 222 outcomes which though not identical were generally quite close (correlation = 99.90%). The *λ*-parameters for the external variables indicate that subtypes 1 and 4 were distinguished by extremes of dependency levels. Subtype 3 was distinguished by high levels of behavioral and psychiatric symptoms, and depression, which declined over time as affected patients progressed towards subtype 4. Subtype 2 was distinguished from subtype 1 by a higher likelihood of being female, age 80+ at intake, having MMSE below 24 at intake, and homozygous APOE e4/e4, as well as substantially lower total and disability-free life expectancies (Table 3).

1.6 Transition Matrices and Pure-Subtype Trajectories

Table A.4 displays the cumulative transition matrices, $\mathbf{V}_t$, for the 21 examinations. Eqns. (A.3) and (A.4) show how these matrices may be combined with either the *λ*-parameters or the GoM scores to represent the temporal progression of AD. The GoM-score trajectories, defined using $\boldsymbol{\gamma}_{it} = \mathbf{V}_t^T \mathbf{g}_i$, are weighted combinations of the *K* pure-subtype trajectories. Hence, the *K* pure-subtype trajectories (Table A.5) are in one-to-one correspondence with the *K* rows of the transition matrices, i.e., the sequence of top rows, taken as a set, forms the pure-subtype trajectory for subtype 1; the sequence of second rows, taken as a set, forms the pure-subtype trajectory for subtype 2; etc.

1.7 GoM Scores

Table A.6 displays, for the 229 participants in Predictors 2, the age group at intake, sex, subgroup assignment, GoM scores, and the complete set of individual-specific LE and probability measures summarized in Tables 1, S.1, and S.2. These may be used in combination with the parameters in Tables A.3 and A.4 to reproduce the predicted outcomes in this paper as well as to simulate other outcomes not reported herein but covered by the variables in Table A.3.

1.8 Subgroups vs. Subtypes

Subgroups are distinct from subtypes in L-GoM. Subgroups 1–4 were defined by high proximity to L-GoM subtypes 1–4; subgroup 0 was defined by low proximity to all subtypes. Subgroup 2 was the largest ($N = 87$) and subgroup 4 the smallest ($N = 11$). Table A.7 displays the means and standard deviations of the GoM scores by subtype, both overall and by subgroup. The subgroup means ranged from 0.008 to 0.774, with the highest values along the main diagonal of the table where the subgroup and subtype indexes are equal. The subgroup standard deviations ranged from 0.027 to 0.173. The subtype means ("Total" row) were largest (0.397) for subtype 2 and smallest (0.099) for subtype 4. The subtype standard deviations ("Total" row) ranged from 0.169 for subtype 4 to 0.324 for subtype 1. The subgroup standard deviations were substantially smaller because the subgroups were substantially more homogeneous, due to their rational design [41], than the overall study sample.

1.9 Mortality and Disability Calculations

The observed survival values in Figure 2 were computed using the Kaplan-Meier product-limit estimator [21] with mortality recorded at the time of the next scheduled exam following the date of death. The estimated survival probabilities were computed by averaging the individual survival probabilities within subgroups, where the individual survival values at time *t* were computed as products of the p_{it}'s, using $\prod_{\tau=0}^{t-1} p_{i\tau}$, as in eqns. (S.1) and (S.2), parametrized as $p_{i\tau} = \sum_{k=1}^{K} g_{ik} \tilde{p}_{k\tau}$. The simultaneous bands were developed by Nair [42] for Kaplan-Meier survival curves [21]. The pointwise bands were tighter, but the estimated values still performed well with these bands. Both sets of confidence bands were calculated using the adjusted Wald methodology [43] which was recommended for binomial confidence intervals for small sample sizes and/or small probabilities, as in Figure 2.

The estimated FTC rates in Figure 3 were computed by averaging the individual FTC rates within subgroups, where the individual values were estimated using the π_{it}'s in eqn. (S.1), parametrized as $\pi_{it} = \sum_{k=1}^{K} g_{ik} \tilde{\pi}_{kt}$. The individual-specific estimated FTC rates increased monotonically over time; the occasional reversals for subgroups in Figure 3 were due to temporal changes in the composition of the subgroups due to different patterns of missing FTC assessments among survivors over time.

The estimated average survival probabilities on the left side of Figure 4 were computed as in Figure 2. The estimated average survival probabilities on the right side of Figure 4 are averages of individual-specific survival probabilities each of which was computed using Sullivan's method to apply the individual's estimated FTC-disability-free probabilities to his/her overall survival

function values at each examination; i.e., for each individual *i* at time *t*, the FTC-disability-free survival probability was computed as $\left(\prod_{\tau=0}^{t-1} p_{i\tau}\right)(1-\pi_{it})$, as in eqn. (S.2).

1.10 Standard Error Calculations

The mean values in Table S.2 were computed as probability-weighted expected values of the indicated conditional life expectancy estimates for individuals, using the corresponding probabilities defined in Table S.1 as the weights. Potthoff et al. [44] showed that the usual calculation of the standard errors for the case of differential weighting was downwardly biased. We used their eqn. (1.11) to remove this bias.

2. ASSOCATED IRREVERSIBLE DISABILITY MODEL

The conditional DFLEs and DLEs are supplementary outputs from the SLT/L-GoM model; they are not required for calculating the Sullivan DFLEs or DLEs. They are, however, important outputs from standard transition models. We present the technical details of their calculations for completeness and to facilitate comparisons with similar outputs from other models.

Figure S.1 displays the states and transitions for the associated irreversible disability model with three states: nondisabled, disabled, and dead. Each individual AD patient *i* is assumed to meet the NINCDS-ADRDA criteria [45] at time $T_{i0}=t_{i0}$, to enter the Predictors 2 Study Cohort at time $T_{i1}=t_{i1}\geq t_{i0}$, to transition to disability at time $T_{i2}=t_{i2}>t_{i0}$, and to die at time $T_{i3}=t_{i3}>t_{i1}$. By convention, we use upper case to denote random times and lower case to denote realized times. The time of onset of AD (t_{i0}) will generally be at or near the time of study intake (t_{i1}). The transition to disability may occur prior to study intake, as shown for FTC in Figure 4.

Three sets of transition rates, $\mu_i(t)$, $\nu_i(t)$, and $\upsilon_i(t)$, governing nondisabled mortality, disability onset, and disabled mortality, respectively, operate in continuous time as individual-specific functions of *t*. L-GoM's conditional independence assumption implies that $\mu_i(t)\equiv\upsilon_i(t)$, given the GoM scores for individual *i*. The transition rates generate the conditional DFLEs and DLEs as shown in the following paragraphs.

2.1 Conditional DFLE

Let

$$\pi_i^c(t;t_{i0}) = \Pr(i \text{ is nondisabled at } t \mid i \text{ is alive at } t) \quad \text{(A.6a)}$$

$$= \exp\left(-\int_{t_{i0}}^{t} \nu_i(\tau)d\tau\right) \quad \text{(A.6b)}$$

and $\pi_i(t;t_{i0}) = 1 - \pi_i^c(t;t_{i0})$. By setting $t_{i1} = 0$, we obtain $\pi_i(t_{i1};t_{i0}) = \pi_{i0}$, the disability rate at intake in eqn. (S.1), which implies that $t_{i1} \equiv 0$ is the natural origin of the time dimension for individual *i*, which will be assumed henceforth. The conditional DFLE (equivalently, conditional ALE [4]) for individual *i* at time t_{i1}, given that individual *i* is alive and nondisabled at that time, is defined as:

$$e_{\mathrm{CA}i} = \int_{t_{i1}}^{\infty} \exp\left(-\int_{t_{i1}}^{t} \left[\nu_i(\tau) + \mu_i(\tau) \right] d\tau \right) dt \tag{A.7a}$$

$$= \int_{t_{i1}}^{\infty} \exp\left(-\int_{t_{i1}}^{t} \mu_i(\tau) d\tau \right) \frac{\pi_i^c(t;t_{i0})}{\pi_i^c(t_{i1};t_{i0})}\, dt \tag{A.7b}$$

$$= \frac{e_{\mathrm{A}i}}{\pi_{i0}^c} \tag{A.7c}$$

where $e_{\mathrm{A}i}$ is the continuous-time version of $e_{\mathrm{A}i}$, defined in eqn. (S.2), with the upper limit of integration extended from ω to ∞. Hence,

$$e_{\mathrm{A}i} = \pi_{i0}^c \times e_{\mathrm{CA}i} + \pi_{i0} \times 0, \tag{A.8}$$

which shows that the Sullivan DFLE (e.g., Table 3; col. 3) is a weighted average of (1) the conditional DFLE for individuals free of disability at intake (e.g., Table S.2; col. 2) and (2) zero for individuals who are disabled at intake.

2.2 Conditional DLE

The Sullivan DLE and the conditional DLE are similarly related. Let

$$\Pi_i(t_{i1};t_{i0}) = \Pr(i \text{ is/becomes disabled at } t_{i1} \text{ or later} \mid i \text{ is alive at } t_{i1}) \tag{A.9a}$$

$$= \pi_i(t_{i1};t_{i0}) + \pi_i^c(t_{i1};t_{i0}) \int_{t_{i1}}^{\infty} \nu_i(t)\, \mathrm{e}^{-\int_{t_{i1}}^{t} [\nu_i(\tau)+\mu_i(\tau)] d\tau}\, dt, \tag{A.9b}$$

where e^x denotes the exponential function $\exp(x)$, and

$$\Pi_i^c(t_{i1};t_{i0}) = \Pr(i \text{ is nondisabled at } t_{i1} \text{ and remains so thereafter} \mid i \text{ is alive at } t_{i1}) \tag{A.10a}$$

$$= 1 - \Pi_i(t_{i1};t_{i0}) \tag{A.10b}$$

which simplifies to $\Pi_i(t_{i1};t_{i0}) = \Pi_{i0}$ and $\Pi_i^c(t_{i1};t_{i0}) = \Pi_{i0}^c$ using $t_{i1} \equiv 0$. The conditional DLE for individual *i* at time t_{i1}, given that individual *i* is alive at that time and is or becomes disabled prior to death, is defined as:

$$e_{\mathrm{CD}i} = \frac{\pi_i(t_{i1};t_{i0}) \int_{t_{i1}}^{\infty} \mathrm{e}^{-\int_{t_{i1}}^{t} \upsilon_i(\tau)d\tau} \, dt \; + \; \pi_i^c(t_{i1};t_{i0}) \int_{t_{i1}}^{\infty} \nu_i(t) \, \mathrm{e}^{-\int_{t_{i1}}^{t} [\nu_i(\tau)+\mu_i(\tau)]d\tau} \int_{t}^{\infty} \mathrm{e}^{-\int_{t}^{z} \upsilon_i(\tau)d\tau} \, dz dt}{\Pi_i(t_{i1};t_{i0})} \qquad \text{(A.11a)}$$

$$= \frac{e_{\mathrm{D}i}}{\Pi_{i0}} \qquad \text{(A.11b)}$$

where one can show via integration by parts that $e_{\mathrm{D}i}$ is the continuous-time version of the quantity $e_{\mathrm{D}i}$, defined in eqn. (S.1), with the upper limit of integration extended from ω to ∞. Hence,

$$e_{\mathrm{D}i} = \Pi_{i0} \times e_{\mathrm{CD}i} + \Pi_{i0}^c \times 0 , \qquad \text{(A.12)}$$

which shows that the Sullivan DLE (e.g., Table 3; col. 4) is a weighted average of (1) the conditional DLE for individuals who are disabled at intake or become disabled some time later (e.g., Table S.2; col. 6) and (2) zero for individuals who never become disabled (because they die prior to onset of disability).

2.3 Alternative Decomposition of Sullivan DLE

The additive form of the numerator in eqn. (A.11a) yields an informative alternative decomposition of $e_{\mathrm{D}i}$. Let

$$e_{\mathrm{CD}i}^{\mathrm{D}} = \int_{t_{i1}}^{\infty} \exp\left(-\int_{t_{i1}}^{t} \upsilon_i(\tau)d\tau \right) dt \qquad \text{(A.13)}$$

and

$$e_{\mathrm{CD}i}^{\mathrm{A}} = \frac{\pi_i^c(t_{i1};t_{i0}) \int_{t_{i1}}^{\infty} \nu_i(t) \, \mathrm{e}^{-\int_{t_{i1}}^{t} [\nu_i(\tau)+\mu_i(\tau)]d\tau} \int_{t}^{\infty} \mathrm{e}^{-\int_{t}^{z} \upsilon_i(\tau)d\tau} \, dz dt}{\Pi_i^{\mathrm{A}}(t_{i1};t_{i0})} , \qquad \text{(A.14)}$$

where

$$\Pi_i^{\mathrm{A}}(t_{i1};t_{i0}) = \Pr(i \text{ is nondisabled at } t_{i1} \text{ but becomes disabled beyond } t_{i1} \mid i \text{ is alive at } t_{i1}) \quad \text{(A.15a)}$$

$$= \pi_i^c(t_{i1};t_{i0}) \int_{t_{i1}}^{\infty} \nu_i(t) \exp\left(-\int_{t_{i1}}^{t} \left[\nu_i(\tau) + \mu_i(\tau) \right] d\tau \right) dt \quad \text{(A.15b)}$$

$$= \Pi_i(t_{i1};t_{i0}) - \pi_i(t_{i1};t_{i0}). \quad \text{(A.15c)}$$

Then, assuming that $t_{i1} \equiv 0$, eqn. (A.15c) simplifies to $\Pi_i^{\mathrm{A}}(t_{i1};t_{i0}) = \Pi_{i0}^{\mathrm{A}} = \Pi_{i0} - \pi_{i0}$ with $\Pi_{i0} = \pi_{i0} + \Pi_{i0}^{\mathrm{A}}$, and eqn. (A.12) can be rewritten as:

$$e_{\mathrm{D}i} = \pi_{i0} \times e_{\mathrm{CD}i}^{\mathrm{D}} \ + \ \Pi_{i0}^{\mathrm{A}} \times e_{\mathrm{CD}i}^{\mathrm{A}} + \Pi_{i0}^{c} \times 0 , \quad \text{(A.16)}$$

which shows that the Sullivan DLE can be decomposed into a weighted sum of (1) the conditional DLE for individuals who are disabled at intake, $e_{\mathrm{CD}i}^{\mathrm{D}}$, with probability π_{i0}, and (2) an alternative conditional DLE defined for individuals who are nondisabled at intake but become disabled at some later time, $e_{\mathrm{CD}i}^{\mathrm{A}}$, with probability Π_{i0}^{A}—the numerator of the corresponding conditional probability given by $\Pi_{i0}^{\mathrm{A}} / \pi_{i0}^{c}$. Moreover, eqns. (A.12) and (A.16) jointly imply that:

$$e_{\mathrm{CD}i} = \frac{\pi_{i0}}{\Pi_{i0}} \times e_{\mathrm{CD}i}^{\mathrm{D}} \ + \ \frac{\Pi_{i0}^{\mathrm{A}}}{\Pi_{i0}} \times e_{\mathrm{CD}i}^{\mathrm{A}} , \quad \text{(A.17)}$$

which expresses the original conditional DLE, $e_{\mathrm{CD}i}$, (e.g., Table S.2; col. 6) as a weighted average of $e_{\mathrm{CD}i}^{\mathrm{D}}$ and $e_{\mathrm{CD}i}^{\mathrm{A}}$ (e.g., Table S.2; cols. 7 and 8). For small π_{i0} and large Π_{i0}^{A}, $e_{\mathrm{CD}i}^{\mathrm{A}} \approx e_{\mathrm{CD}i}$ (e.g., see Table A.6).

Table A.1. Model Selection Statistics

Scope	Model 1 (M1)	Model 2 (M2)	Model 3 (M3)	M3–M1
		Log-Likelihood		
Exam 1	-8,443.05	-7,258.60	-7,886.68	556.37
Exams 2–21	-54,294.88	–	-55,469.88	-1,175.00
Exams 1–21	-62,737.93	–	-63,356.56	-618.63
		Expected Log-Likelihood		
Exam 1	–	-8,449.60	–	
Exams 1–21	-64,112.93	–	-63,556.56	556.37
		Number of Free Parameters		
Exam 1	–	1,191	–	
Exams 1–21	1,375	–	200	-1,175
		Number of Observed Data Points		
Exam 1 / 1–21	121,131	16,149	121,131	0

Notes: M3 was estimated using weights of 3.993 and 1.000 for observations in Exam 1 and Exams 2–21, respectively; the log-likelihood results for M3 shown above are unweighted. The expected log-likelihoods for M1, M2, and M3 were computed as the log-likelihood minus the number of free parameters, i.e., degrees of freedom (d.f.) (Akaike, 1973). M1 had 1,375 free parameters: 80 λ's for the 6 fixed variables, 488 λ's for the 73 time-varying variables and mortality/survival, 687 GoM scores, and 120 u-parameters in the transition matrices. M2 had 1,191 free parameters: 80 λ's for the 6 fixed variables, 424 λ's for the 73 time-varying variables and mortality/survival, and 687 GoM scores. M3 imposed constraints on 1,175 parameters used for Exams 2–21 in the M1 likelihood: 488 λ's for the 73 time-varying variables and mortality/survival, and 687 GoM scores. M3 had 200 free parameters: 80 λ's for the 6 fixed variables and 120 u-parameters in the transition matrices. To account for the 1,175 constraints in M3, the log-likelihood component for Exams 2–21 in M3 was reduced by 1,175.00, from −54,294.88 to −55,469.88, by increasing the Exam 1 weights from 1.000 to 3.993, which allowed the log-likelihood component for Exam 1 to increase by 556.37. Under this procedure, the overall M3 expected log-likelihood value was guaranteed to always exceed the overall M1 expected log-likelihood value, and to do so by an amount equal to the increase in the log-likelihood component for Exam 1 (e.g., 556.37, as shown above).

Table A.2. Summary Statistics for 80 Internal and Six External Variables, Model 3, Predictors 2 Study Cohort

j	Domain	Name	Description	R_j	N_j	H_{j1}	H_{j2}	H_{j3}	H_{j4}	p-Value	df	Chi-Sq	AIC	BIC
				Internal Variables										
0	—	ProsSurv	Prospective 6-Month Survival	2	1976	0.0433	0.0627	0.0627	0.0609	5.0E-19	3	88.33	82.33	65.57
1	—	APOE_RC_F	APOE RECODE	4	169	0.0339	0.1231	0.1980	**0.5218**	1.3E-02	9	20.97	2.97	-25.20
2	—	B01_F	Sex	2	229	0.0468	0.2241	0.2078	0.0038	7.0E-05	3	21.85	15.85	5.55
3	—	B02_RC_F	Age at Intake: RECODE	6	229	0.2275	0.0415	0.3055	**1.1393**	4.0E-05	15	46.80	16.80	-34.71
4	—	Race_F	Race RECODE	2	228	0.0001	0.0775	0.1364	0.0775	4.3E-03	3	13.16	7.16	-3.13
5	—	Occup_RC_F	Occupation RECODE	8	228	0.2068	0.1738	**0.5225**	**0.6556**	1.5E-03	21	45.42	3.42	-68.60
6	—	Diagnosis_Lag_RC_F	Years Since Diagnosis RECODE	4	214	0.1920	0.0424	0.4855	0.3446	1.7E-03	9	26.42	8.42	-21.87
7	1	PP43	Wandered Away	2	1772	0.0922	0.0401	0.0019	0.0277	1.2E-14	3	67.84	61.84	45.40
8	1	PP44	Verbal Outbursts	2	1771	0.2811	0.2811	**1.3866**	0.1170	6.1E-66	3	305.60	299.60	283.16
9	1	PP45	Physical Threats	3	1771	0.1066	0.1066	0.2242	0.0575	4.1E-30	6	151.28	139.28	106.41
10	1	PP51_RC	Difficulty Sleeping	3	449	0.1299	0.0185	0.0415	0.0444	1.9E-04	6	26.38	14.38	-10.26
11	2	MMSE_GATE	Overall MMSE Gatekeeper	2	1908	0.1550	0.2982	0.1969	0.3150	1.7E-75	3	349.68	343.68	327.02
12	2	Orientation_RC	Sum of Orientation Variables RECODE	3	1416	**0.8035**	0.1171	0.1185	**0.6235**	1.1E-106	6	508.78	496.78	465.24
13	2	SP11B	MMSE -- Name 3 Objects	4	1485	0.0750	0.1808	0.0943	0.2251	1.0E-28	9	154.54	136.54	88.81
14	2	SP16B	MMSE -- World	6	1477	0.2482	0.1160	0.3124	0.3742	1.1E-50	15	279.25	249.25	169.79
15	2	SP18B	MMSE -- Ask for 3 Objects	4	1480	0.1917	0.0091	0.0676	0.0952	2.3E-25	9	138.35	120.35	72.65
16	2	Language	Sum of Language Variables	9	1416	0.2296	0.1697	0.1542	0.4080	1.4E-43	24	270.53	222.53	96.39
17	2	SP41B	MMSE -- Intersecting Pentagons-MMS	2	1436	0.3567	0.0003	0.0710	**0.6014**	8.7E-73	3	337.23	331.23	315.42
18	3	NN01	Patient Trouble with Chores	3	1818	**1.0409**	0.2468	0.1284	**0.5547**	1.9E-183	6	864.35	852.35	819.32
19	3	NN02	Patient Trouble Handling Money	3	1816	**0.9937**	0.2915	0.2318	**0.5661**	6.4E-183	6	861.91	849.91	816.88
20	3	NN03	Patient Trouble Remembering Lists	3	1818	**0.6916**	0.0133	0.1319	0.1746	6.0E-71	6	342.59	330.59	297.55
21	3	NN04	Patient Trouble Around House	3	1817	0.4928	0.4928	0.2138	**0.9462**	4.9E-182	6	857.81	845.81	812.78
22	3	NN05	Patient Trouble Around Neighborhood	3	1813	**0.8487**	**0.6221**	0.4233	**0.7801**	1.4E-236	6	1110.01	1098.01	1064.99
23	3	NN06	Patient Trouble Recognizing Place	3	1815	**0.5266**	**0.5266**	0.3490	**0.6049**	5.0E-180	6	848.55	836.55	803.52
24	3	NN07	Patient Trouble Remembering Things	3	1818	**0.6786**	0.0611	0.0927	0.2362	4.1E-76	6	366.67	354.67	321.64
25	3	NN08	Patient Dwells in the Past	3	1811	0.2892	0.2445	**0.8550**	0.0361	1.9E-34	6	171.74	159.74	126.73
26	3	NN09	Patient Eating	4	1811	0.3644	0.3644	0.1127	0.4670	8.8E-115	9	559.80	541.80	492.28
27	3	NN10	Patient Dressing	4	1811	**0.7984**	0.4966	0.4156	**0.7425**	3.2E-252	9	1198.08	1180.08	1130.56
28	3	NN11	Patient Bladder and Bowel Control	4	1810	0.3822	0.4328	0.1679	**0.7088**	2.1E-160	9	772.10	754.10	704.59
29	3	NN12	Increased Rigidity	2	1776	0.0230	0.0933	**0.8370**	0.0719	1.7E-41	3	192.57	186.57	170.13
30	3	NN13	Increased Egocentricity	2	1776	0.2799	0.1358	**0.9914**	0.0567	4.0E-54	3	250.98	244.98	228.54
31	3	NN14	Impairment of Regard for Feelings of Others	2	1777	0.3510	0.3510	**1.0791**	0.0201	9.4E-59	3	272.40	266.40	249.95
32	3	NN15	Coarsening of Affect	2	1778	0.0975	0.1194	**1.1359**	0.1375	3.6E-49	3	228.08	222.08	205.63
33	3	NN16	Impairment of Emotional Control	2	1778	0.1125	0.0538	**0.9437**	0.1573	3.9E-61	3	283.38	277.38	260.93
34	3	NN17	Hilarity in Inappropriate Situations	2	1777	0.1493	0.1924	0.3777	0.0296	9.9E-28	3	128.79	122.79	106.34
35	3	NN18	Diminished Emotional Responsiveness	2	1776	**0.5294**	0.1133	0.3943	0.0223	4.4E-48	3	223.07	217.07	200.62
36	3	NN19	Sexual Misdemeanor	2	1768	0.0307	0.1146	**0.5620**	0.0636	1.5E-20	3	95.42	89.42	72.99
37	3	NN20	Hobbies Relinquished	2	1775	**0.5372**	0.0031	0.1157	0.0729	3.7E-53	3	246.50	240.50	224.06
38	3	NN21	Diminished Initiative/Growing Apathy	2	1778	0.3783	0.0254	0.3365	0.0267	5.5E-40	3	185.59	179.59	163.14
39	3	NN22	Purposeless Hyperactivity	2	1773	0.0649	0.2508	0.4741	0.0184	8.5E-29	3	133.74	127.74	111.30
40	4	RR01	Needs Reminders	3	1777	**0.9436**	0.0231	0.1604	0.1160	4.4E-71	6	343.22	331.22	298.33
41	4	RR02	Needs Help to Remember	3	1779	**0.5554**	0.0393	0.1162	0.1123	7.4E-57	6	276.87	264.87	231.97
42	4	RR03	Needs Help Finding Things	2	1779	0.2545	0.0050	0.0807	0.0683	3.0E-39	3	182.17	176.17	159.71
43	4	RR04	Needs Household Chores Done	2	1779	**1.3332**	0.0806	0.3060	0.3060	1.3E-174	3	807.03	801.03	784.58
44	4	RR05	Needs Watching When Awake	2	1784	**0.5919**	**0.5919**	0.0123	**0.8058**	1.0E-197	3	913.55	907.55	891.09
45	4	RR06	Needs to be Escorted When Outside	2	1786	**0.9239**	**0.8455**	0.2093	**0.5058**	1.3E-201	3	931.46	925.46	908.99
46	4	RR07	Needs to be Accompanied Bathing/Eating	2	1785	**0.5528**	**0.5528**	0.2837	**0.8565**	1.1E-243	3	1125.49	1119.49	1103.03
47	4	RR08	Needs to be Dressed/Washed/Groomed	2	1781	0.4117	0.4117	0.4117	**1.0863**	3.9E-236	3	1090.67	1084.67	1068.21

48	4	RR09	Needs to be Taken to Toilet	2	1780	0.3212	0.3212	0.3212	**0.7362**	1.3E-181	3	839.34	833.34	816.88
49	4	RR10	Needs to be Fed	2	1781	0.0923	0.0923	0.0923	0.0983	5.5E-56	3	259.59	253.59	237.14
50	4	RR11	Needs to be Turned/Moved/Transferred	2	1782	0.0837	0.0837	0.0837	0.0970	2.8E-50	3	233.23	227.23	210.77
51	4	RR12	Needs to Wear Diaper/Catheter	2	1782	0.3395	0.3395	0.3395	**0.7269**	7.2E-155	3	715.97	709.97	693.52
52	4	RR13	Needs to be Tube Fed	2	1781	0.0107	0.0107	0.0107	0.0136	1.0E-04	3	21.06	15.06	-1.39
53	4	RR15	Equivalent Institutional Care	3	1771	**1.2255**	0.4540	**0.9254**	**0.6769**	1.6E-268	6	1257.61	1245.61	1212.73
54	4	CC08_A_RC	Living Status RECODE	5	1849	0.2663	0.2435	0.2960	0.4686	6.6E-96	12	483.67	459.67	393.40
55	4	INST_LOS_RC	Years Since Entering LTC Facility RECODE	6	383	**0.9467**	**0.7528**	0.4363	0.1085	6.4E-05	15	45.50	15.50	-43.72
56	5	FF10	Adequate Sight?	2	525	0.0367	0.0021	0.0731	0.0685	2.0E-05	3	24.49	18.49	5.69
57	5	FF11	Adequate Hearing?	2	524	0.1003	0.0058	0.1003	0.1418	2.4E-06	3	28.84	22.84	10.05
58	6	EF07	Admission to Hospital	2	1635	0.0095	0.0612	0.0080	0.0093	5.2E-04	3	17.65	11.65	-4.55
59	6	EF08	Treatment	2	1634	0.0106	0.0227	0.0113	0.0275	5.7E-05	3	22.30	16.30	0.10
60	6	KK16	Had Seizure?	2	1708	0.0072	0.0339	0.0000	0.0127	4.0E-04	3	18.21	12.21	-4.12
61	7	DELUSION	Delusions	2	1775	0.2602	0.2627	**0.7647**	0.0001	3.3E-48	3	223.60	217.60	201.16
62	7	HALLUCIN	Hallucinations	2	1772	0.1293	0.0580	0.0690	0.0103	4.5E-18	3	83.87	77.87	61.43
63	7	ILLUSION	Illusions	2	1758	0.0430	0.0430	0.0176	0.0071	1.4E-09	3	44.14	38.14	21.73
64	8	Beer_Wk_RC	Beer/Week: 0 - 99 RECODE	3	1180	0.0354	0.0345	0.0502	0.0345	3.6E-03	6	19.38	7.38	-23.06
65	8	Wine_Wk_RC	Wine/Week: 0 - 99 RECODE	3	1184	0.0190	0.1169	0.0854	0.1148	1.2E-13	6	72.51	60.51	30.05
66	8	Liquor_Wk_RC	Hard liquor/Week: 0 - 99 RECODE	3	1182	0.0244	0.0157	0.0123	0.0503	1.9E-04	6	26.32	14.32	-16.13
67	9	EPSXX	Extrapyramidal Symptoms	3	1437	0.4335	0.3109	0.1300	**0.8966**	5.6E-92	6	440.44	428.44	396.82
68	9	Tremor	Tremor	2	1508	0.0615	0.0412	0.0615	0.1452	5.5E-18	3	83.50	77.50	61.54
69	9	Bradykinesia	Bradykinesia	2	1465	0.0937	0.0937	0.0785	0.2064	3.7E-32	3	149.31	143.31	127.45
70	9	Gait	Gait	2	1460	0.1061	0.1061	0.1061	0.2771	5.1E-39	3	181.09	175.09	159.23
71	9	Myoclonus	Myoclonus	2	1515	0.0213	0.0116	0.0010	0.0094	4.0E-01	3	2.96	-3.04	-19.01
72	9	Rigidity	Rigidity	2	1512	0.1268	0.1268	0.1268	0.2836	3.2E-41	3	191.32	185.32	169.35
73	10	AGITATION	Agitation	2	1774	0.3183	0.3689	**0.9285**	0.0050	2.3E-65	3	302.97	296.97	280.52
74	10	SAD	Sadness/Depression	2	1772	0.0756	0.0144	**0.5882**	0.0541	2.9E-21	3	98.76	92.76	76.32
75	10	DEP_FREQ_RC	Depression Frequency	4	667	0.1300	0.2924	**0.7809**	0.0794	3.2E-05	9	36.49	18.49	-22.03
76	10	AP	Appetite Problems	2	1768	0.1275	0.0236	0.1238	0.0022	1.8E-12	3	57.74	51.74	35.31
77	11	DL_Gate	Lewy Bodies Gatekeeper	2	1918	0.0344	0.0724	0.0582	0.0816	2.0E-05	3	24.48	18.48	1.80
78	11	DL02	Fluctuating Cognition (Lewy)	2	1572	0.1223	0.0405	0.0171	0.0140	1.0E-07	3	35.41	29.41	13.33
79	11	DL03	Visual Hallucinations (Lewy)	2	1583	0.0912	0.0912	0.0001	0.0403	3.2E-18	3	84.58	78.58	62.48
Total				222	121131						426	24843	23991	21853
				External Variables										
1	2	SP51_RC	MMSE Score (Range 0-30) RECODE	3	1416	**0.7472**	0.2796	0.3217	**0.6040**	1.3E-110	6	527.02	515.02	483.49
2	3	NNTOT_RC	BDRS Score RECODE	4	1723	**0.9728**	**0.8781**	**0.6977**	**0.6536**	4.9E-248	9	1178.68	1160.68	1111.61
3	4	RR14	Dependence Scale Score	6	1776	**1.0821**	**0.6245**	**0.7741**	**0.8712**	1.2E-263	15	1279.83	1249.83	1167.60
4	8	Alcohol_Wk_RC	Alcoholic Drinks/Week RECODE	3	1179	0.0252	0.0853	0.1228	0.1499	6.5E-17	6	88.42	76.42	45.98
5	—	QQ01_RC	CDR Rating	6	1772	**0.5492**	**0.5188**	0.2428	**0.8868**	1.1E-192	15	948.98	918.98	836.78
6	7	PSYCHSX	Psychiatric Symptoms	2	1775	0.3281	0.2522	**0.9483**	0.0021	8.7E-54	3	249.42	243.42	226.98
Total				24	9641						54	4272	4164	3872

Notes: j denotes variable number; 11 domains are defined for the 73 time-varying variables; R_j is the number of response levels for j; N_j is the number of respondents for j; H_{j1}–H_{j4} are the Kullback-Leibler information statistics for the K=4 subtypes. Boldface red font indicates H_{jk} > 0.5.

Table A.3. λ-Parameters for 80 Internal and Six External Variables, Model 3, Predictors 2 Study Cohort

j	Name	Description	l	Label	N_{jl}	λ^{jl}_1	λ^{jl}_2	λ^{jl}_3	λ^{jl}_4	$E(\pi^{jl}_i)$	Obs. Rate
				Internal Variables							
0	ProsSurv	Prospective 6-Month Survival	1	Dies	120	0.0056	0.0000	0.0000	0.1598	0.0578	0.0607
			2	Survives	1856	0.9944	1.0000	1.0000	0.8402	0.9422	0.9393
1	APOE_RC_F	APOE RECODE	1	e4/e4	20	0.1004	0.1938	0.0611	**0.0000**	0.1185	0.1183
			2	e3/e4	71	0.3357	0.3732	0.6814	**0.3000**	0.4214	0.4201
			3	e3/e3	62	0.3976	0.4330	0.2574	**0.2412**	0.3668	0.3669
			4	e2/e3, e2/e4	16	0.1664	0.0000	0.0000	**0.4587**	0.0933	0.0947
2	B01_F	Sex	1	Male	91	0.5491	0.0994	0.7159	0.4403	0.3957	0.3974
			2	Female	138	0.4509	0.9006	0.2841	0.5597	0.6043	0.6026
3	B02_RC_F	Age at Intake: RECODE	1	49-64	20	0.1189	0.1396	0.0000	**0.0000**	0.0899	0.0873
			2	65-69	14	0.0000	0.0939	0.1090	**0.0000**	0.0605	0.0611
			3	70-74	48	0.4319	0.1282	0.1912	**0.0000**	0.2173	0.2096
			4	75-79	69	0.2942	0.2742	0.5140	**0.0000**	0.3041	0.3013
			5	80-84	46	0.1145	0.2069	0.1858	**0.4142**	0.1961	0.2009
			6	85+	32	0.0404	0.1572	0.0000	**0.5858**	0.1321	0.1397
4	Race_F	Race RECODE	1	White	211	0.9224	1.0000	0.7560	1.0000	0.9249	0.9254
			2	Non-white	17	0.0776	0.0000	0.2440	0.0000	0.0751	0.0746
5	Occup_RC_F	Occupation RECODE	1	Housewife	16	0.0549	0.0649	**0.0000**	**0.2704**	0.0685	0.0702
			2	Housewife,other	18	0.0321	0.1406	**0.0112**	**0.1184**	0.0790	0.0789
			3	Unskilled/semi-skl	14	0.0000	0.0000	**0.2775**	**0.0000**	0.0596	0.0614
			4	Skilled trade	12	0.1619	0.0000	**0.0000**	**0.0638**	0.0535	0.0526
			5	Clerical/office	43	0.1376	0.1958	**0.1986**	**0.2743**	0.1873	0.1886
			6	Manager bus/govt	39	0.0984	0.1600	**0.2327**	**0.2731**	0.1689	0.1711
			7	Professional	75	0.4710	0.4304	**0.1328**	**0.0000**	0.3355	0.3289
			8	Other	11	0.0441	0.0083	**0.1472**	**0.0000**	0.0478	0.0482
6	Diagnosis_Lag_ RC_F	Years Since Diagnosis RECODE	1	0	101	0.7025	0.5218	0.0579	0.5022	0.4728	0.4720
			2	1	55	0.2151	0.3255	0.3062	0.0000	0.2586	0.2570
			3	2	26	0.0000	0.0758	0.3446	0.1722	0.1206	0.1215
			4	3+	32	0.0825	0.0769	0.2912	0.3256	0.1480	0.1495
7	PP43	Wandered Away	1	No	1616	1.0000	0.9795	0.8940	0.8383	0.9140	0.9120
			2	Yes	156	0.0000	0.0205	0.1060	0.1617	0.0860	0.0880
8	PP44	Verbal Outbursts	1	No	1337	1.0000	1.0000	**0.0024**	0.9371	0.7487	0.7549
			2	Yes	434	0.0000	0.0000	**0.9976**	0.0629	0.2513	0.2451
9	PP45	Physical Threats	1	No	1592	1.0000	1.0000	0.6582	0.9443	0.9019	0.8989
			2	Threatening	98	0.0000	0.0000	0.2332	0.0000	0.0536	0.0553
			3	Physical	81	0.0000	0.0000	0.1086	0.0557	0.0445	0.0457
10	PP51_RC	Difficulty Sleeping	1	Slight difficulty	236	0.7224	0.6092	0.3922	0.4121	0.5086	0.5256
			2	Greater difficulty	104	0.2158	0.2198	0.3385	0.2137	0.2446	0.2316
			3	Excessive sleep	109	0.0618	0.1710	0.2693	0.3741	0.2468	0.2428
11	MMSE_GATE	Overall MMSE Gatekeeper	1	No answers	492	0.0487	0.0000	0.0293	0.6361	0.2384	0.2579
			2	Any answer	1416	0.9513	1.0000	0.9707	0.3639	0.7616	0.7421
12	Orientation_RC	Sum of Orientation Variables RECODE	1	0	105	**0.0000**	0.0000	0.0000	**0.2241**	0.0612	0.0742
			2	1-5	677	**0.0000**	0.3755	0.6588	**0.7759**	0.4695	0.4781
			3	6+	634	**1.0000**	0.6245	0.3412	**0.0000**	0.4693	0.4477
13	SP11B	MMSE -- Name 3 Objects	1	0	67	0.0050	0.0000	0.0000	0.1375	0.0388	0.0451
			2	1	64	0.0174	0.0000	0.0000	0.1235	0.0377	0.0431
			3	2	125	0.0265	0.0016	0.0750	0.1999	0.0790	0.0842
			4	3	1229	0.9512	0.9984	0.9250	0.5391	0.8446	0.8276
14	SP16B	MMSE -- World	1	0	203	0.0029	0.0275	0.0000	0.4241	0.1238	0.1374
			2	1	124	0.0353	0.1301	0.0127	0.1153	0.0771	0.0840
			3	2	185	0.0611	0.1129	0.1485	0.1366	0.1164	0.1253
			4	3	157	0.0615	0.2040	0.0642	0.0809	0.1056	0.1063
			5	4	86	0.0553	0.0741	0.0030	0.0951	0.0587	0.0582
			6	5	722	0.7839	0.4514	0.7717	0.1479	0.5184	0.4888
15	SP18B	MMSE -- Ask for 3 Objects	1	0	999	0.3853	0.6328	0.7572	0.8545	0.6686	0.6750
			2	1	255	0.2812	0.1935	0.1763	0.0987	0.1828	0.1723
			3	2	140	0.1675	0.1275	0.0666	0.0380	0.0973	0.0946
			4	3	86	0.1660	0.0461	0.0000	0.0088	0.0513	0.0581
16	Language	Sum of Language Variables	1	0	26	0.0000	0.0000	0.0000	0.0578	0.0158	0.0184
			2	1	14	0.0000	0.0000	0.0000	0.0311	0.0085	0.0099
			3	2	33	0.0000	0.0000	0.0000	0.0733	0.0200	0.0233
			4	3	24	0.0000	0.0000	0.0000	0.0533	0.0146	0.0169
			5	4	59	0.0000	0.0000	0.0000	0.1444	0.0394	0.0417
			6	5	79	0.0000	0.0466	0.0172	0.1305	0.0523	0.0558
			7	6	173	0.0848	0.0507	0.1182	0.1857	0.1111	0.1222
			8	7	394	0.2452	0.2942	0.4036	0.2138	0.2874	0.2782
			9	8	614	0.6700	0.6086	0.4610	0.1101	0.4509	0.4336
17	SP41B	MMSE -- Intersecting Pentagons-MMS	1	Wrong	787	0.1428	0.5363	0.3605	**1.0000**	0.5345	0.5481
			2	Right	649	0.8572	0.4637	0.6395	**0.0000**	0.4655	0.4519
18	NN01	Patient Trouble with Chores	1	None	305	**0.6966**	0.2060	0.0178	**0.0000**	0.1795	0.1678
			2	Some	469	**0.3034**	0.5481	0.3533	**0.0000**	0.2665	0.2580
			3	A lot	1044	**0.0000**	0.2459	0.6289	**1.0000**	0.5540	0.5743

19 NN02	Patient Trouble Handling Money	1 None	355	**0.7270**	0.3014	0.0003	**0.0000**	0.2036	0.1955
		2 Some	430	**0.2730**	0.4977	0.3791	**0.0000**	0.2548	0.2368
		3 A lot	1031	**0.0000**	0.2009	0.6206	**1.0000**	0.5416	0.5677
20 NN03	Patient Trouble Remembering Lists	1 None	45	**0.1520**	0.0189	0.0000	0.0016	0.0327	0.0248
		2 Some	256	**0.5386**	0.1996	0.0141	0.0000	0.1485	0.1408
		3 A lot	1517	**0.3093**	0.7815	0.9859	0.9984	0.8188	0.8344
21 NN04	Patient Trouble Around House	1 None	1110	1.0000	1.0000	0.7811	**0.0000**	0.5987	0.6109
		2 Some	360	0.0000	0.0000	0.2189	**0.4752**	0.2170	0.1981
		3 A lot	347	0.0000	0.0000	0.0000	**0.5248**	0.1843	0.1910
22 NN05	Patient Trouble Around Neighborhood	1 None	605	**0.9160**	**0.6805**	0.0484	**0.0000**	0.3392	0.3337
		2 Some	377	**0.0840**	**0.3195**	0.5798	**0.0000**	0.2239	0.2079
		3 A lot	831	**0.0000**	**0.0000**	0.3717	**1.0000**	0.4369	0.4584
23 NN06	Patient Trouble Recognizing Place	1 None	1072	**1.0000**	**1.0000**	0.5397	**0.0947**	0.5763	0.5906
		2 Some	311	**0.0000**	**0.0000**	0.4489	**0.2220**	0.1809	0.1713
		3 A lot	432	**0.0000**	**0.0000**	0.0114	**0.6834**	0.2428	0.2380
24 NN07	Patient Trouble Remembering Things	1 None	55	**0.1248**	0.0328	0.0000	0.0090	0.0336	0.0303
		2 Some	359	**0.6520**	0.3455	0.0764	0.0000	0.2178	0.1975
		3 A lot	1404	**0.2232**	0.6217	0.9236	0.9910	0.7487	0.7723
25 NN08	Patient Dwells in the Past	1 None	1051	0.9080	0.7256	**0.0170**	0.7013	0.5879	0.5803
		2 Some	368	0.0756	0.2744	**0.2834**	0.1717	0.2044	0.2032
		3 A lot	392	0.0165	0.0000	**0.6996**	0.1270	0.2078	0.2165
26 NN09	Patient Eating	1 Eats cleanly	1258	1.0000	1.0000	0.8492	0.2260	0.6938	0.6946
		2 Eats messily	385	0.0000	0.0000	0.1488	0.5222	0.2174	0.2126
		3 Eats only solids	44	0.0000	0.0000	0.0019	0.0647	0.0232	0.0243
		4 Has to be fed	124	0.0000	0.0000	0.0000	0.1871	0.0656	0.0685
27 NN10	Patient Dressing	1 Dress w/o help	815	**1.0000**	0.8652	0.3387	**0.0000**	0.4653	0.4500
		2 Misses details	407	**0.0000**	0.1348	0.5737	**0.1625**	0.2207	0.2247
		3 Gets mixed up	213	**0.0000**	0.0000	0.0876	**0.2689**	0.1144	0.1176
		4 Unable to dress	376	**0.0000**	0.0000	0.0000	**0.5686**	0.1995	0.2076
28 NN11	Patient Bladder and Bowel Control	1 Never wets bed	848	0.8137	0.8477	0.5615	**0.0000**	0.4783	0.4685
		2 Occasionally wets	436	0.1817	0.1523	0.3095	**0.2659**	0.2336	0.2409
		3 Frequently wets	255	0.0046	0.0000	0.1290	**0.3267**	0.1451	0.1409
		4 Incontinent	271	0.0000	0.0000	0.0000	**0.4073**	0.1430	0.1497
29 NN12	Increased Rigidity	1 No	1007	0.6717	0.7734	**0.0000**	0.7493	0.5693	0.5670
		2 Yes	769	0.3283	0.2266	**1.0000**	0.2507	0.4307	0.4330
30 NN13	Increased Egocentricity	1 No	1117	0.9451	0.8624	**0.0000**	0.7851	0.6519	0.6289
		2 Yes	659	0.0549	0.1376	**1.0000**	0.2149	0.3481	0.3711
31 NN14	Impairment of Regard for Feelings of Others	1 No	1251	1.0000	1.0000	**0.0249**	0.7925	0.7029	0.7040
		2 Yes	526	0.0000	0.0000	**0.9751**	0.2075	0.2971	0.2960
32 NN15	Coarsening of Affect	1 No	1207	0.8693	0.8872	**0.0000**	0.9002	0.6851	0.6789
		2 Yes	571	0.1307	0.1128	**1.0000**	0.0998	0.3149	0.3211
33 NN16	Impairment of Emotional Control	1 No	1086	0.8289	0.7652	**0.0000**	0.8645	0.6359	0.6108
		2 Yes	692	0.1711	0.2348	**1.0000**	0.1355	0.3641	0.3892
34 NN17	Hilarity in Inappropriate Situations	1 No	1466	0.9891	1.0000	0.4383	0.9103	0.8376	0.8250
		2 Yes	311	0.0109	0.0000	0.5617	0.0897	0.1624	0.1750
35 NN18	Diminished Emotional Responsiveness	1 No	1046	**1.0000**	0.8116	0.1588	0.4840	0.5793	0.5890
		2 Yes	730	**0.0000**	0.1884	0.8412	0.5160	0.4207	0.4110
36 NN19	Sexual Misdemeanor	1 No	1463	0.9136	0.9764	**0.3496**	0.9458	0.8106	0.8275
		2 Yes	305	0.0864	0.0236	**0.6504**	0.0542	0.1894	0.1725
37 NN20	Hobbies Relinquished	1 No	578	**0.8278**	0.3626	0.1190	0.1578	0.3167	0.3256
		2 Yes	1197	**0.1722**	0.6374	0.8810	0.8422	0.6833	0.6744
38 NN21	Diminished Initiative/Growing Apathy	1 No	508	0.7064	0.3907	0.0000	0.1856	0.2846	0.2857
		2 Yes	1270	0.2936	0.6093	1.0000	0.8144	0.7154	0.7143
39 NN22	Purposeless Hyperactivity	1 No	1374	0.9111	0.9994	0.3174	0.8514	0.7749	0.7750
		2 Yes	399	0.0889	0.0006	0.6826	0.1486	0.2251	0.2250
40 RR01	Needs Reminders	1 No	133	**0.3871**	0.0273	0.0000	0.0096	0.0789	0.0748
		2 Occasionally	131	**0.3753**	0.0942	0.0000	0.0033	0.0905	0.0737
		3 Frequently	1513	**0.2376**	0.8786	1.0000	0.9871	0.8307	0.8514
41 RR02	Needs Help to Remember	1 No	68	**0.2221**	0.0076	0.0029	0.0000	0.0422	0.0382
		2 Occasionally	144	**0.3318**	0.1447	0.0000	0.0034	0.0950	0.0809
		3 Frequently	1567	**0.4461**	0.8478	0.9971	0.9966	0.8629	0.8808
42 RR03	Needs Help Finding Things	1 No	138	0.3248	0.1058	0.0000	0.0028	0.0841	0.0776
		2 Yes	1641	0.6752	0.8942	1.0000	0.9972	0.9159	0.9224
43 RR04	Needs Household Chores Done	1 No	469	**1.0000**	0.4506	0.0000	0.0000	0.2859	0.2636
		2 Yes	1310	**0.0000**	0.5494	1.0000	1.0000	0.7141	0.7364
44 RR05	Needs Watching When Awake	1 No	987	**1.0000**	**1.0000**	0.6305	**0.0000**	0.5615	0.5533
		2 Yes	797	**0.0000**	**0.0000**	0.3695	**1.0000**	0.4385	0.4467
45 RR06	Needs to be Escorted When Outside	1 No	709	**1.0000**	**0.9862**	0.1076	**0.0000**	0.4382	0.3970
		2 Yes	1077	**0.0000**	**0.0138**	0.8924	**1.0000**	0.5618	0.6030
46 RR07	Needs to be Accompanied Bathing/Eating	1 No	1027	**1.0000**	**1.0000**	0.9130	**0.0000**	0.6257	0.5754
		2 Yes	758	**0.0000**	**0.0000**	0.0870	**1.0000**	0.3743	0.4246
47 RR08	Needs to be Dressed/Washed/Groomed	1 No	1180	1.0000	1.0000	1.0000	**0.0000**	0.6453	0.6625
		2 Yes	601	0.0000	0.0000	0.0000	**1.0000**	0.3547	0.3375
48 RR09	Needs to be Taken to Toilet	1 No	1291	1.0000	1.0000	1.0000	**0.1454**	0.6973	0.7253
		2 Yes	489	0.0000	0.0000	0.0000	**0.8546**	0.3027	0.2747

49 RR10	Needs to be Fed	1 No	1624	1.0000	1.0000	1.0000	0.7631	0.9160	0.9118
		2 Yes	157	0.0000	0.0000	0.0000	0.2369	0.0840	0.0882
50 RR11	Needs to be Turned/Moved/Transferred	1 No	1639	1.0000	1.0000	1.0000	0.7769	0.9208	0.9198
		2 Yes	143	0.0000	0.0000	0.0000	0.2231	0.0792	0.0802
51 RR12	Needs to Wear Diaper/Catheter	1 No	1269	1.0000	1.0000	1.0000	**0.1351**	0.6931	0.7121
		2 Yes	513	0.0000	0.0000	0.0000	**0.8649**	0.3069	0.2879
52 RR13	Needs to be Tube Fed	1 No	1762	1.0000	1.0000	1.0000	0.9684	0.9888	0.9893
		2 Yes	19	0.0000	0.0000	0.0000	0.0316	0.0112	0.0107
53 RR15	Equivalent Institutional Care	1 Limited home	520	**1.0000**	0.6282	**0.0000**	**0.0000**	0.3266	0.2936
		2 Adult home	702	**0.0000**	0.3718	**1.0000**	**0.1681**	0.3774	0.3964
		3 Full-time care	549	**0.0000**	0.0000	**0.0000**	**0.8319**	0.2960	0.3100
54 CC08_A_RC	Living Status RECODE	1 Home	1309	0.9703	0.9134	0.9358	0.2660	0.7002	0.7080
		2 Retirement home	39	0.0000	0.0547	0.0372	0.0045	0.0231	0.0211
		3 Nursing home	284	0.0000	0.0000	0.0000	0.4611	0.1628	0.1536
		4 Assist liv. facil.	187	0.0283	0.0266	0.0000	0.2506	0.0999	0.1011
		5 Other	30	0.0014	0.0053	0.0270	0.0179	0.0140	0.0162
55 INST_LOS_RC	Years Since Entering LTC Facility RECODE	1 0	130	**0.1475**	**0.8775**	0.7361	0.1381	0.3624	0.3394
		2 1	75	**0.0330**	**0.0000**	0.1377	0.2549	0.1838	0.1958
		3 2	46	**0.4131**	**0.0000**	0.0875	0.1449	0.1238	0.1201
		4 3	39	**0.0000**	**0.0260**	0.0298	0.1502	0.1023	0.1018
		5 4	34	**0.4064**	**0.0000**	0.0090	0.0980	0.0804	0.0888
		6 5+	59	**0.0000**	**0.0965**	0.0000	0.2140	0.1474	0.1540
56 FF10	Adequate Sight?	1 No	37	0.0133	0.0544	0.0000	0.1824	0.0681	0.0705
		2 Yes	488	0.9867	0.9456	1.0000	0.8176	0.9319	0.9295
57 FF11	Adequate Hearing?	1 No	50	0.0000	0.0654	0.0000	0.2836	0.0956	0.0954
		2 Yes	474	1.0000	0.9346	1.0000	0.7164	0.9044	0.9046
58 EF07	Admission to Hospital	1 No	1399	0.9018	0.9615	0.8095	0.8058	0.8569	0.8557
		2 Yes	236	0.0982	0.0385	0.1905	0.1942	0.1431	0.1443
59 EF08	Treatment	1 No	1350	0.8788	0.9015	0.8805	0.7319	0.8280	0.8262
		2 Yes	284	0.1212	0.0985	0.1195	0.2681	0.1720	0.1738
60 KK16	Had Seizure?	1 No	1651	0.9858	1.0000	0.9682	0.9344	0.9673	0.9666
		2 Yes	57	0.0142	0.0000	0.0318	0.0656	0.0327	0.0334
61 DELUSION	Delusions	1 No	1177	0.9560	0.9570	**0.0769**	0.6577	0.6505	0.6631
		2 Yes	598	0.0440	0.0430	**0.9231**	0.3423	0.3495	0.3369
62 HALLUCIN	Hallucinations	1 No	1557	1.0000	0.9726	0.7422	0.8293	0.8748	0.8787
		2 Yes	215	0.0000	0.0274	0.2578	0.1707	0.1252	0.1213
63 ILLUSION	Illusions	1 No	1684	1.0000	1.0000	0.9152	0.9318	0.9568	0.9579
		2 Yes	74	0.0000	0.0000	0.0848	0.0682	0.0432	0.0421
64 Beer_Wk_RC	Beer/Week: 0 - 99 RECODE	1 0	1140	0.9104	1.0000	0.8994	1.0000	0.9654	0.9661
		2 1-7	38	0.0801	0.0000	0.1006	0.0000	0.0333	0.0322
		3 8+	2	0.0095	0.0000	0.0000	0.0000	0.0013	0.0017
65 Wine_Wk_RC	Wine/Week: 0 - 99 RECODE	1 0	979	0.8806	0.6303	0.6963	0.9748	0.8256	0.8269
		2 1-7	183	0.1175	0.3051	0.3037	0.0252	0.1601	0.1546
		3 8+	22	0.0019	0.0646	0.0000	0.0000	0.0143	0.0186
66 Liquor_Wk_RC	Hard liquor/Week: 0 - 99 RECODE	1 0	1124	0.8994	0.9250	0.9210	1.0000	0.9524	0.9509
		2 1-7	49	0.0922	0.0479	0.0749	0.0000	0.0397	0.0415
		3 8+	9	0.0084	0.0270	0.0041	0.0000	0.0079	0.0076
67 EPSXX	Extrapyramidal symptoms	1 None	536	0.7506	0.5132	0.4321	**0.0000**	0.4044	0.3730
		2 Mild	517	0.2494	0.4868	0.4970	**0.1291**	0.3368	0.3598
		3 Moderate	384	0.0000	0.0000	0.0709	**0.8709**	0.2588	0.2672
68 Tremor	Tremor	1 No	1418	1.0000	0.9939	1.0000	0.7773	0.9334	0.9403
		2 Yes	90	0.0000	0.0061	0.0000	0.2227	0.0666	0.0597
69 Bradykinesia	Bradykinesia	1 No	1334	1.0000	1.0000	0.9965	0.6820	0.9111	0.9106
		2 Yes	131	0.0000	0.0000	0.0035	0.3180	0.0889	0.0894
70 Gait	Gait	1 No	1313	1.0000	1.0000	1.0000	0.6182	0.8940	0.8993
		2 Yes	147	0.0000	0.0000	0.0000	0.3818	0.1060	0.1007
71 Myoclonus	Myoclonus	1 No	1483	1.0000	0.9966	0.9850	0.9564	0.9828	0.9789
		2 Yes	32	0.0000	0.0034	0.0150	0.0436	0.0172	0.0211
72 Rigidity	Rigidity	1 No	1332	1.0000	1.0000	1.0000	0.5821	0.8795	0.8810
		2 Yes	180	0.0000	0.0000	0.0000	0.4179	0.1205	0.1190
73 AGITATION	Agitation	1 No	1073	0.9471	0.9660	**0.0000**	0.6534	0.6311	0.6048
		2 Yes	701	0.0529	0.0340	**1.0000**	0.3466	0.3689	0.3952
74 SAD	Sadness/Depression	1 No	1091	0.7964	0.6969	**0.0985**	0.7699	0.6030	0.6157
		2 Yes	681	0.2036	0.3031	**0.9015**	0.2301	0.3970	0.3843
75 DEP_FREQ_RC	Depression Frequency	1 Occasionally	323	0.6621	0.7906	**0.0012**	0.6032	0.4873	0.4843
		2 Some of the time	220	0.3062	0.2094	**0.3894**	0.3431	0.3186	0.3298
		3 Most of the time	96	0.0286	0.0000	**0.4644**	0.0409	0.1487	0.1439
		4 All of the time	28	0.0031	0.0000	**0.1449**	0.0127	0.0454	0.0420
76 AP	Appetite Problems	1 No	1332	0.9426	0.8426	0.5230	0.7248	0.7459	0.7534
		2 Yes	436	0.0574	0.1574	0.4770	0.2752	0.2541	0.2466
77 DL_Gate	Lewy Bodies Gatekeeper	1 No answers	318	0.2703	0.3211	0.3039	0.0377	0.2088	0.1658
		2 Any answer	1600	0.7297	0.6789	0.6961	0.9623	0.7912	0.8342
78 DL02	Fluctuating Cognition (Lewy)	1 No	1223	0.9541	0.8877	0.6983	0.7060	0.7892	0.7780
		2 Yes	349	0.0459	0.1123	0.3017	0.2940	0.2108	0.2220
79 DL03	Visual Hallucinations (Lewy)	1 No	1445	1.0000	1.0000	0.9098	0.8228	0.9143	0.9128
		2 Yes	138	0.0000	0.0000	0.0902	0.1772	0.0857	0.0872

		External Variables							
1 SP51_RC	MMSE Score (range 0-30) RECODE	1 0-15	366	**0.0000**	0.0062	0.0568	**0.7324**	0.2151	0.2585
		2 16-23	686	**0.1922**	0.7202	0.8646	**0.2676**	0.5147	0.4845
		3 24+	364	**0.8078**	0.2736	0.0786	**0.0000**	0.2702	0.2571
2 NNTOT_RC	BDRS Score RECODE	1 0.0-5.0	262	**0.6128**	**0.2033**	**0.0000**	**0.0000**	0.1572	0.1521
		2 5.5-10.0	491	**0.3872**	**0.7967**	**0.0867**	**0.0000**	0.2810	0.2850
		3 10.5-15.0	543	**0.0000**	**0.0000**	**0.8674**	**0.3619**	0.3267	0.3151
		4 15.5-28.0	427	**0.0000**	**0.0000**	**0.0459**	**0.6381**	0.2351	0.2478
3 RR14	Dependence Scale Score	1 Level 0	26	**0.0742**	**0.0000**	**0.0000**	**0.0028**	0.0141	0.0146
		2 Level 1	49	**0.1328**	**0.0435**	**0.0000**	**0.0018**	0.0345	0.0276
		3 Level 2	531	**0.7898**	**0.6996**	**0.0756**	**0.0000**	0.3241	0.2990
		4 Level 3	439	**0.0000**	**0.2569**	**0.8123**	**0.0000**	0.2472	0.2472
		5 Level 4	219	**0.0000**	**0.0000**	**0.0886**	**0.2726**	0.1172	0.1233
		6 Level 5	512	**0.0032**	**0.0000**	**0.0236**	**0.7228**	0.2628	0.2883
4 Alcohol_Wk_RC	Alcoholic Drinks/Week RECODE	1 0	929	0.7797	0.6235	0.6172	0.9708	0.7911	0.7880
		2 1-7	210	0.1419	0.2742	0.3775	0.0292	0.1746	0.1781
		3 8+	40	0.0784	0.1023	0.0054	0.0000	0.0343	0.0339
5 QQ01_RC	CDR Rating	1 0.5=Questionable	38	**0.0762**	**0.0221**	0.0085	**0.0000**	0.0218	0.0214
		2 1=Mild	1003	**0.9238**	**0.9746**	0.8459	**0.0000**	0.6059	0.5660
		3 2=Moderate	476	**0.0000**	**0.0018**	0.1455	**0.6266**	0.2456	0.2686
		4 3=Severe	181	**0.0000**	**0.0000**	0.0000	**0.2662**	0.0900	0.1021
		5 4=Profound	60	**0.0000**	**0.0000**	0.0000	**0.0882**	0.0298	0.0339
		6 5=Terminal	14	**0.0000**	**0.0016**	0.0000	**0.0190**	0.0068	0.0079
6 PSYCHSX	Psychiatric Symptoms	1 No	1093	0.9571	0.9242	**0.0010**	0.5842	0.5997	0.6158
		2 Yes	682	0.0429	0.0758	**0.9990**	0.4158	0.4003	0.3842

Notes: j denotes variable number; l indexes response levels; N_{jl} is the number of respondents for (j,l); λ_1–λ_4 are the hidden outcome probabilities for the K=4 subtypes; $E(\pi^{jl}{}_i)$ is the model-based mean response rate; Obs. Rate is the observed mean response rate. Boldface red font indicates $H_{jk} > 0.5$.

Table A.4. Cumulative Transition Matrices (V_t) at 21 Semiannual Examinations, Predictors 2 Study Cohort

Time	Subtype	Subtype (*k*)				Time	Subtype	Subtype (*k*)			
t	*k*	1	2	3	4	*t*	*k*	1	2	3	4
0	1	**1.0000**	**-**	**-**	**-**	10	1	**0.2121**	**0.4304**	**0.2740**	**0.0836**
	2	**-**	**1.0000**	**-**	**-**		2	**-**	**0.0000**	**0.0810**	**0.9190**
	3	**-**	**-**	**1.0000**	**-**		3	**-**	**-**	**0.5542**	**0.4458**
	4	**-**	**-**	**-**	**1.0000**		4	**-**	**-**	**-**	**1.0000**
1	1	1.0000	0.0000	0.0000	0.0000	11	1	0.0960	0.5464	0.2740	0.0836
	2	-	0.9098	0.0698	0.0204		2	-	0.0000	0.0810	0.9190
	3	-	-	0.9946	0.0054		3	-	-	0.5542	0.4458
	4	-	-	-	1.0000		4	-	-	-	1.0000
2	1	1.0000	0.0000	0.0000	0.0000	12	1	0.0101	0.5399	0.2246	0.2255
	2	-	0.7160	0.1432	0.1407		2	-	0.0000	0.0391	0.9609
	3	-	-	0.9796	0.0204		3	-	-	0.2672	0.7328
	4	-	-	-	1.0000		4	-	-	-	1.0000
3	1	0.9757	0.0000	0.0243	0.0000	13	1	0.0101	0.4849	0.2598	0.2453
	2	-	0.5133	0.1432	0.3435		2	-	0.0000	0.0380	0.9620
	3	-	-	0.9796	0.0204		3	-	-	0.2601	0.7399
	4	-	-	-	1.0000		4	-	-	-	1.0000
4	1	0.9757	0.0000	0.0243	0.0000	14	1	0.0000	0.4949	0.2573	0.2478
	2	-	0.3636	0.1432	0.4931		2	-	0.0000	0.0377	0.9623
	3	-	-	0.9796	0.0204		3	-	-	0.2575	0.7425
	4	-	-	-	1.0000		4	-	-	-	1.0000
5	1	**0.5796**	**0.3961**	**0.0243**	**0.0000**	15	1	**0.0000**	**0.3379**	**0.1896**	**0.4725**
	2	**-**	**0.0000**	**0.1432**	**0.8568**		2	**-**	**0.0000**	**0.0225**	**0.9775**
	3	**-**	**-**	**0.9796**	**0.0204**		3	**-**	**-**	**0.1538**	**0.8462**
	4	**-**	**-**	**-**	**1.0000**		4	**-**	**-**	**-**	**1.0000**
6	1	0.4236	0.5412	0.0334	0.0018	16	1	0.0000	0.3379	0.1896	0.4725
	2	-	0.0000	0.1324	0.8676		2	-	0.0000	0.0225	0.9775
	3	-	-	0.9056	0.0944		3	-	-	0.1538	0.8462
	4	-	-	-	1.0000		4	-	-	-	1.0000
7	1	0.2451	0.6591	0.0930	0.0029	17	1	0.0000	0.3379	0.1896	0.4725
	2	-	0.0000	0.1281	0.8719		2	-	0.0000	0.0225	0.9775
	3	-	-	0.8765	0.1235		3	-	-	0.1538	0.8462
	4	-	-	-	1.0000		4	-	-	-	1.0000
8	1	0.2451	0.6503	0.0826	0.0221	18	1	0.0000	0.3379	0.1896	0.4725
	2	-	0.0000	0.1017	0.8983		2	-	0.0000	0.0225	0.9775
	3	-	-	0.6957	0.3043		3	-	-	0.1538	0.8462
	4	-	-	-	1.0000		4	-	-	-	1.0000
9	1	0.2451	0.4169	0.3017	0.0364	19	1	0.0000	0.3379	0.0841	0.5780
	2	-	0.0000	0.0961	0.9039		2	-	0.0000	0.0100	0.9900
	3	-	-	0.6570	0.3430		3	-	-	0.0682	0.9318
	4	-	-	-	1.0000		4	-	-	-	1.0000
10	1	**0.2121**	**0.4304**	**0.2740**	**0.0836**	20	1	**0.0000**	**0.3379**	**0.0841**	**0.5780**
	2	**-**	**0.0000**	**0.0810**	**0.9190**		2	**-**	**0.0000**	**0.0100**	**0.9900**
	3	**-**	**-**	**0.5542**	**0.4458**		3	**-**	**-**	**0.0682**	**0.9318**
	4	**-**	**-**	**-**	**1.0000**		4	**-**	**-**	**-**	**1.0000**

Notes: Time *t* runs from 0 to 20 half-years (0–10 years); all $\mathbf{V}_t$ are upper triangular 4x4 matrices; $\mathbf{V}_0$ is the identity matrix; $\mathbf{V}_{10}$ is repeated at top of right-hand column. Boldface red font highlights every 5th matrix. The 4 rows of the transition matrices are in one-to-one correspondence with the 4 pure-subtype trajectories shown in Table A.5, obtainable by re-sorting the rows of the 21 matrices so that *t* changes more rapidly than *k*.

Table A.5. Pure-Subtype Trajectories for Four Subtypes at 21 Semiannual Examinations, Predictors 2 Study Cohort

Subtype	Time	Subtype (*k*)				Subtype	Time	Subtype (*k*)			
k	*t*	1	2	3	4	*k*	*t*	1	2	3	4
1	0	**1.0000**	**-**	**-**	**-**	3	0	**-**	**-**	**1.0000**	**-**
1	1	1.0000	0.0000	0.0000	0.0000	3	1	-	-	0.9946	0.0054
1	2	1.0000	0.0000	0.0000	0.0000	3	2	-	-	0.9796	0.0204
1	3	0.9757	0.0000	0.0243	0.0000	3	3	-	-	0.9796	0.0204
1	4	0.9757	0.0000	0.0243	0.0000	3	4	-	-	0.9796	0.0204
1	5	**0.5796**	**0.3961**	**0.0243**	**0.0000**	3	5	**-**	**-**	**0.9796**	**0.0204**
1	6	0.4236	0.5412	0.0334	0.0018	3	6	-	-	0.9056	0.0944
1	7	0.2451	0.6591	0.0930	0.0029	3	7	-	-	0.8765	0.1235
1	8	0.2451	0.6503	0.0826	0.0221	3	8	-	-	0.6957	0.3043
1	9	0.2451	0.4169	0.3017	0.0364	3	9	-	-	0.6570	0.3430
1	10	**0.2121**	**0.4304**	**0.2740**	**0.0836**	3	10	**-**	**-**	**0.5542**	**0.4458**
1	11	0.0960	0.5464	0.2740	0.0836	3	11	-	-	0.5542	0.4458
1	12	0.0101	0.5399	0.2246	0.2255	3	12	-	-	0.2672	0.7328
1	13	0.0101	0.4849	0.2598	0.2453	3	13	-	-	0.2601	0.7399
1	14	0.0000	0.4949	0.2573	0.2478	3	14	-	-	0.2575	0.7425
1	15	**0.0000**	**0.3379**	**0.1896**	**0.4725**	3	15	**-**	**-**	**0.1538**	**0.8462**
1	16	0.0000	0.3379	0.1896	0.4725	3	16	-	-	0.1538	0.8462
1	17	0.0000	0.3379	0.1896	0.4725	3	17	-	-	0.1538	0.8462
1	18	0.0000	0.3379	0.1896	0.4725	3	18	-	-	0.1538	0.8462
1	19	0.0000	0.3379	0.0841	0.5780	3	19	-	-	0.0682	0.9318
1	20	**0.0000**	**0.3379**	**0.0841**	**0.5780**	3	20	**-**	**-**	**0.0682**	**0.9318**
2	0	**-**	**1.0000**	**-**	**-**	4	0	**-**	**-**	**-**	**1.0000**
2	1	-	0.9098	0.0698	0.0204	4	1	-	-	-	1.0000
2	2	-	0.7160	0.1432	0.1407	4	2	-	-	-	1.0000
2	3	-	0.5133	0.1432	0.3435	4	3	-	-	-	1.0000
2	4	-	0.3636	0.1432	0.4931	4	4	-	-	-	1.0000
2	5	**-**	**0.0000**	**0.1432**	**0.8568**	4	5	**-**	**-**	**-**	**1.0000**
2	6	-	0.0000	0.1324	0.8676	4	6	-	-	-	1.0000
2	7	-	0.0000	0.1281	0.8719	4	7	-	-	-	1.0000
2	8	-	0.0000	0.1017	0.8983	4	8	-	-	-	1.0000
2	9	-	0.0000	0.0961	0.9039	4	9	-	-	-	1.0000
2	10	**-**	**0.0000**	**0.0810**	**0.9190**	4	10	**-**	**-**	**-**	**1.0000**
2	11	-	0.0000	0.0810	0.9190	4	11	-	-	-	1.0000
2	12	-	0.0000	0.0391	0.9609	4	12	-	-	-	1.0000
2	13	-	0.0000	0.0380	0.9620	4	13	-	-	-	1.0000
2	14	-	0.0000	0.0377	0.9623	4	14	-	-	-	1.0000
2	15	**-**	**0.0000**	**0.0225**	**0.9775**	4	15	**-**	**-**	**-**	**1.0000**
2	16	-	0.0000	0.0225	0.9775	4	16	-	-	-	1.0000
2	17	-	0.0000	0.0225	0.9775	4	17	-	-	-	1.0000
2	18	-	0.0000	0.0225	0.9775	4	18	-	-	-	1.0000
2	19	-	0.0000	0.0100	0.9900	4	19	-	-	-	1.0000
2	20	**-**	**0.0000**	**0.0100**	**0.9900**	4	20	**-**	**-**	**-**	**1.0000**

Notes: The pure-subtype trajectories are sets of time-varying GoM scores that change from one examination to the next. Each pure-subtype has its own trajectory beginning with its unique unit value at time 0. Time t runs from 0 to 20 half-years (0–10 years). Boldface red font highlights every 5th set of GoM scores. The pure-subtype trajectories are in one-to-one correspondence with the transition matrices shown in Table A.4, obtainable by re-sorting the rows of the trajectories so that k changes more rapidly than t.

Table A.6. Age-Group at Intake, Sex, GoM Scores and Subgroups, and Sullivan and Conditional Life Expectancies for 229 Participants in the Predictors 2 Study Cohort

Subject (i)	Age Group	Sex (1=M 2=F)	GoM Sub-Group	g_{i1}	g_{i2}	g_{i3}	g_{i4}	TLE	NLE	DLE	Pr(Censored at Year 10)	Pr(No FTC at Intake)	Joint Prob. of No FTC at Intake and…: Death before FTC	Joint Prob. of No FTC at Intake and…: FTC before Death	Joint Prob. of No FTC at Intake and…: Death after FTC	Pr(FTC at Any Time)	Pr(FTC at Intake)	Joint Prob. of No FTC at Intake and FTC before Death	Cond. DFLE	Among those with No FTC at Intake…: Time to Death	Among those with No FTC at Intake…: Time to FTC	Among those with No FTC at Intake…: Total Time to Death	Cond. DLE	Cond. DLE_1	Cond. DLE_2
(Corresponding Column in Tables 3, S.1, and S.2)				—	—	—	—	Table 3 (2)	Table 3 (3)	Table 3 (4)	—	Table S.1 (2)	Table S.1 (3)	Table S.1 (4)	Table S.1 (5)	Table S.1 (6)	Table S.1 (7)	Table S.1 (8)	Table S.2 (2)	Table S.2 (3)	Table S.2 (4)	Table S.2 (5)	Table S.2 (6)	Table S.2 (7)	Table S.2 (8)
1	1	1	0	0.4842	0.2060	0.3097	0.0000	7.264	5.410	1.854	0.2872	1.0000	0.5246	0.4754	0.4754	0.4754	0.0000	0.4754	5.410	6.382	4.337	8.239	3.902	7.264	3.902
2	1	1	0	0.4024	0.4397	0.0239	0.1341	5.583	3.429	2.155	0.1579	0.8885	0.4996	0.3888	0.3888	0.5004	0.1115	0.3888	3.859	4.696	2.783	6.729	4.310	5.583	3.945
3	1	1	1	**0.8280**	0.0000	0.1720	0.0000	8.554	7.212	1.342	0.4764	1.0000	0.5643	0.4357	0.4357	0.4357	0.0000	0.4357	7.212	8.041	6.137	9.220	3.082	8.554	3.082
4	1	1	1	**0.8919**	0.0000	0.1081	0.0000	8.654	7.382	1.272	0.5014	1.0000	0.5756	0.4244	0.4244	0.4244	0.0000	0.4244	7.382	8.180	6.300	9.299	2.999	8.654	2.999
5	1	1	1	**0.5038**	0.3424	0.1537	0.0000	6.817	4.874	1.942	0.2504	1.0000	0.5291	0.4709	0.4709	0.4709	0.0000	0.4709	4.874	5.960	3.654	7.782	4.128	6.817	4.128
6	1	1	2	0.0000	**0.5283**	0.4717	0.0000	5.885	3.599	2.286	0.1318	1.0000	0.3990	0.6010	0.6010	0.6010	0.0000	0.6010	3.599	4.793	2.807	6.616	3.809	5.885	3.809
7	1	1	2	0.0346	**0.5084**	0.4570	0.0000	5.958	3.693	2.265	0.1390	1.0000	0.4096	0.5904	0.5904	0.5904	0.0000	0.5904	3.693	4.884	2.867	6.708	3.841	5.958	3.841
8	1	1	3	0.0000	0.4361	**0.5639**	0.0000	6.123	3.873	2.250	0.1468	1.0000	0.4099	0.5901	0.5901	0.5901	0.0000	0.5901	3.873	4.987	3.099	6.916	3.818	6.123	3.818
9	1	1	4	0.0000	0.3595	0.0000	**0.6405**	3.415	1.048	2.367	0.0429	0.4672	0.2807	0.1865	0.1865	0.7193	0.5328	0.1865	2.244	2.584	1.732	4.681	3.294	3.415	2.949
10	1	2	0	0.1717	0.4724	0.3559	0.0000	6.149	3.957	2.192	0.1631	1.0000	0.4451	0.5549	0.5549	0.5549	0.0000	0.5549	3.957	5.157	2.993	6.949	3.955	6.149	3.955
11	1	2	1	**1.0000**	0.0000	0.0000	0.0000	8.831	7.685	1.146	0.5462	1.0000	0.5940	0.4060	0.4060	0.4060	0.0000	0.4060	7.685	8.426	6.601	9.425	2.823	8.831	2.823
12	1	2	1	**0.6497**	0.3503	0.0000	0.0000	6.936	5.083	1.853	0.2800	1.0000	0.5597	0.4403	0.4403	0.4403	0.0000	0.4403	5.083	6.154	3.721	7.933	4.212	6.936	4.212
13	1	2	1	**0.8596**	0.1404	0.0000	0.0000	7.981	6.499	1.482	0.4193	1.0000	0.5952	0.4048	0.4048	0.4048	0.0000	0.4048	6.499	7.307	5.312	8.974	3.662	7.981	3.662
14	1	2	2	0.3614	**0.6386**	0.0000	0.0000	5.847	3.662	2.185	0.1581	1.0000	0.4601	0.5399	0.5399	0.5399	0.0000	0.5399	3.662	5.090	2.445	6.497	4.052	5.847	4.052
15	1	2	2	0.4260	**0.5740**	0.0000	0.0000	6.062	3.937	2.125	0.1800	1.0000	0.4863	0.5137	0.5137	0.5137	0.0000	0.5137	3.937	5.296	2.651	6.791	4.140	6.062	4.140
16	1	2	2	0.0000	**0.7538**	0.1336	0.1126	4.820	2.432	2.387	0.0802	0.9064	0.3466	0.5597	0.5597	0.6534	0.0936	0.5597	2.684	3.750	2.023	5.490	3.660	4.820	3.467
17	1	2	2	0.0000	**0.9480**	0.0520	0.0000	4.980	2.568	2.412	0.0798	1.0000	0.3018	0.6982	0.6982	0.6982	0.0000	0.6982	2.568	4.134	1.891	5.353	3.462	4.980	3.462
18	1	2	2	0.0000	**0.7939**	0.1291	0.0770	4.909	2.519	2.390	0.0822	0.9359	0.3399	0.5960	0.5960	0.6601	0.0641	0.5960	2.692	3.880	2.013	5.503	3.627	4.909	3.490
19	1	2	2	0.2609	**0.6852**	0.0000	0.0539	5.371	3.101	2.270	0.1232	0.9552	0.4252	0.5299	0.5299	0.5748	0.0448	0.5299	3.247	4.545	2.205	6.040	3.954	5.371	3.834
20	1	2	2	0.0000	**0.8941**	0.0000	0.1059	4.606	2.189	2.417	0.0685	0.9119	0.3049	0.6070	0.6070	0.6951	0.0881	0.6070	2.400	3.583	1.806	5.129	3.485	4.606	3.323
21	2	1	1	**0.5146**	0.0000	0.4854	0.0000	8.101	6.456	1.646	0.3699	1.0000	0.5039	0.4961	0.4961	0.4961	0.0000	0.4961	6.456	7.417	5.479	8.799	3.320	8.101	3.320
22	2	1	2	0.0000	**0.5755**	0.1695	0.2551	4.505	2.133	2.372	0.0742	0.7878	0.3685	0.4193	0.4193	0.6315	0.2122	0.4193	2.708	3.376	2.121	5.507	3.762	4.505	3.386
23	2	1	2	0.1259	**0.7445**	0.1296	0.0000	5.453	3.136	2.317	0.1139	1.0000	0.3852	0.6148	0.6148	0.6148	0.0000	0.6148	3.136	4.600	2.219	5.992	3.774	5.453	3.774
24	2	1	2	0.3689	**0.5491**	0.0819	0.0000	6.087	3.948	2.139	0.1765	1.0000	0.4781	0.5219	0.5219	0.5219	0.0000	0.5219	3.948	5.269	2.737	6.840	4.102	6.087	4.102
25	2	1	3	0.3735	0.0000	**0.6265**	0.0000	7.917	6.155	1.762	0.3295	1.0000	0.4745	0.5255	0.5255	0.5255	0.0000	0.5255	6.155	7.160	5.248	8.604	3.356	7.917	3.356
26	2	1	4	0.0000	0.1304	0.2259	**0.6438**	3.680	1.346	2.333	0.0565	0.4645	0.3433	0.1212	0.1212	0.6567	0.5355	0.1212	2.898	2.908	2.869	5.874	3.555	3.680	3.005
27	2	2	0	0.2793	0.3089	0.3363	0.0755	6.196	4.086	2.110	0.1859	0.9372	0.4931	0.4441	0.4441	0.5069	0.0628	0.4441	4.360	5.134	3.501	7.380	4.167	6.196	3.880
28	2	2	1	**0.6560**	0.0613	0.2827	0.0000	8.035	6.451	1.584	0.3880	1.0000	0.5443	0.4557	0.4557	0.4557	0.0000	0.4557	6.451	7.331	5.400	8.878	3.478	8.035	3.478
29	2	2	2	0.0000	**0.9689**	0.0000	0.0311	4.801	2.380	2.422	0.0730	0.9742	0.2919	0.6822	0.6822	0.7081	0.0258	0.6822	2.443	3.907	1.817	5.193	3.429	4.801	3.377
30	2	2	2	0.3226	**0.6774**	0.0000	0.0000	5.725	3.506	2.218	0.1461	1.0000	0.4434	0.5566	0.5566	0.5566	0.0000	0.5566	3.506	4.972	2.339	6.330	3.991	5.725	3.991
31	2	2	2	0.0000	**1.0000**	0.0000	0.0000	4.885	2.462	2.423	0.0749	1.0000	0.2852	0.7148	0.7148	0.7148	0.0000	0.7148	2.462	4.068	1.821	5.220	3.399	4.885	3.399
32	2	2	2	0.2971	**0.6657**	0.0371	0.0000	5.737	3.513	2.224	0.1449	1.0000	0.4405	0.5595	0.5595	0.5595	0.0000	0.5595	3.513	4.958	2.376	6.355	3.979	5.737	3.979
33	2	2	2	0.0000	**0.9165**	0.0835	0.0000	5.039	2.635	2.404	0.0829	1.0000	0.3114	0.6886	0.6886	0.6886	0.0000	0.6886	2.635	4.175	1.938	5.437	3.499	5.039	3.499
34	2	2	3	0.1490	0.2748	**0.5762**	0.0000	6.704	4.601	2.103	0.2007	1.0000	0.4519	0.5481	0.5481	0.5481	0.0000	0.5481	4.601	5.633	3.750	7.592	3.842	6.704	3.842
35	3	1	0	0.2947	0.1540	0.2774	0.2739	5.403	3.251	2.152	0.1518	0.7721	0.5084	0.2637	0.2637	0.4916	0.2279	0.2637	4.211	4.368	3.908	7.406	4.381	5.403	3.498
36	3	1	0	0.3123	0.2229	0.3710	0.0938	6.349	4.289	2.060	0.2035	0.9220	0.5077	0.4143	0.4143	0.4923	0.0780	0.4143	4.652	5.262	3.905	7.686	4.188	6.349	3.781
37	3	1	0	0.1846	0.4037	0.4117	0.0000	6.350	4.197	2.153	0.1785	1.0000	0.4548	0.5452	0.5452	0.5452	0.0000	0.5452	4.197	5.335	3.247	7.201	3.954	6.350	3.954
38	3	1	1	**0.9995**	0.0005	0.0000	0.0000	8.827	7.681	1.147	0.5457	1.0000	0.5941	0.4059	0.4059	0.4059	0.0000	0.4059	7.681	8.421	6.597	9.424	2.827	8.827	2.827
39	3	1	1	**0.6876**	0.0895	0.2229	0.0000	7.960	6.374	1.585	0.3859	1.0000	0.5552	0.4448	0.4448	0.4448	0.0000	0.4448	6.374	7.240	5.294	8.860	3.566	7.960	3.566
40	3	1	1	**0.7339**	0.2594	0.0067	0.0000	7.350	5.636	1.714	0.3321	1.0000	0.5785	0.4215	0.4215	0.4215	0.0000	0.4215	5.636	6.586	4.333	8.401	4.069	7.350	4.069
41	3	1	1	**1.0000**	0.0000	0.0000	0.0000	8.831	7.685	1.146	0.5462	1.0000	0.5940	0.4060	0.4060	0.4060	0.0000	0.4060	7.685	8.426	6.601	9.425	2.823	8.831	2.823
42	3	1	1	**1.0000**	0.0000	0.0000	0.0000	8.831	7.685	1.146	0.5462	1.0000	0.5940	0.4060	0.4060	0.4060	0.0000	0.4060	7.685	8.426	6.601	9.425	2.823	8.831	2.823
43	3	1	1	**0.6457**	0.0000	0.3543	0.0000	8.283	6.756	1.527	0.4115	1.0000	0.5300	0.4700	0.4700	0.4700	0.0000	0.4700	6.756	7.668	5.728	8.979	3.251	8.283	3.251
44	3	1	1	**1.0000**	0.0000	0.0000	0.0000	8.831	7.685	1.146	0.5462	1.0000	0.5940	0.4060	0.4060	0.4060	0.0000	0.4060	7.685	8.426	6.601	9.425	2.823	8.831	2.823
45	3	1	1	**0.7117**	0.2163	0.0720	0.0000	7.486	5.795	1.692	0.3421	1.0000	0.5725	0.4275	0.4275	0.4275	0.0000	0.4275	5.795	6.713	4.565	8.524	3.959	7.486	3.959
46	3	1	1	**0.8056**	0.0000	0.1944	0.0000	8.519	7.153	1.366	0.4680	1.0000	0.5602	0.4398	0.4398	0.4398	0.0000	0.4398	7.153	7.994	6.083	9.191	3.108	8.519	3.108
47	3	1	2	0.0000	**0.5303**	0.0000	0.4697	3.759	1.373	2.386	0.0500	0.6093	0.3090	0.3004	0.3004	0.6910	0.3907	0.3004	2.254	2.738	1.756	4.823	3.458	3.759	3.067
48	3	1	2	0.3314	**0.6492**	0.0194	0.0000	5.800	3.598	2.203	0.1522	1.0000	0.4514	0.5486	0.5486	0.5486	0.0000	0.5486	3.598	5.031	2.418	6.437	4.020	5.800	4.020
49	3	1	2	0.0447	**0.5453**	0.4101	0.0000	5.872	3.596	2.276	0.1343	1.0000	0.4066	0.5934	0.5934	0.5934	0.0000	0.5934	3.596	4.825	2.754	6.594	3.840	5.872	3.840
50	3	1	2	0.0000	**0.5469**	0.3073	0.1458	5.056	2.714	2.342	0.0961	0.8787	0.3953	0.4833	0.4833	0.6047	0.1213	0.4833	3.089	3.889	2.435	6.017	3.878	5.056	3.583
51	3	1	3	0.4798	0.0006	**0.5039**	0.0157	7.909	6.210	1.699	0.3487	0.9869	0.5052	0.4817	0.4817	0.4948	0.0131	0.4817	6.293	7.127	5.417	8.733	3.437	7.909	3.315
52	3	1	3	0.2540	0.1635	**0.5826**	0.0000	7.170	5.195	1.974	0.2487	1.0000	0.4716	0.5284	0.5284	0.5284	0.0000	0.5284	5.195	6.179	4.317	8.057	3.740	7.170	3.740
53	3	1	3	0.1800	0.1474	**0.5269**	0.1458	6.113	3.990	2.123	0.1790	0.8787	0.4894	0.3894	0.3894	0.5106	0.1213	0.3894	4.541	4.891	4.101	7.654	4.161	6.113	3.553
54	3	1	3	0.0000	0.4528	**0.5472**	0.0000	6.079	3.822	2.257	0.1439	1.0000	0.4083	0.5917	0.5917	0.5917	0.0000	0.5917	3.822	4.950	3.043	6.862	3.819	6.079	3.819

55	3	2	0	0.0000	0.2814	0.3028	0.4158	4.318	1.984	2.334	0.0759	0.6541	0.3983	0.2558	0.2558	0.6017	0.3459	0.2558	3.033	3.248	2.697	5.992	3.883	4.318	3.295
56	3	2	0	0.2550	0.3181	0.4269	0.0000	6.664	4.592	2.071	0.2090	1.0000	0.4753	0.5247	0.5247	0.5247	0.0000	0.5247	4.592	5.664	3.622	7.573	3.951	6.664	3.951
57	3	2	1	1.0000	0.0000	0.0000	0.0000	8.831	7.685	1.146	0.5462	1.0000	0.5940	0.4060	0.4060	0.4060	0.0000	0.4060	7.685	8.426	6.601	9.425	2.823	8.831	2.823
58	3	2	1	0.6122	0.0044	0.3834	0.0000	8.216	6.654	1.563	0.3986	1.0000	0.5245	0.4755	0.4755	0.4755	0.0000	0.4755	6.654	7.576	5.637	8.926	3.289	8.216	3.289
59	3	2	1	0.8571	0.0000	0.1429	0.0000	8.599	7.289	1.311	0.4876	1.0000	0.5695	0.4305	0.4305	0.4305	0.0000	0.4305	7.289	8.104	6.210	9.256	3.046	8.599	3.046
60	3	2	1	0.7746	0.2254	0.0000	0.0000	7.528	5.879	1.649	0.3564	1.0000	0.5856	0.4144	0.4144	0.4144	0.0000	0.4144	5.879	6.783	4.602	8.582	3.980	7.528	3.980
61	3	2	1	1.0000	0.0000	0.0000	0.0000	8.831	7.685	1.146	0.5462	1.0000	0.5940	0.4060	0.4060	0.4060	0.0000	0.4060	7.685	8.426	6.601	9.425	2.823	8.831	2.823
62	3	2	1	0.9890	0.0110	0.0000	0.0000	8.759	7.584	1.175	0.5350	1.0000	0.5950	0.4050	0.4050	0.4050	0.0000	0.4050	7.584	8.323	6.498	9.401	2.903	8.759	2.903
63	3	2	1	0.6821	0.3179	0.0000	0.0000	7.082	5.277	1.804	0.2982	1.0000	0.5676	0.4324	0.4324	0.4324	0.0000	0.4324	5.277	6.304	3.930	8.105	4.176	7.082	4.176
64	3	2	1	0.9036	0.0719	0.0246	0.0000	8.341	6.987	1.354	0.4680	1.0000	0.5931	0.4069	0.4069	0.4069	0.0000	0.4069	6.987	7.754	5.869	9.199	3.330	8.341	3.330
65	3	2	1	0.7555	0.2445	0.0000	0.0000	7.431	5.749	1.683	0.3436	1.0000	0.5825	0.4175	0.4175	0.4175	0.0000	0.4175	5.749	6.677	4.454	8.486	4.033	7.431	4.033
66	3	2	1	0.8981	0.0000	0.1019	0.0000	8.664	7.399	1.265	0.5038	1.0000	0.5767	0.4233	0.4233	0.4233	0.0000	0.4233	7.399	8.194	6.317	9.307	2.990	8.664	2.990
67	3	2	1	0.5535	0.1306	0.2867	0.0292	7.378	5.601	1.777	0.3126	0.9757	0.5434	0.4323	0.4323	0.4566	0.0243	0.4323	5.740	6.487	4.802	8.501	3.895	7.378	3.700
68	3	2	1	0.5165	0.1141	0.3269	0.0425	7.287	5.479	1.808	0.3011	0.9646	0.5387	0.4259	0.4259	0.4613	0.0354	0.4259	5.680	6.354	4.828	8.469	3.921	7.287	3.641
69	3	2	2	0.0000	0.5137	0.2621	0.2242	4.749	2.397	2.352	0.0852	0.8135	0.3914	0.4221	0.4221	0.6086	0.1865	0.4221	2.947	3.588	2.352	5.833	3.869	4.749	3.481
70	3	2	2	0.0000	0.8120	0.1880	0.0000	5.244	2.867	2.377	0.0941	1.0000	0.3408	0.6592	0.6592	0.6592	0.0000	0.6592	2.867	4.318	2.117	5.729	3.612	5.244	3.612
71	3	2	2	0.0000	0.6746	0.1767	0.1487	4.799	2.424	2.375	0.0819	0.8763	0.3631	0.5132	0.5132	0.6369	0.1237	0.5132	2.766	3.687	2.114	5.594	3.736	4.799	3.480
72	3	2	2	0.3414	0.6586	0.0000	0.0000	5.783	3.581	2.203	0.1518	1.0000	0.4516	0.5484	0.5484	0.5484	0.0000	0.5484	3.581	5.028	2.389	6.410	4.021	5.783	4.021
73	3	2	2	0.1073	0.7301	0.1626	0.0000	5.474	3.156	2.318	0.1140	1.0000	0.3846	0.6154	0.6154	0.6154	0.0000	0.6154	3.156	4.596	2.256	6.029	3.773	5.474	3.773
74	3	2	2	0.3426	0.5614	0.0960	0.0000	6.034	3.877	2.157	0.1702	1.0000	0.4703	0.5297	0.5297	0.5297	0.0000	0.5297	3.877	5.209	2.695	6.772	4.077	6.034	4.077
75	3	2	2	0.0000	0.7965	0.2035	0.0000	5.276	2.903	2.373	0.0959	1.0000	0.3448	0.6552	0.6552	0.6552	0.0000	0.6552	2.903	4.341	2.147	5.775	3.628	5.276	3.628
76	3	2	2	0.0000	0.7081	0.2044	0.0875	5.022	2.652	2.371	0.0892	0.9272	0.3622	0.5650	0.5650	0.6378	0.0728	0.5650	2.860	3.947	2.162	5.718	3.723	5.022	3.556
77	3	2	2	0.2740	0.5341	0.1920	0.0000	6.057	3.882	2.175	0.1657	1.0000	0.4596	0.5404	0.5404	0.5404	0.0000	0.5404	3.882	5.170	2.787	6.816	4.029	6.057	4.029
78	3	2	2	0.3062	0.5563	0.1375	0.0000	6.022	3.850	2.171	0.1660	1.0000	0.4632	0.5368	0.5368	0.5368	0.0000	0.5368	3.850	5.169	2.712	6.761	4.049	6.022	4.049
79	3	2	2	0.1766	0.7684	0.0550	0.0000	5.428	3.120	2.307	0.1157	1.0000	0.3903	0.6097	0.6097	0.6097	0.0000	0.6097	3.120	4.634	2.151	5.941	3.790	5.428	3.790
80	3	2	2	0.0000	0.6201	0.1985	0.1814	4.750	2.381	2.369	0.0818	0.8491	0.3722	0.4770	0.4770	0.6278	0.1509	0.4770	2.804	3.616	2.170	5.642	3.779	4.750	3.472
81	3	2	3	0.0000	0.4801	0.5199	0.0000	6.008	3.740	2.268	0.1394	1.0000	0.4053	0.5947	0.5947	0.5947	0.0000	0.5947	3.740	4.891	2.955	6.773	3.818	6.008	3.818
82	3	2	3	0.0756	0.3181	0.6063	0.0000	6.511	4.348	2.163	0.1795	1.0000	0.4345	0.5655	0.5655	0.5655	0.0000	0.5655	4.348	5.396	3.543	7.372	3.829	6.511	3.829
83	4	1	0	0.4836	0.2775	0.2389	0.0000	7.013	5.104	1.909	0.2649	1.0000	0.5262	0.4738	0.4738	0.4738	0.0000	0.4738	5.104	6.130	3.965	7.996	4.032	7.013	4.032
84	4	1	0	0.0000	0.2166	0.3468	0.4366	4.338	2.014	2.324	0.0786	0.6368	0.4066	0.2302	0.2302	0.5934	0.3632	0.2302	3.163	3.276	2.963	6.222	3.919	4.338	3.258
85	4	1	0	0.0000	0.4594	0.4286	0.1120	5.415	3.110	2.305	0.1142	0.9068	0.4152	0.4916	0.4916	0.5848	0.0932	0.4916	3.429	4.203	2.776	6.444	3.947	5.415	3.669
86	4	1	0	0.1237	0.4983	0.2338	0.1442	5.228	2.940	2.288	0.1138	0.8800	0.4310	0.4490	0.4490	0.5690	0.1200	0.4490	3.341	4.156	2.559	6.262	4.025	5.228	3.704
87	4	1	0	0.1875	0.4457	0.3668	0.0000	6.234	4.061	2.173	0.1705	1.0000	0.4516	0.5484	0.5484	0.5484	0.0000	0.5484	4.061	5.240	3.091	7.057	3.966	6.234	3.966
88	4	1	0	0.0000	0.3349	0.3613	0.3038	4.713	2.389	2.325	0.0896	0.7473	0.4164	0.3309	0.3309	0.5836	0.2527	0.3309	3.197	3.538	2.767	6.199	3.987	4.713	3.432
89	4	1	1	0.5071	0.0512	0.4417	0.0000	7.881	6.179	1.702	0.3475	1.0000	0.5124	0.4876	0.4876	0.4876	0.0000	0.4876	6.179	7.113	5.197	8.690	3.493	7.881	3.493
90	4	1	1	0.9176	0.0052	0.0772	0.0000	8.670	7.420	1.250	0.5088	1.0000	0.5814	0.4186	0.4186	0.4186	0.0000	0.4186	7.420	8.201	6.336	9.324	2.988	8.670	2.988
91	4	1	1	0.6514	0.0000	0.3486	0.0000	8.291	6.770	1.521	0.4134	1.0000	0.5312	0.4688	0.4688	0.4688	0.0000	0.4688	6.770	7.680	5.739	8.986	3.247	8.291	3.247
92	4	1	1	0.6114	0.1791	0.2095	0.0000	7.508	5.771	1.737	0.3285	1.0000	0.5497	0.4503	0.4503	0.4503	0.0000	0.4503	5.771	6.695	4.643	8.503	3.860	7.508	3.860
93	4	1	1	0.9162	0.0000	0.0838	0.0000	8.693	7.449	1.244	0.5111	1.0000	0.5799	0.4201	0.4201	0.4201	0.0000	0.4201	7.449	8.234	6.365	9.328	2.963	8.693	2.963
94	4	1	1	0.5500	0.4500	0.0000	0.0000	6.520	4.532	1.987	0.2303	1.0000	0.5308	0.4692	0.4692	0.4692	0.0000	0.4692	4.532	5.738	3.168	7.407	4.238	6.520	4.238
95	4	1	1	1.0000	0.0000	0.0000	0.0000	8.831	7.685	1.146	0.5462	1.0000	0.5940	0.4060	0.4060	0.4060	0.0000	0.4060	7.685	8.426	6.601	9.425	2.823	8.831	2.823
96	4	1	1	0.9111	0.0000	0.0889	0.0000	8.685	7.435	1.250	0.5090	1.0000	0.5790	0.4210	0.4210	0.4210	0.0000	0.4210	7.435	8.223	6.351	9.322	2.971	8.685	2.971
97	4	1	1	0.8282	0.1285	0.0433	0.0000	7.987	6.489	1.498	0.4142	1.0000	0.5878	0.4122	0.4122	0.4122	0.0000	0.4122	6.489	7.305	5.325	8.961	3.636	7.987	3.636
98	4	1	1	0.9234	0.0000	0.0766	0.0000	8.705	7.469	1.236	0.5140	1.0000	0.5811	0.4189	0.4189	0.4189	0.0000	0.4189	7.469	8.250	6.385	9.337	2.952	8.705	2.952
99	4	1	1	1.0000	0.0000	0.0000	0.0000	8.831	7.685	1.146	0.5462	1.0000	0.5940	0.4060	0.4060	0.4060	0.0000	0.4060	7.685	8.426	6.601	9.425	2.823	8.831	2.823
100	4	1	2	0.0000	0.5411	0.4589	0.0000	5.854	3.563	2.291	0.1298	1.0000	0.3972	0.6028	0.6028	0.6028	0.0000	0.6028	3.563	4.768	2.769	6.574	3.805	5.854	3.805
101	4	1	2	0.0000	0.6032	0.2443	0.1525	4.915	2.556	2.359	0.0886	0.8732	0.3811	0.4920	0.4920	0.6189	0.1268	0.4920	2.927	3.773	2.272	5.806	3.817	4.915	3.534
102	4	1	2	0.0000	0.7116	0.2858	0.0026	5.443	3.094	2.349	0.1056	0.9978	0.3653	0.6325	0.6325	0.6347	0.0022	0.6325	3.101	4.452	2.320	6.021	3.707	5.443	3.701
103	4	1	2	0.0000	0.6602	0.0685	0.2712	4.303	1.912	2.391	0.0647	0.7744	0.3397	0.4347	0.4347	0.6603	0.2256	0.4347	2.469	3.191	1.904	5.183	3.629	4.303	3.279
104	4	1	2	0.0000	0.9866	0.0134	0.0000	4.909	2.489	2.420	0.0761	1.0000	0.2895	0.7105	0.7105	0.7105	0.0000	0.7105	2.489	4.085	1.838	5.254	3.415	4.909	3.415
105	4	1	2	0.0538	0.5040	0.4423	0.0000	5.982	3.727	2.256	0.1421	1.0000	0.4145	0.5855	0.5855	0.5855	0.0000	0.5855	3.727	4.921	2.881	6.739	3.858	5.982	3.858
106	4	1	2	0.0000	0.6728	0.0720	0.2552	4.348	1.956	2.392	0.0658	0.7877	0.3404	0.4474	0.4474	0.6596	0.2123	0.4474	2.483	3.234	1.911	5.205	3.633	4.348	3.294
107	4	1	2	0.1092	0.8908	0.0000	0.0000	5.138	2.771	2.366	0.0942	1.0000	0.3421	0.6579	0.6579	0.6579	0.0000	0.6579	2.771	4.372	1.939	5.543	3.604	5.138	3.604
108	4	1	2	0.0000	0.5679	0.4321	0.0000	5.788	3.487	2.301	0.1258	1.0000	0.3930	0.6070	0.6070	0.6070	0.0000	0.6070	3.487	4.717	2.692	6.487	3.795	5.788	3.795
109	4	1	3	0.3588	0.0000	0.6412	0.0000	7.899	6.125	1.774	0.3255	1.0000	0.4713	0.5287	0.5287	0.5287	0.0000	0.5287	6.125	7.134	5.226	8.584	3.358	7.899	3.358
110	4	1	3	0.0057	0.4589	0.5354	0.0000	6.067	3.810	2.257	0.1436	1.0000	0.4089	0.5911	0.5911	0.5911	0.0000	0.5911	3.810	4.945	3.024	6.847	3.823	6.067	3.823
111	4	1	3	0.2253	0.0495	0.7171	0.0082	7.480	5.560	1.920	0.2715	0.9932	0.4555	0.5377	0.5377	0.5445	0.0068	0.5377	5.598	6.529	4.810	8.290	3.530	7.480	3.481
112	4	1	3	0.0000	0.0000	0.8881	0.1119	6.626	4.514	2.112	0.1942	0.9069	0.4421	0.4648	0.4648	0.5579	0.0931	0.4648	4.978	5.247	4.721	7.944	3.791	6.626	3.223
113	4	1	3	0.0000	0.3493	0.6507	0.0000	6.361	4.148	2.213	0.1623	1.0000	0.4160	0.5840	0.5840	0.5840	0.0000	0.5840	4.148	5.198	3.401	7.195	3.793	6.361	3.793
114	4	1	4	0.0362	0.0127	0.4169	0.5342	4.282	1.996	2.286	0.0845	0.5556	0.4223	0.1333	0.1333	0.5777	0.4444	0.1333	3.592	3.345	4.377	7.260	3.959	4.282	2.883
115	4	2	0	0.3093	0.1935	0.4972	0.0000	7.123	5.162	1.960	0.2519	1.0000	0.4859	0.5141	0.5141	0.5141	0.0000	0.5141	5.162	6.153	4.226	8.043	3.817	7.123	3.817
116	4	2	0	0.4105	0.1397	0.4498	0.0000	7.423	5.570	1.853	0.2910	1.0000	0.5039	0.4961	0.4961	0.4961	0.0000	0.4961	5.570	6.528	4.598	8.335	3.738	7.423	3.738
117	4	2	0	0.3076	0.4775	0.0695	0.1455	5.371	3.156	2.215	0.1362	0.8790	0.4720	0.4070	0.4070	0.5280	0.1210	0.4070	3.591	4.443	2.602	6.453	4.199	5.371	3.850
118	4	2	0	0.0000	0.3871	0.2737	0.3392	4.464	2.120	2.345	0.0783	0.7179	0.3956	0.3222	0.3222	0.6044	0.2821	0.3222	2.953	3.341	2.476	5.851	3.883	4.464	3.375
119	4	2	0	0.4506	0.4844	0.0651	0.0000	6.332	4.269	2.063	0.2037	1.0000	0.5053	0.4947	0.4947	0.4947	0.0000	0.4947	4.269	5.519	2.992	7.166	4.174	6.332	4.174
120	4	2	1	0.5761	0.0000	0.3041	0.1198	7.141	5.375	1.766	0.3093	0.9003	0.5628	0.3375	0.3375	0.4372	0.0997	0.3375	5.970	6.159	5.655	8.781	4.041	7.141	3.126

121	4	2	1	**0.9712**	0.0288	0.0000	0.0000	8.645	7.424	1.221	0.5176	1.0000	0.5962	0.4038	0.4038	0.4038	0.0000	0.4038	7.424	8.164	6.332	9.358	3.026	8.645	3.026
122	4	2	1	**0.5508**	0.3239	0.1253	0.0000	6.925	5.026	1.899	0.2658	1.0000	0.5399	0.4601	0.4601	0.4601	0.0000	0.4601	5.026	6.086	3.782	7.912	4.130	6.925	4.130
123	4	2	1	**0.5113**	0.1347	0.3540	0.0000	7.558	5.783	1.775	0.3179	1.0000	0.5249	0.4751	0.4751	0.4751	0.0000	0.4751	5.783	6.716	4.753	8.490	3.738	7.558	3.738
124	4	2	1	**0.5313**	0.4352	0.0336	0.0000	6.548	4.560	1.989	0.2306	1.0000	0.5283	0.4717	0.4717	0.4717	0.0000	0.4717	4.560	5.749	3.228	7.447	4.219	6.548	4.219
125	4	2	1	**0.9165**	0.0522	0.0312	0.0000	8.450	7.136	1.315	0.4831	1.0000	0.5917	0.4083	0.4083	0.4083	0.0000	0.4083	7.136	7.898	6.032	9.253	3.221	8.450	3.221
126	4	2	1	**0.8675**	0.1325	0.0000	0.0000	8.025	6.560	1.465	0.4256	1.0000	0.5958	0.4042	0.4042	0.4042	0.0000	0.4042	6.560	7.360	5.381	9.006	3.625	8.025	3.625
127	4	2	1	**0.8532**	0.1266	0.0201	0.0000	8.030	6.558	1.472	0.4235	1.0000	0.5923	0.4077	0.4077	0.4077	0.0000	0.4077	6.558	7.362	5.390	9.002	3.612	8.030	3.612
128	4	2	2	0.1544	**0.6830**	0.1626	0.0000	5.608	3.323	2.285	0.1256	1.0000	0.4056	0.5944	0.5944	0.5944	0.0000	0.5944	3.323	4.736	2.359	6.208	3.850	5.608	3.850
129	4	2	2	0.0000	**0.7449**	0.0000	0.2551	4.238	1.833	2.405	0.0603	0.7878	0.3179	0.4699	0.4699	0.6821	0.2122	0.4699	2.326	3.125	1.786	5.003	3.535	4.238	3.217
130	4	2	2	0.1550	**0.7139**	0.0017	0.1294	4.883	2.545	2.338	0.0931	0.8924	0.3858	0.5066	0.5066	0.6142	0.1076	0.5066	2.852	3.958	2.009	5.594	3.812	4.883	3.585
131	4	2	2	0.0964	**0.9036**	0.0000	0.0000	5.107	2.733	2.373	0.0917	1.0000	0.3356	0.6644	0.6644	0.6644	0.0000	0.6644	2.733	4.337	1.923	5.503	3.580	5.107	3.580
132	4	2	2	0.0795	**0.6931**	0.2274	0.0000	5.542	3.226	2.316	0.1163	1.0000	0.3869	0.6131	0.6131	0.6131	0.0000	0.6131	3.226	4.614	2.350	6.132	3.782	5.542	3.782
133	4	2	2	0.0052	**0.7776**	0.2172	0.0000	5.318	2.952	2.366	0.0985	1.0000	0.3507	0.6493	0.6493	0.6493	0.0000	0.6493	2.952	4.376	2.183	5.833	3.650	5.318	3.650
134	4	2	2	0.0740	**0.9260**	0.0000	0.0000	5.053	2.667	2.386	0.0875	1.0000	0.3241	0.6759	0.6759	0.6759	0.0000	0.6759	2.667	4.276	1.896	5.434	3.537	5.053	3.537
135	4	2	2	0.0000	**0.9953**	0.0000	0.0047	4.872	2.449	2.423	0.0746	0.9961	0.2863	0.7098	0.7098	0.7137	0.0039	0.7098	2.459	4.042	1.820	5.216	3.404	4.872	3.396
136	4	2	2	0.0000	**0.5188**	0.2930	0.1882	4.907	2.562	2.345	0.0911	0.8434	0.3963	0.4471	0.4471	0.6037	0.1566	0.4471	3.037	3.734	2.419	5.953	3.890	4.907	3.534
137	4	2	2	0.0000	**0.9831**	0.0169	0.0000	4.915	2.496	2.420	0.0765	1.0000	0.2907	0.7093	0.7093	0.7093	0.0000	0.7093	2.496	4.089	1.843	5.263	3.420	4.915	3.420
138	4	2	2	0.3217	**0.6783**	0.0000	0.0000	5.722	3.503	2.219	0.1458	1.0000	0.4430	0.5570	0.5570	0.5570	0.0000	0.5570	3.503	4.969	2.337	6.326	3.989	5.722	3.989
139	4	2	2	0.2292	**0.5315**	0.2393	0.0000	6.032	3.838	2.194	0.1600	1.0000	0.4500	0.5500	0.5500	0.5500	0.0000	0.5500	3.838	5.113	2.794	6.788	3.994	6.032	3.994
140	4	2	2	0.0414	**0.7595**	0.1992	0.0000	5.375	3.026	2.349	0.1039	1.0000	0.3631	0.6369	0.6369	0.6369	0.0000	0.6369	3.026	4.457	2.210	5.904	3.695	5.375	3.695
141	4	2	2	0.0000	**1.0000**	0.0000	0.0000	4.885	2.462	2.423	0.0749	1.0000	0.2852	0.7148	0.7148	0.7148	0.0000	0.7148	2.462	4.068	1.821	5.220	3.399	4.885	3.399
142	4	2	2	0.0000	**0.8739**	0.1261	0.0000	5.121	2.727	2.393	0.0873	1.0000	0.3238	0.6762	0.6762	0.6762	0.0000	0.6762	2.727	4.232	2.007	5.553	3.547	5.121	3.547
143	4	2	3	0.1737	0.0000	**0.8263**	0.0000	7.676	5.766	1.909	0.2791	1.0000	0.4308	0.5692	0.5692	0.5692	0.0000	0.5692	5.766	6.811	4.976	8.334	3.358	7.676	3.358
144	4	2	3	0.0000	0.3106	**0.5085**	0.1808	5.376	3.095	2.281	0.1184	0.8496	0.4371	0.4125	0.4125	0.5629	0.1504	0.4125	3.644	4.096	3.164	6.739	4.056	5.376	3.575
145	4	2	3	0.2968	0.0658	**0.6374**	0.0000	7.569	5.694	1.874	0.2875	1.0000	0.4699	0.5301	0.5301	0.5301	0.0000	0.5301	5.694	6.675	4.825	8.364	3.539	7.569	3.539
146	4	2	3	0.0974	0.2009	**0.7018**	0.0000	6.892	4.803	2.089	0.2089	1.0000	0.4383	0.5617	0.5617	0.5617	0.0000	0.5617	4.803	5.795	4.029	7.753	3.724	6.892	3.724
147	4	2	3	0.0548	0.2355	**0.7098**	0.0000	6.744	4.613	2.131	0.1937	1.0000	0.4298	0.5702	0.5702	0.5702	0.0000	0.5702	4.613	5.612	3.861	7.602	3.741	6.744	3.741
148	4	2	3	0.2573	0.0000	**0.7427**	0.0000	7.774	5.924	1.850	0.2993	1.0000	0.4493	0.5507	0.5507	0.5507	0.0000	0.5507	5.924	6.955	5.082	8.446	3.364	7.774	3.364
149	4	2	3	0.0153	0.4477	**0.5370**	0.0000	6.103	3.854	2.249	0.1467	1.0000	0.4123	0.5877	0.5877	0.5877	0.0000	0.5877	3.854	4.984	3.061	6.892	3.831	6.103	3.831
150	4	2	3	0.0000	0.0720	**0.5604**	0.3676	4.918	2.649	2.268	0.1074	0.6942	0.4514	0.2428	0.2428	0.5486	0.3058	0.2428	3.816	3.698	4.037	7.194	4.138	4.918	3.156
151	4	2	4	0.0000	0.3342	0.1043	**0.5615**	3.698	1.337	2.361	0.0524	0.5329	0.3269	0.2060	0.2060	0.6731	0.4671	0.2060	2.509	2.799	2.049	5.136	3.511	3.698	3.086
152	5	1	0	0.3396	0.3880	0.2724	0.0000	6.524	4.457	2.067	0.2073	1.0000	0.4906	0.5094	0.5094	0.5094	0.0000	0.5094	4.457	5.597	3.360	7.420	4.060	6.524	4.060
153	5	1	0	0.1283	0.4342	0.0327	0.4049	4.168	1.833	2.335	0.0722	0.6632	0.3835	0.2798	0.2798	0.6165	0.3368	0.2798	2.764	3.261	2.082	5.421	3.791	4.168	3.338
154	5	1	0	0.0000	0.2981	0.3859	0.3160	4.725	2.408	2.318	0.0913	0.7371	0.4215	0.3156	0.3156	0.5785	0.2629	0.3156	3.266	3.547	2.890	6.306	4.011	4.725	3.416
155	5	1	0	0.1322	0.4867	0.3811	0.0000	6.082	3.866	2.216	0.1551	1.0000	0.4345	0.5655	0.5655	0.5655	0.0000	0.5655	3.866	5.070	2.941	6.864	3.923	6.082	3.923
156	5	1	0	0.2452	0.4540	0.3008	0.0000	6.255	4.105	2.150	0.1774	1.0000	0.4636	0.5364	0.5364	0.5364	0.0000	0.5364	4.105	5.302	3.070	7.082	4.012	6.255	4.012
157	5	1	0	0.0000	0.2322	0.2921	0.4757	4.154	1.820	2.333	0.0711	0.6043	0.3895	0.2147	0.2147	0.6105	0.3957	0.2147	3.013	3.149	2.765	5.986	3.826	4.154	3.221
158	5	1	1	**1.0000**	0.0000	0.0000	0.0000	8.831	7.685	1.146	0.5462	1.0000	0.5940	0.4060	0.4060	0.4060	0.0000	0.4060	7.685	8.426	6.601	9.425	2.823	8.831	2.823
159	5	1	1	**1.0000**	0.0000	0.0000	0.0000	8.831	7.685	1.146	0.5462	1.0000	0.5940	0.4060	0.4060	0.4060	0.0000	0.4060	7.685	8.426	6.601	9.425	2.823	8.831	2.823
160	5	1	1	**0.7518**	0.0673	0.1809	0.0000	8.141	6.640	1.501	0.4165	1.0000	0.5641	0.4359	0.4359	0.4359	0.0000	0.4359	6.640	7.479	5.554	9.000	3.446	8.141	3.446
161	5	1	1	**0.6263**	0.0000	0.3737	0.0000	8.255	6.710	1.545	0.4051	1.0000	0.5262	0.4738	0.4738	0.4738	0.0000	0.4738	6.710	7.630	5.689	8.952	3.263	8.255	3.263
162	5	1	2	0.1175	**0.5679**	0.0918	0.2229	4.707	2.374	2.333	0.0887	0.8146	0.3998	0.4148	0.4148	0.6002	0.1854	0.4148	2.914	3.693	2.164	5.692	3.892	4.707	3.528
163	5	1	2	0.0000	**0.7074**	0.2926	0.0000	5.466	3.119	2.347	0.1067	1.0000	0.3662	0.6338	0.6338	0.6338	0.0000	0.6338	3.119	4.476	2.335	6.044	3.709	5.466	3.709
164	5	1	2	0.1412	**0.5866**	0.2722	0.0000	5.831	3.577	2.254	0.1391	1.0000	0.4216	0.5784	0.5784	0.5784	0.0000	0.5784	3.577	4.885	2.625	6.526	3.901	5.831	3.901
165	5	1	3	0.2539	0.0782	**0.6679**	0.0000	7.474	5.561	1.913	0.2736	1.0000	0.4622	0.5378	0.5378	0.5378	0.0000	0.5378	5.561	6.544	4.716	8.278	3.562	7.474	3.562
166	5	1	3	0.0000	0.0000	**0.6643**	0.3357	5.236	3.003	2.233	0.1247	0.7208	0.4647	0.2561	0.2561	0.5353	0.2792	0.2561	4.166	3.933	4.589	7.609	4.176	5.236	3.020
167	5	1	3	0.4173	0.0000	**0.5827**	0.0000	7.973	6.246	1.727	0.3416	1.0000	0.4837	0.5163	0.5163	0.5163	0.0000	0.5163	6.246	7.239	5.316	8.664	3.349	7.973	3.349
168	5	1	4	0.0460	0.1400	0.1245	**0.6895**	3.528	1.198	2.330	0.0528	0.4264	0.3238	0.1026	0.1026	0.6762	0.5736	0.1026	2.810	2.883	2.578	5.573	3.447	3.528	2.995
169	5	2	0	0.3597	0.3488	0.2915	0.0000	6.662	4.630	2.032	0.2204	1.0000	0.4974	0.5026	0.5026	0.5026	0.0000	0.5026	4.630	5.731	3.540	7.586	4.046	6.662	4.046
170	5	2	0	0.0000	0.4569	0.3852	0.1578	5.180	2.861	2.319	0.1044	0.8687	0.4132	0.4555	0.4555	0.5868	0.1313	0.4555	3.293	3.969	2.679	6.285	3.958	5.180	3.605
171	5	2	0	0.0000	0.4181	0.4397	0.1422	5.345	3.043	2.302	0.1128	0.8818	0.4214	0.4603	0.4603	0.5786	0.1182	0.4603	3.451	4.111	2.846	6.481	3.984	5.345	3.635
172	5	2	0	0.0445	0.4060	0.2900	0.2595	4.806	2.486	2.320	0.0937	0.7841	0.4182	0.3660	0.3660	0.5818	0.2159	0.3660	3.171	3.669	2.601	6.111	3.991	4.806	3.511
173	5	2	0	0.2538	0.2837	0.3379	0.1247	5.932	3.790	2.142	0.1699	0.8963	0.4930	0.4033	0.4033	0.5070	0.1037	0.4033	4.229	4.833	3.490	7.280	4.228	5.932	3.790
174	5	2	0	0.3681	0.0346	0.4992	0.0980	6.967	5.057	1.911	0.2615	0.9185	0.5185	0.4000	0.4000	0.4815	0.0815	0.4000	5.506	5.875	5.027	8.388	3.971	6.967	3.361
175	5	2	1	**0.5572**	0.4188	0.0241	0.0000	6.623	4.661	1.962	0.2400	1.0000	0.5354	0.4646	0.4646	0.4646	0.0000	0.4646	4.661	5.827	3.316	7.543	4.227	6.623	4.227
176	5	2	1	**0.5228**	0.4772	0.0000	0.0000	6.414	4.394	2.020	0.2182	1.0000	0.5218	0.4782	0.4782	0.4782	0.0000	0.4782	4.394	5.635	3.040	7.267	4.227	6.414	4.227
177	5	2	1	**0.7559**	0.1055	0.0000	0.1387	6.819	5.071	1.748	0.3070	0.8846	0.6034	0.2813	0.2813	0.3966	0.1154	0.2813	5.732	5.953	5.258	8.679	4.409	6.819	3.421
178	5	2	2	0.2919	**0.6440**	0.0000	0.0640	5.426	3.179	2.248	0.1302	0.9467	0.4407	0.5060	0.5060	0.5593	0.0533	0.5060	3.358	4.592	2.282	6.158	4.024	5.426	3.876
179	5	2	2	0.0000	**0.5594**	0.4406	0.0000	5.809	3.511	2.298	0.1271	1.0000	0.3944	0.6056	0.6056	0.6056	0.0000	0.6056	3.511	4.733	2.716	6.515	3.799	5.809	3.799
180	5	2	2	0.0126	**0.9874**	0.0000	0.0000	4.913	2.495	2.417	0.0769	1.0000	0.2919	0.7081	0.7081	0.7081	0.0000	0.7081	2.495	4.104	1.832	5.255	3.423	4.913	3.423
181	5	2	2	0.2522	**0.5821**	0.0000	0.1656	5.012	2.733	2.280	0.1102	0.8622	0.4335	0.4287	0.4287	0.5665	0.1378	0.4287	3.169	4.112	2.216	5.930	4.030	5.012	3.714
182	5	2	2	0.2140	**0.5670**	0.0565	0.1624	5.040	2.753	2.286	0.1093	0.8649	0.4305	0.4344	0.4344	0.5695	0.1351	0.4344	3.183	4.095	2.280	5.982	4.020	5.040	3.703
183	5	2	2	0.0155	**0.9845**	0.0000	0.0000	4.919	2.503	2.416	0.0774	1.0000	0.2935	0.7065	0.7065	0.7065	0.0000	0.7065	2.503	4.112	1.835	5.263	3.428	4.919	3.428
184	5	2	2	0.0000	**0.7447**	0.0571	0.1982	4.465	2.066	2.399	0.0679	0.8352	0.3329	0.5022	0.5022	0.6671	0.1648	0.5022	2.474	3.362	1.886	5.206	3.604	4.465	3.321
185	5	2	2	0.0000	**0.8588**	0.0386	0.1026	4.679	2.270	2.409	0.0720	0.9147	0.3168	0.5979	0.5979	0.6832	0.0853	0.5979	2.482	3.651	1.863	5.233	3.534	4.679	3.370
186	5	2	2	0.0000	**0.5233**	0.1427	0.3340	4.266	1.893	2.373	0.0671	0.7222	0.3612	0.3610	0.3610	0.6388	0.2778	0.3610	2.621	3.167	2.076	5.375	3.719	4.266	3.299

187	5	2	2	0.2111	**0.7889**	0.0000	0.0000	5.401	3.099	2.302	0.1163	1.0000	0.3924	0.6076	0.6076	0.6076	0.0000	0.6076	3.099	4.652	2.097	5.892	3.795	5.401	3.795
188	5	2	2	0.0242	**0.5400**	0.3455	0.0902	5.367	3.051	2.316	0.1108	0.9249	0.4058	0.5191	0.5191	0.5942	0.0751	0.5191	3.298	4.227	2.573	6.263	3.902	5.367	3.691
189	5	2	2	0.0000	**0.8402**	0.0000	0.1598	4.470	2.057	2.413	0.0654	0.8671	0.3115	0.5555	0.5555	0.6885	0.1329	0.5555	2.372	3.393	1.799	5.084	3.513	4.470	3.284
190	5	2	2	0.1451	**0.8549**	0.0000	0.0000	5.227	2.882	2.345	0.1015	1.0000	0.3602	0.6398	0.6398	0.6398	0.0000	0.6398	2.882	4.470	1.989	5.660	3.672	5.227	3.672
191	5	2	2	0.0000	**0.8873**	0.0000	0.1127	4.588	2.172	2.416	0.0681	0.9063	0.3059	0.6004	0.6004	0.6941	0.0937	0.6004	2.397	3.557	1.806	5.124	3.490	4.588	3.318
192	5	2	2	0.1858	**0.5738**	0.1035	0.1369	5.139	2.850	2.289	0.1114	0.8861	0.4273	0.4588	0.4588	0.5727	0.1139	0.4588	3.216	4.166	2.331	6.051	4.002	5.139	3.720
193	5	2	3	0.0000	0.0000	**0.6134**	0.3866	4.970	2.718	2.252	0.1125	0.6784	0.4580	0.2205	0.2205	0.5420	0.3216	0.2205	4.007	3.741	4.557	7.531	4.158	4.970	2.974
194	5	2	3	0.0000	0.2228	**0.6877**	0.0895	6.131	3.925	2.206	0.1573	0.9256	0.4401	0.4855	0.4855	0.5599	0.0744	0.4855	4.241	4.810	3.725	7.335	3.944	6.131	3.609
195	5	2	3	0.1265	0.2097	**0.5447**	0.1191	6.072	3.911	2.161	0.1679	0.9009	0.4722	0.4288	0.4288	0.5278	0.0991	0.4288	4.341	4.837	3.795	7.437	4.098	6.072	3.642
196	5	2	4	0.0000	0.1263	0.0000	**0.8737**	2.996	0.658	2.338	0.0346	0.2732	0.2153	0.0580	0.0580	0.7847	0.7268	0.0580	2.407	2.598	1.697	4.489	2.981	2.996	2.792
197	5	2	4	0.0000	0.1927	0.0417	**0.7656**	3.226	0.879	2.347	0.0403	0.3631	0.2613	0.1018	0.1018	0.7387	0.6369	0.1018	2.420	2.619	1.910	4.797	3.179	3.226	2.888
198	6	1	0	0.0000	0.4955	0.2647	0.2398	4.711	2.361	2.351	0.0843	0.8005	0.3928	0.4077	0.4077	0.6072	0.1995	0.4077	2.949	3.551	2.369	5.837	3.876	4.711	3.468
199	6	1	0	0.2294	0.3144	0.0964	0.3597	4.599	2.331	2.268	0.1000	0.7007	0.4468	0.2539	0.2539	0.5532	0.2993	0.2539	3.327	3.704	2.663	6.181	4.103	4.599	3.518
200	6	1	1	**0.8880**	0.0365	0.0429	0.0326	8.160	6.769	1.391	0.4516	0.9728	0.5978	0.3751	0.3751	0.4022	0.0272	0.3751	6.958	7.497	6.099	9.219	3.460	8.160	3.120
201	6	1	1	**0.7572**	0.1512	0.0000	0.0915	7.029	5.305	1.724	0.3200	0.9239	0.5984	0.3254	0.3254	0.4016	0.0761	0.3254	5.742	6.180	4.936	8.593	4.296	7.029	3.657
202	6	1	1	**0.5447**	0.3346	0.1208	0.0000	6.883	4.972	1.911	0.2613	1.0000	0.5382	0.4618	0.4618	0.4618	0.0000	0.4618	4.972	6.044	3.722	7.863	4.141	6.883	4.141
203	6	1	2	0.0000	**0.8092**	0.0000	0.1908	4.393	1.982	2.411	0.0637	0.8412	0.3144	0.5269	0.5269	0.6856	0.1588	0.5269	2.356	3.297	1.795	5.057	3.524	4.393	3.262
204	6	1	2	0.2467	**0.7533**	0.0000	0.0000	5.501	3.223	2.277	0.1252	1.0000	0.4092	0.5908	0.5908	0.5908	0.0000	0.5908	3.223	4.752	2.165	6.025	3.860	5.501	3.860
205	6	2	0	0.2719	0.3255	0.4026	0.0000	6.656	4.589	2.067	0.2102	1.0000	0.4789	0.5211	0.5211	0.5211	0.0000	0.5211	4.589	5.668	3.598	7.567	3.969	6.656	3.969
206	6	2	0	0.0926	0.4092	0.1016	0.3965	4.227	1.890	2.337	0.0734	0.6701	0.3876	0.2826	0.2826	0.6124	0.3299	0.2826	2.820	3.267	2.207	5.551	3.819	4.227	3.344
207	6	2	0	0.1084	0.2254	0.4108	0.2554	5.228	2.986	2.242	0.1237	0.7876	0.4653	0.3223	0.3223	0.5347	0.2124	0.3223	3.791	4.057	3.409	6.926	4.197	5.228	3.517
208	6	2	0	0.3992	0.4746	0.0105	0.1157	5.602	3.441	2.161	0.1569	0.9038	0.4942	0.4095	0.4095	0.5058	0.0962	0.4095	3.807	4.729	2.694	6.660	4.277	5.602	3.966
209	6	2	0	0.2312	0.2329	0.2077	0.3283	4.906	2.675	2.231	0.1176	0.7269	0.4735	0.2535	0.2535	0.5265	0.2731	0.2535	3.680	3.932	3.210	6.732	4.240	4.906	3.522
210	6	2	0	0.1302	0.2916	0.3458	0.2324	5.217	2.968	2.249	0.1221	0.8067	0.4615	0.3452	0.3452	0.5385	0.1933	0.3452	3.680	4.082	3.143	6.741	4.180	5.217	3.599
211	6	2	0	0.0000	0.3085	0.3447	0.3468	4.567	2.240	2.327	0.0847	0.7115	0.4120	0.2995	0.2995	0.5880	0.2885	0.2995	3.148	3.426	2.765	6.145	3.962	4.567	3.379
212	6	2	0	0.4103	0.1821	0.2605	0.1471	6.192	4.165	2.027	0.2084	0.8776	0.5343	0.3434	0.3434	0.4657	0.1224	0.3434	4.745	5.152	4.112	7.814	4.356	6.192	3.702
213	6	2	0	0.4565	0.2240	0.1670	0.1526	6.075	4.043	2.032	0.2043	0.8731	0.5401	0.3330	0.3330	0.4599	0.1269	0.3330	4.631	5.091	3.883	7.673	4.421	6.075	3.790
214	6	2	1	**0.6596**	0.3404	0.0000	0.0000	6.980	5.141	1.839	0.2854	1.0000	0.5622	0.4378	0.4378	0.4378	0.0000	0.4378	5.141	6.199	3.783	7.986	4.202	6.980	4.202
215	6	2	2	0.0000	**1.0000**	0.0000	0.0000	4.885	2.462	2.423	0.0749	1.0000	0.2852	0.7148	0.7148	0.7148	0.0000	0.7148	2.462	4.068	1.821	5.220	3.399	4.885	3.399
216	6	2	2	0.4484	**0.5516**	0.0000	0.0000	6.140	4.038	2.102	0.1882	1.0000	0.4950	0.5050	0.5050	0.5050	0.0000	0.5050	4.038	5.371	2.732	6.898	4.166	6.140	4.166
217	6	2	2	0.0000	**0.5143**	0.4158	0.0698	5.520	3.212	2.309	0.1165	0.9419	0.4057	0.5362	0.5362	0.5943	0.0581	0.5362	3.410	4.352	2.697	6.410	3.889	5.520	3.713
218	6	2	2	0.0000	**0.5935**	0.2964	0.1101	5.139	2.794	2.345	0.0977	0.9084	0.3882	0.5202	0.5202	0.6118	0.0916	0.5202	3.076	3.999	2.387	5.997	3.839	5.139	3.610
219	6	2	2	0.0000	**0.6437**	0.1709	0.1854	4.690	2.315	2.375	0.0789	0.8458	0.3650	0.4807	0.4807	0.6350	0.1542	0.4807	2.737	3.564	2.108	5.552	3.747	4.690	3.444
220	6	2	2	0.0056	**0.9086**	0.0750	0.0108	5.005	2.603	2.403	0.0823	0.9910	0.3141	0.6769	0.6769	0.6859	0.0090	0.6769	2.626	4.124	1.931	5.422	3.511	5.005	3.491
221	6	2	2	0.0000	**0.6195**	0.0590	0.3215	4.169	1.780	2.389	0.0612	0.7326	0.3369	0.3957	0.3957	0.6631	0.2674	0.3957	2.429	3.068	1.885	5.117	3.610	4.169	3.232
222	6	2	2	0.0000	**0.7338**	0.0674	0.1988	4.480	2.083	2.397	0.0687	0.8346	0.3361	0.4985	0.4985	0.6639	0.1654	0.4985	2.496	3.375	1.904	5.235	3.617	4.480	3.331
223	6	2	2	0.0000	**0.8690**	0.0000	0.1310	4.542	2.127	2.415	0.0670	0.8910	0.3083	0.5827	0.5827	0.6917	0.1090	0.5827	2.387	3.490	1.803	5.108	3.500	4.542	3.305
224	6	2	2	0.1476	**0.7830**	0.0694	0.0000	5.380	3.058	2.322	0.1109	1.0000	0.3803	0.6197	0.6197	0.6197	0.0000	0.6197	3.058	4.573	2.129	5.881	3.752	5.380	3.752
225	6	2	3	0.0166	0.3508	**0.5129**	0.1197	5.633	3.368	2.265	0.1296	0.9004	0.4354	0.4650	0.4650	0.5646	0.0996	0.4650	3.740	4.376	3.145	6.815	4.016	5.633	3.670
226	6	2	4	0.0000	0.4320	0.0000	**0.5680**	3.557	1.182	2.375	0.0458	0.5275	0.2948	0.2327	0.2327	0.7052	0.4725	0.2327	2.241	2.634	1.742	4.741	3.373	3.557	2.999
227	6	2	4	0.2060	0.2233	0.0000	**0.5707**	3.876	1.577	2.299	0.0705	0.5252	0.3831	0.1421	0.1421	0.6169	0.4748	0.1421	3.002	3.227	2.396	5.632	3.728	3.876	3.237
228	6	2	4	0.0000	0.0641	0.2350	**0.7008**	3.572	1.245	2.327	0.0543	0.4170	0.3317	0.0854	0.0854	0.6683	0.5830	0.0854	2.985	2.896	3.328	6.206	3.483	3.572	2.878
229	6	2	4	0.0353	0.3273	0.0000	**0.6374**	3.469	1.113	2.357	0.0464	0.4697	0.2966	0.1731	0.1731	0.7034	0.5303	0.1731	2.369	2.698	1.803	4.804	3.354	3.469	3.000
Mean				0.2907	0.3965	0.2138	0.0990	6.091	4.033	2.058	0.2030	0.9177	0.4461	0.4715	0.4715	0.5539	0.0823	0.4715	4.287	5.203	3.469	7.016	3.717	6.091	3.547
Maximum				1.0000	1.0000	0.8881	0.8737	8.831	7.685	2.423	0.5462	1.0000	0.6034	0.7148	0.7148	0.7847	0.7268	0.7148	7.685	8.426	6.601	9.425	4.421	8.831	4.238
Minimum				0.0000	0.0000	0.0000	0.0000	2.996	0.658	1.146	0.0346	0.2732	0.2153	0.0580	0.0580	0.3966	0.0000	0.0580	2.241	2.584	1.697	4.489	2.823	2.996	2.792
Probability-Weighted Avg.				—	—	—	—	—	—	—	—	—	—	—	—	—	—	—	4.395	5.491	3.358	6.949	3.720	4.459	3.591

Notes: All life expectancy (LE) estimates are 10-year LEs. Survival beyond 10 years was censored; all survivors at year 10 were assumed to die immediately thereafter. TLE is total LE; DFLE is disability-free LE; DLE is disabled LE. Pr(No FTC at Intake) is defined following eqn. (A.6); Pr(FTC at Any Time) is defined at eqn. (A.9); Joint Prob. of no FTC at intake and FTC before death) is defined at eqn. (A.15). Cond. DLE is a probability-weighted average of Cond. DLE_1 and Cond. DLE_2. Cond_DLE1 is the same as TLE for those with FTC at intake; Cond_DLE2 is defined at eqn. (A.14) for those with no FTC at intake. Age groups are: 1=49–64, 2=65–69, 3=70–74, 4=75–79, 5=80–84, and 6=85+ years. Boldface red font indicates g_{ik} > 0.5. Additional description is provided in Tables 3, S.1, and S.2 and the accompanying text.

Table A.7. Means and Standard Deviations of GoM Scores by Subtype and Subgroup

Subgroup	*N*	Subtype 1	Subtype 2	Subtype 3	Subtype 4
		Mean			
1	59	0.774	0.111	0.107	0.008
2	87	0.091	0.714	0.125	0.070
3	29	0.131	0.184	0.620	0.065
4	11	0.029	0.213	0.104	0.653
0	43	0.206	0.336	0.295	0.163
Total	229	0.291	0.397	0.214	0.099
		Standard Deviation			
1	59	0.173	0.141	0.137	0.027
2	87	0.128	0.152	0.139	0.101
3	29	0.147	0.169	0.094	0.114
4	11	0.058	0.128	0.131	0.096
0	43	0.160	0.115	0.127	0.150
Total	229	0.324	0.297	0.214	0.169
